# Primary Structure of Glycans by NMR Spectroscopy

**DOI:** 10.1021/acs.chemrev.2c00580

**Published:** 2023-01-09

**Authors:** Carolina Fontana, Göran Widmalm

**Affiliations:** † Departamento de Química del Litoral, CENUR Litoral Norte, Universidad de la República, Paysandú 60000, Uruguay; ‡ Department of Organic Chemistry, Arrhenius Laboratory, Stockholm University, S-106 91 Stockholm, Sweden

## Abstract

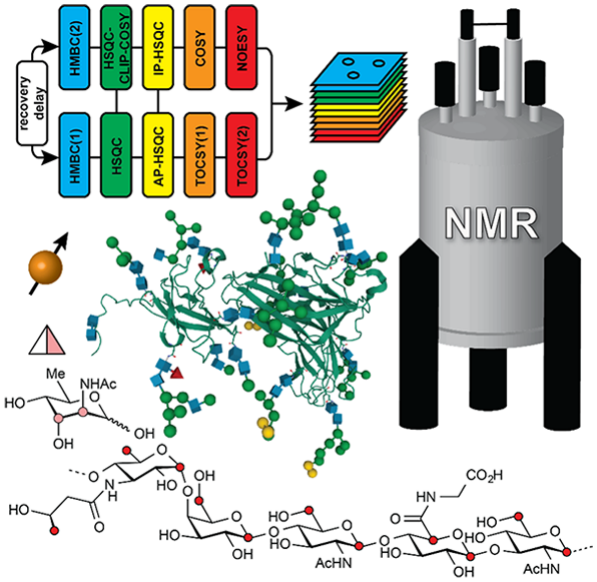

Glycans, carbohydrate molecules in the realm of biology, are present as biomedically important glycoconjugates and a characteristic aspect is that their structures in many instances are branched. In determining the primary structure of a glycan, the sugar components including the absolute configuration and ring form, anomeric configuration, linkage(s), sequence, and substituents should be elucidated. Solution state NMR spectroscopy offers a unique opportunity to resolve all these aspects at atomic resolution. During the last two decades, advancement of both NMR experiments and spectrometer hardware have made it possible to unravel carbohydrate structure more efficiently. These developments applicable to glycans include, inter alia, NMR experiments that reduce spectral overlap, use selective excitations, record tilted projections of multidimensional spectra, acquire spectra by multiple receivers, utilize polarization by fast-pulsing techniques, concatenate pulse-sequence modules to acquire several spectra in a single measurement, acquire pure shift correlated spectra devoid of scalar couplings, employ stable isotope labeling to efficiently obtain homo- and/or heteronuclear correlations, as well as those that rely on dipolar cross-correlated interactions for sequential information. Refined computer programs for NMR spin simulation and chemical shift prediction aid the structural elucidation of glycans, which are notorious for their limited spectral dispersion. Hardware developments include cryogenically cold probes and dynamic nuclear polarization techniques, both resulting in enhanced sensitivity as well as ultrahigh field NMR spectrometers with a ^1^H NMR resonance frequency higher than 1 GHz, thus improving resolution of resonances. Taken together, the developments have made and will in the future make it possible to elucidate carbohydrate structure in great detail, thereby forming the basis for understanding of how glycans interact with other molecules.

## 1 Introduction

### 1.1 Glycans in Biology

Glycans are the most abundant biomolecules found in nature and, without any known exception, they are present in all living cells either on their own (as “free” sugars) or, more commonly, covalently attached to other biomolecules to form glycoconjugates. Even though glycoproteins and glycolipids are among the most common glycoconjugates displayed on the cell surface, it has recently been demonstrated that some glycosylated small noncoding RNAs can also be found on the surface of mammalian cells.^1^ The glycan-containing biomolecules play essential roles in biological systems and are critical for the development and function of multicellular organisms, taking part in a variety of processes that involve interaction of a cell with other cells, molecules, or the environment. Glycans also play major roles in symbiotic relationships or as mediators in host–pathogen interactions, acting either as specific binding sites for viruses, bacteria, and parasites, or as antigen structures that are recognized by the host immune response.^2^

**Figure 1:**
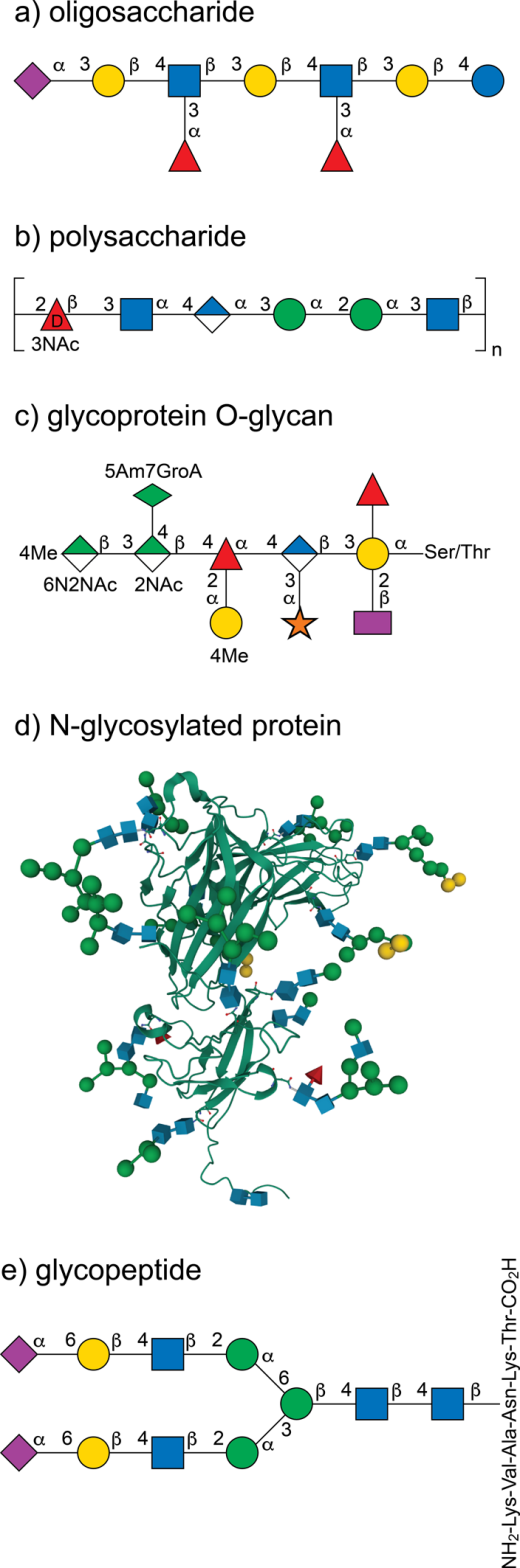
Schematic representation of glycan structures using the SNFG format (https://www.ncbi.nlm.nih.gov/glycans/snfg.html#nomn):^5,6^ (a) oligosaccharide Em-1-2-19 isolated from Asian elephant milk,^4^ (b) the repeating unit of the O-antigen polysaccharide from *E. coli* O187,^7^ (c) structure of *Tannerella forsythia* ATCC 43037 S-layer O-glycan,^8,9^ (d) structure of the highly glycosylated Epstein–Barr virus major envelope glycoprotein gp350 (PDB 2H6O)^10^ in which the N-glycans are shown using 3D-SNFG symbols,^11,12^ based on the previously developed 3D-CFG symbols,^13^ and (e) sialoglycopeptide (SGP) isolated from yolk of hen eggs.^14^

Except for lactose, structurally complex free oligosaccharides are among the main components of mammalian milk, with more than one hundred different oligosaccharide structures occurring in human breast milk. Because these oligosaccharides are minimally affected or absorbed in the gastrointestinal tract, once they reach the colon they act as prebiotics, conferring protection against pathogenic viruses or bacteria, either by shaping the microbiota of the infant or acting as soluble receptors that emulate the glycans from the gastrointestinal surface.^3^ The oligosaccharide diversity differs within mammalian species, and novel structures are continued to be reported, such as the sialylated nonasaccharide depicted in Figure 1a that was isolated from Asian elephant milk.^4^

Besides peptidoglycan, extracellular bacterial polysaccharides^15^ include lipopolysaccharides (LPS) from gram-negative bacteria,^16−18^ capsular polysaccharides (CPS),^19^ exopolysaccharides (EPS),^20^ lipoteichoic acids (LTA),^21^ wall teichoic acids (WTA),^22^ and wall polysaccharides (WPS).^23^ These polymers are considered critical virulence factors because they protect the bacteria from the host immunity and phagocytosis, and promote adherence, colonization, and biofilm formation needed for their survival. Because the structures of many of these glycans are unique to bacteria, they can also be exploited to generate carbohydrate-based vaccines. Considering that most polysaccharides are poorly immunogenic, conjugation to immunogens (such as a carrier protein) has been strategically used to stimulate the host immune response. For instance, three different conjugate vaccines against *S. pneumoniae* have been licensed since 2000, with the latest including CPS from the 13 most prevalent or invasive serotypes. These vaccines have helped to reduce the incidence of the pneumococcal disease, and it is estimated that the latter alone has prevented more than half a million deaths during the first decade of use.^24^ CPS-based conjugate vaccines against *Neisseria meningitidis* and *Haemophilus influenzae* type b have also been commercially available during the last decades. In the case of noncapsulated gram-negative bacteria, the O-antigen polysaccharide region of the LPS can be used as the main target for vaccine development; however, a detoxification procedure that involves removal of the toxic lipid A region must be performed prior to its use. Alternatively, synthetic oligosaccharides representing the O-antigen repeating units can be employed. LPS-based conjugate vaccines against some gram-negative bacteria are currently in clinical or preclinical stages.^25^ Although diseases caused by pathogenic strains of *E. coli* are usually not as severe as those caused by other pathogenic bacteria, antibiotic-resistant strains are evolving rapidly becoming an alarming public health problem; thus, the development of vaccines against these pathogens has also been targeted as a priority for the WHO. Currently, the *E. coli* serogroups are defined based on the serological reactivity of their O-antigens, and they are labeled from O1 to O188; however, only a dozen of these O-antigen structures have been considered so far in the formulation of vaccines that are currently in clinical and preclinical trials. The structure of the O-antigen polysaccharides of the latest seven recognized *E. coli* serogroups (O182–O188) were recently reported,^7,26,27^ and the hexasaccharide repeating unit of the O-antigen from *E. coli* O187 is shown in Figure 1b.

Glycosylation is also the most important co- and post-translational modification of proteins, and it has been estimated that at least half of all proteins in nature undergo this modification. Glycans can be N-, O-, or C-linked to proteins, via specific amino acid residues. In human cells, N-glycosylation takes place when a GlcNAc residue located at the reducing end of an oligosaccharide is covalently linked to the amide nitrogen of an asparagine (Asn) residue of a protein. Only ten different monosaccharides are used to build the human glycome, and seven of them (viz. d-Glc, d-Gal, d-GlcNAc, d-GalNAc, d-Man, d-Xyl, and l-Fuc) can be found directly O-linked to the hydroxyl groups of serine (Ser), threonine (Thr), tyrosine (Tyr), or hydroxylysine (Hyl) residues.^28^ Interestingly, the glycome of bacteria comprises a more diverse variety of monosaccharides; for instance, it has been shown that the proteins of the surface of *Tannerella forsythia* are heavily *O*-glycosylated with a unique decasaccharide containing, inter alia, an uncommon 5-*N*-acetamidino-7-*N*-glyceroyl derivative of pseudaminic acid (Pse) (green flat diamond in Figure 1c).^8,9^ Glycosylation of the membrane proteins of enveloped viruses contribute to shield antigenic moieties of the virus surface and, consequently, protect the virus from the immune system of the host. For instance, the viral envelope glycoprotein (gp350) of the Epstein–Barr virus (Figure 1d) is highly N-glycosylated, containing only a single glycan-free surface that corresponds to the binding epitope of this protein with the host receptor.^10^ These modifications have also represented a challenge for the development of antiviral vaccines against HIV and Ebola viruses, which display dense N- and O-glycosylated glycoproteins on their surface, respectively.^29^ Recently emerged SARS-CoV-2 also express a highly N- and O-glycosylated spike (S) glycoprotein.^30,31^ Except for proteins, bioactive peptides such as hormones and neuropeptides can also be glycosylated.^32^ Among other things, these modifications can contribute to improve the peptide stability by reducing the susceptibility to enzymatic degradation and modulate the interaction with the receptor. The sialoglycopeptide shown in Figure 1e is a natural product that has gained popularity as starting material for semisynthetic approaches of N-glycans,^33^ and can be extracted with good yields from chicken egg yolk.^34^ This glycopeptide has also been suggested to possess antibacterial properties capable of providing protection against *Salmonella* infections.^35^

### 1.2 Scope of the Review

Structural analysis of glycans by NMR spectroscopy described herein refers to the “primary structure” of carbohydrates being of natural origin, such as biological samples, or synthesized by chemical or enzymatic methods. Thus, the challenge consists of unravelling and defining sugar components, their stereochemical arrangements, linkage positions and sequence, as well as noncarbohydrate substituents. To lay the basis for the subsequent description of NMR methods used in analysis of glycans, an overview of carbohydrate structure is first given. The current review covers structural elucidation of oligo- and polysaccharides, including monosaccharide components and their substituents, and is based on NMR spectroscopy developments during the last two decades continuing from the review published in the year 2000 in this journal.^36^ Pertinent examples of glycopeptides and glycoproteins carrying oligosaccharides or polysaccharides are also included. However, glycoconjugates such as glycolipids per se that require solvents other than water, e.g., mixtures of chloroform:methanol,^37^ or pyridine^38^ will not be covered. For structural studies on saponins of steroidal or alkaloid origin, which contain carbohydrate entities, as well as flavonol glycosides, either methanol,^39^ pyridine,^40^ or dimethyl sulfoxide:water,^41,42^ are commonly used as solvents, and some specific references to compounds from these classes will be made in the context of NMR spectroscopy methodology or applications. Furthermore, solid-state NMR spectroscopy is an important technique with great potential for structure elucidation of glycans, but as it is still emerging as a tool available to the community, we refer to recent publications using this technique for glycan structure determination.^43−47^

Current complementary analytical techniques to NMR for the structural elucidation of glycans^48,49^ are, e.g., infrared spectroscopy (IR),^50,51^ liquid chromatography (LC),^52^ capillary electrophoresis (CE),^53^ and mass spectrometry (MS).^54,55^ The conformation of carbohydrates and three-dimensional (3D) structure of glycans are interlinked to the determination of the “primary structure” of a glycan molecule and some aspects and potential caveats will also be touched upon.^56−61^

## 2 Representation of Glycan Structures

### 2.1 Monosaccharides

**Figure 2:**
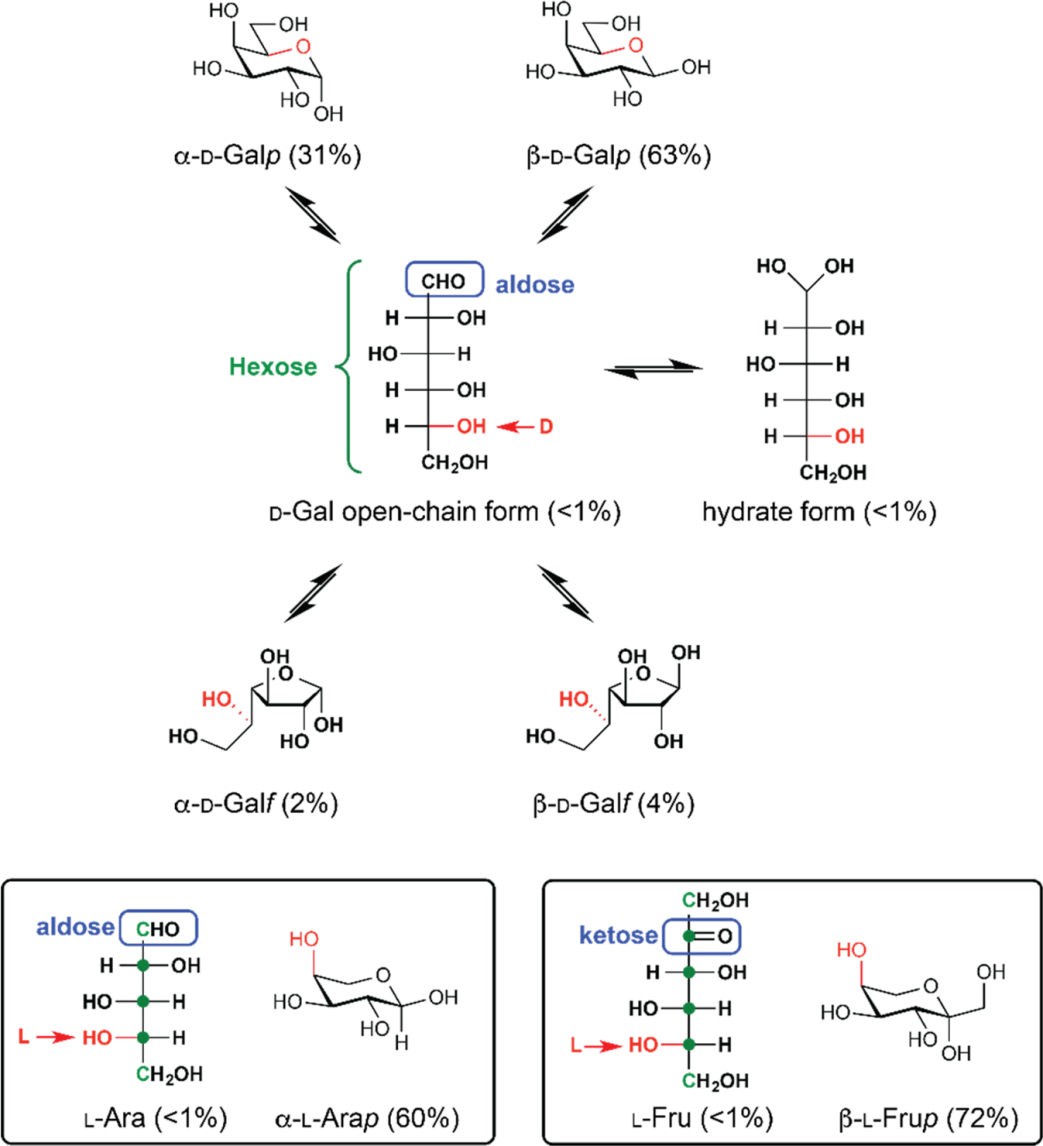
Ring–chain tautomerism of d-galactose showing the pyranose, furanose, open-chain, and hydrate forms (top). Open-chain and α-pyranose forms of l-arabinose (bottom left). Open-chain and β-pyranose forms of l-fructose (bottom right). The relative populations of each monosaccharide forms at 30 °C (d-Gal and l-Fru) and 31 °C (l-Ara) are shown in parentheses.^63−65^

Monosaccharides are polyhydroxylated compounds that can be defined as aldoses or ketoses depending on whether they have an aldehyde or a ketone group in their chain of carbon atoms, respectively. They are also classified based on their chain length, with the smallest carbohydrates consisting of three carbon atoms. Aldoses and ketoses that contain three to seven carbon atoms are, respectively, denoted trioses/triuloses, tetroses/tetruloses, pentoses/pentuloses, hexoses/hexuloses, and heptoses/heptuloses; therefore, arabinose (Ara) is considered a pentose, galactose (Gal) a hexose and fructose (Fru) a hexulose (Figure 2). The aldehyde carbon in aldoses is always numbered as C1, whereas the ketone carbon in ketoses is given the lowest possible number. Except for dihydroxyacetone, all monosaccharides have at least one asymmetric carbon, and the number of possible stereoisomers is given by 2^*n*^ (where *n* is the number of asymmetric carbons); thus, 16 different hexoses are possible. In the Fischer projection, the carbon backbone is represented vertically, with C1 on the top and the substituents that project toward the viewer depicted as horizontal bonds. In aldoses and ketoses formed up to six carbon atoms the hydroxyl group at the highest numbered asymmetric carbon is called the configurational carbon and determines the absolute configuration of each monosaccharide; when this group is pointing to the right in the Fischer projection the overall configuration is d (see Fisher projection of d-Gal in Figure 2 top), otherwise the absolute configuration is l (see Fischer projection of l-Ara and l-Fru in Figure 2, bottom left and right, respectively).^62^

Monosaccharides can exist both as open chain or cyclic compounds. The open-chain hydrates are formed by a nucleophilic addition of water to the carbonyl carbon of the free aldehyde/ketone, whereas the cyclic forms are generated by a reversible intramolecular nucleophilic addition of one of the hydroxyl groups to the aldehyde/ketone to form a cyclic hemiacetal/hemiketal (Figure 2 top). Five- and six-membered rings are the most stable structures formed from acyclic monosaccharides, and they are called furanoses and pyranoses, respectively. A new asymmetric center (termed the anomeric carbon) is generated when the cyclic tautomer is produced; thus, two possible stereoisomers can be created. If the hydroxyl group from the anomeric and configurational carbons point in the same direction in the Fisher projection, the tautomeric form is defined as the α-anomer, otherwise it is denoted as the β-anomer. In the case of free monosaccharides, all of these forms are in equilibrium in aqueous solution (Figure 2 top), and the population of each species will depend on the temperature, the monosaccharide identity, and, in the case of ionic monosaccharides, also on the pH.^66^ These equilibria have been extensively studied for pentoses, pentuloses, hexoses, hexuloses, and 6-deoxyheptoses, using NMR spectroscopy^63,67^ and/or computational methods.^68^

**Figure 3:**
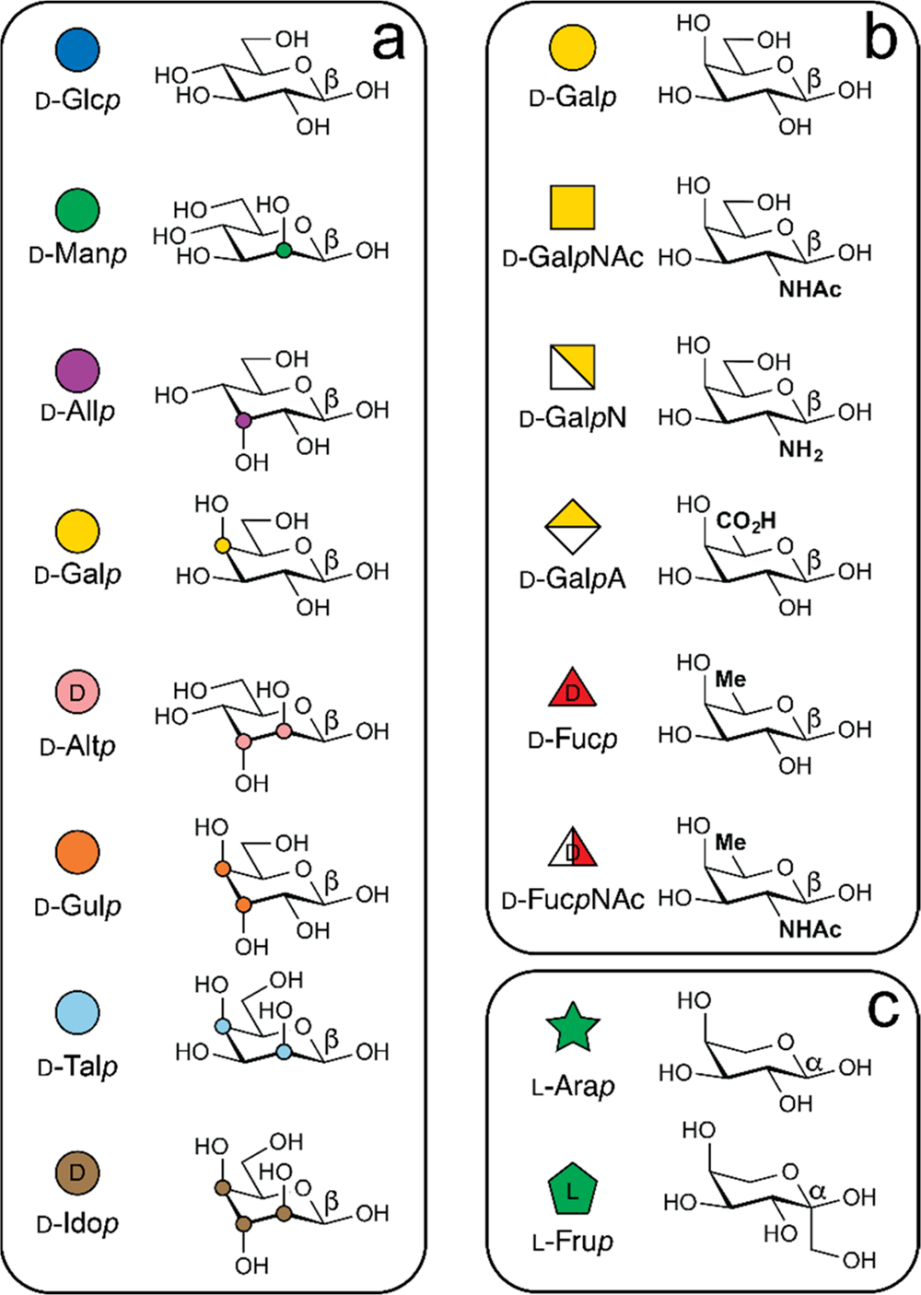
(a) The eight β-d-aldohexopyranoses (^4^*C*_1_ conformer) shown in chemical representation (right); the stereocenters that differ from those of d-glucose are highlighted using colored circles. (b) The β-anomeric and pyranose ring form of selected monosaccharides of the d-galactose series shown in chemical representation (right); the moieties that differ from those of d-galactopyranose are highlighted in bold. (c) The α-pyranose forms of l-arabinose (aldopentose) and l-fructose (ketohexose) shown in chemical representation (right). In (a–c), the corresponding monosaccharides are also represented in SNFG notation (left).

The eight possible d-aldohexoses (Figure 3a) are represented by their respective β-anomeric and pyranose ring forms and displayed in the ^4^*C*_1_ chair conformation (in which C4 and C1 are above and below the plane of the chair, respectively). In this case, the β-d-glucopyranose tautomer has all its ring substituents in equatorial orientations; because d-Man, d-All, and d-Gal are the C2, C3, and C4 epimers of d-Glc, respectively, the corresponding hydroxyl groups at their epimeric positions are therefore in axial orientation. Considering that in most cases the major number of substituents can be allocated in the less bulky equatorial orientations, the dominant conformation in d-hexopyranoses is usually the chair conformation ^4^*C*_1_, whereas l-hexopyranoses prefer the ^1^*C*_4_ conformation. However, in those cases where the number of axial bulky substituents surpasses the number of equatorial substituents other conformations can also be present. In this regard, α-d-Alt*p*, α-d-Gul*p*, α-d-Ido*p*, and β-d-Ido*p* have been shown to partially adopt the ^1^*C*_4_ chair conformation, whereas α-d-Gul*p* and α-d-Ido*p* also have minor contributions from skew conformers.^69^ Other conformations such as boat (*B*), skew (*S*), and half-chair (*H*) may also occur when some specific substituents or double bonds are present.^70^ Furanose rings are more flexible than pyranoses and can be found in different envelope (*E*) and twist conformations (*T*).^71^ A few monosaccharides, such as xylulose and sorbose, have only be found in furanose form in nature, whereas monosaccharides, such as l-Ara, d-Rib, d-Gal, and d-Fuc, can be found as both five and six-membered ring tautomers.^20^

The structural diversity of monosaccharides derivatives found in nature is increased by different modifications. When a hydroxyl group of a monosaccharide is replaced by a hydrogen atom or an amine group, deoxysugars and aminosugars are formed, respectively; the latter can be *N*-acetylated, *N*-sulfated, or remain unsubstituted. Furthermore, the hydroxyl groups can also undergo phosphorylation, sulfation, methylation, or *O*-acetylation. For instance, some lipoarabinomannans from *Mycobacterium tuberculosis* and *M. kansasii* are capped with an unusual 5-deoxy-5-methylthio-d-xylofuranose residue and its corresponding oxidized sulfoxide derivative.^72,73^ Carboxyl groups can be present and, in some cases, undergo lactonization or lactamization to nearby hydroxyl or amino groups, respectively. Some of these modifications are exemplified in Figure 3b with naturally occurring monosaccharides having the *galacto*-configuration. Moreover, when α-l-Ara*p* and α-l-Fru*p* are represented in the ^4^*C*_1_ chair conformation (Figure 3c), the hydroxyl groups located on the C1–C4 carbons are displayed in the same equatorial/axial orientation as those of β-d-Gal*p* and β-d-Fuc*p* (Figure 3b); as will be discussed in the following sections, these four analogous structures will display similar features in the NMR spectra (such as similar vicinal coupling constants patterns).

**Figure 4:**
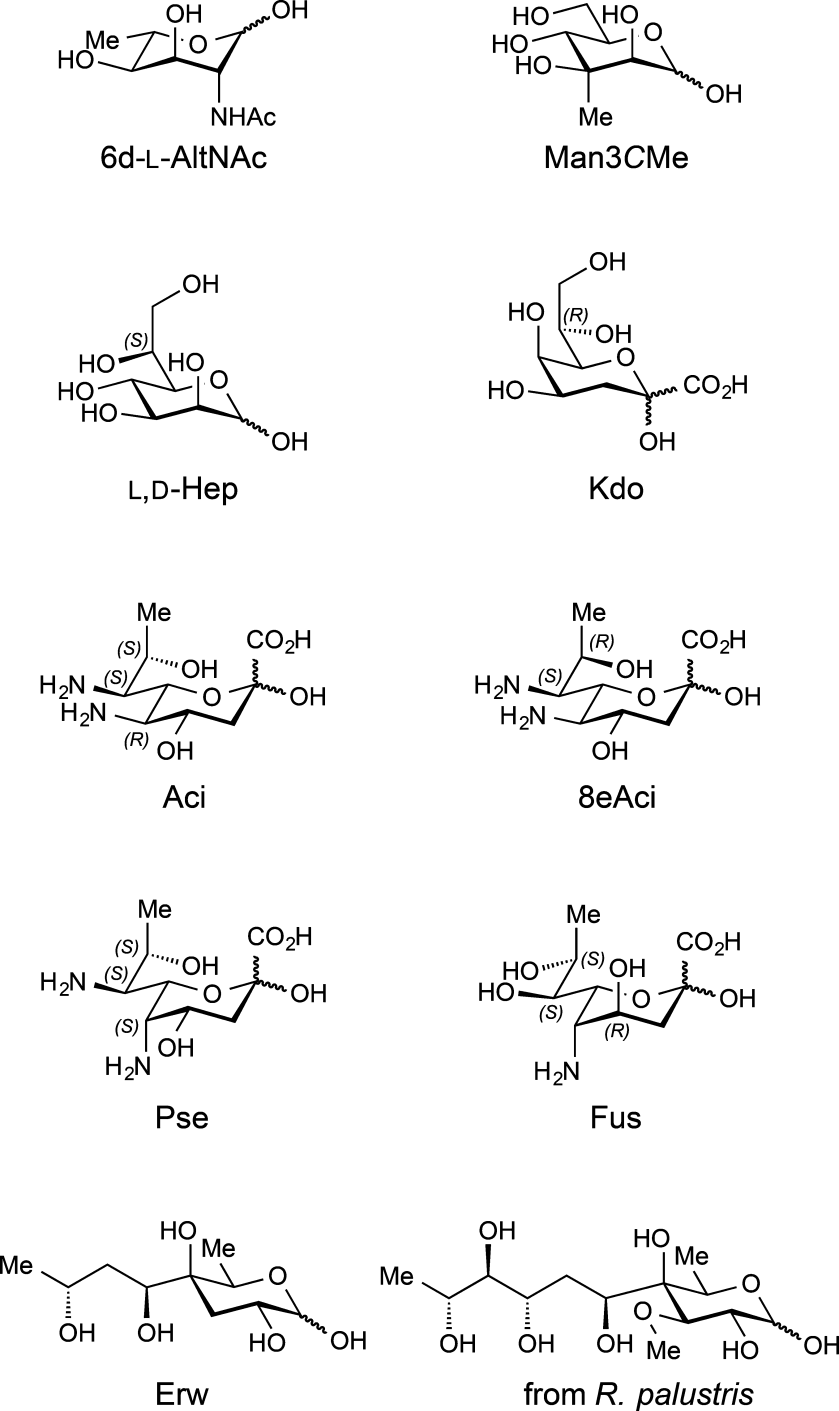
Chemical structures of the recently reported novel monosaccharides: *N*-acetyl 6-deoxy-l-altrosamine (6d-l-AltNAc),^75^ 3-*C*-methyl-d-mannose (Man3CMe),^76^ acinetaminic acid (Aci),^83^ 8-epiacinetaminic acid (8eAci),^84^ fusaminic acid (Fus),^75^ erwiniose (Erw),^77^ and C4-branched monosaccharide from *R. palustris*.^80^ Note that Aci is the C5 epimer of pseudaminic acid (Pse), where the latter was identified in 1984.^87^ Ketodeoxyoctonic acid (Kdo) and l-*glycero*-d-*manno*-heptose (Hep) are major components of the LPS core of gram-negative bacteria.

Even though more than one hundred different monosaccharides have been identified in bacterial polysaccharides, only a small number of them have been found in polysaccharides and glycoconjugates form plants and animals, with only ten of them present in the human glycome. For instance, Kdo and l,d-Hep residues are highly conserved sugar moieties found in the inner core oligosaccharides of the lipopolysaccharides from gram-negative bacteria (Figure 4). Furthermore, whereas l-rhamnose and l-fucose are ubiquitous in nature, the remaining 6-deoxyhexoses are rarer; in particular, 6-deoxy-l-idose has been reported only once in nature^74^ and both 6-deoxy-l-allose and 6-deoxy-d-idose have not been isolated from natural sources. Regarding the amino derivatives of 6-deoxy-hexoses, *N*-acetyl 6-deoxy-l-altrosamine (6d-l-AltNAc) was first isolated in 2017 from the O-antigen polysaccharide of a *Fusobacterium nucleatum* strain (Figure 4),^75^ whereas 6-deoxy-allosamine, 6-deoxy-gulosamine, and 6-deoxy-idosamine have not yet been found in nature. Besides apiose (Api), 3-*C*-methyl-branched monosaccharides are quite uncommon; Man3*C*Me was identified in 2000 as a component of the O-antigen polysaccharide of a *Helicobacter pylori* strain.^76^ Erwiniose (Erw), a novel C4-branched monosaccharide, was later obtained from the O-antigen polysaccharides of *Erwinia carotovora* and *Pectobacterium atrosepticum* strains,^77,78^ and a C4-branched higher carbon monosaccharide that shares structural similarities with caryophyllose (Car) was isolated from a *Mycobacterium marinum* lipooligosaccharide;^79^ the aforementioned monosaccharides differ in the presence and absence, respectively, of a hydroxyl group at the C3 position. Furthermore, a 3-*O*-methylated derivative of the former monosaccharide (Figure 4 right bottom) has recently been found in the O-antigen polysaccharide of a *Rhodopseudomonas palustris* strain.^80^ Interestingly, a ten-carbon bicyclic monosaccharide, namely bradyrhizose, was isolated as the only component of the O-antigen homopolysaccharide from a *Bradyrhizobium* strain.^81^ Besides Neu5Ac, Neu5Gc, and Kdn, 2-keto-3-deoxynononic acids also include rarer pseudaminic (Pse) and legionaminic (Leg) acids, as well as the C4 and C8 epimers of the latter (4eLeg and 8eLeg), fully characterized and confirmed in 2001 using a synthetic approach.^82^ Noteworthy, five novel non-2-ulosonic acids structures have been reported since 2015. Acinetaminic acid (Aci),^83^ its 8-epimer (8eAci),^84^ and the 8-epimer of Pse (8ePse)^85^ were all recently isolated from CPS of *Acinetobacter baumannii* strains. The former is the C5 epimer of Pse whereas 8eAci is the 7-epimer of Leg. Additionally, fusaminic acid (Fus) was isolated from the O-antigen polysaccharide of a *Fusobacterium nucleatum* strain and bears structural similarities with Pse, differing only in the stereochemistry at C4 and the functional group at 7 (i.e., in the former, a hydroxyl group is present at C7, whereas in the latter there is an amino group instead) (Figure 4).^75^ Furthermore, a presumed C8 epimer of the latter was isolated from another *F. nucleatum*, but its proposed configuration has not yet been confirmed.^86^

Rapid identification and comparison of monosaccharides in polymeric structures have been facilitated by the implementation of the symbol nomenclature for functional glycomics (SNFG), in which the monosaccharides are represented by colored geometric shapes.^5,6^ The shape of these symbols represents the monosaccharide type, i.e., hexoses, *N*-acetylhexosamines, and 6-deoxyhexoses are represented as fully filled circles, squares, and triangles, respectively, whereas hexosamines, hexuronic acids, and 6-deoxy-*N*-acetylhexosamines are represented as half-filled squares, diamonds, and triangles, respectively; see Figure 3b left side. In the case of aldohexose derivatives, the color of the symbol represents the relative stereochemical configuration of the monosaccharide; thus, blue, green, purple, yellow, pink, orange, light blue, and brown are used for monosaccharides with *gluco*-, *manno*-, *allo*-, *galacto*-, *altro*-, *gulo*-, *talo*-, and *ido*-configuration, respectively (Figure 3a left side). The only exception to this rule are the fucose derivatives because they have historically been represented using red color (Figure 3b left side).

### 2.2 Oligosaccharides

**Scheme 1:**
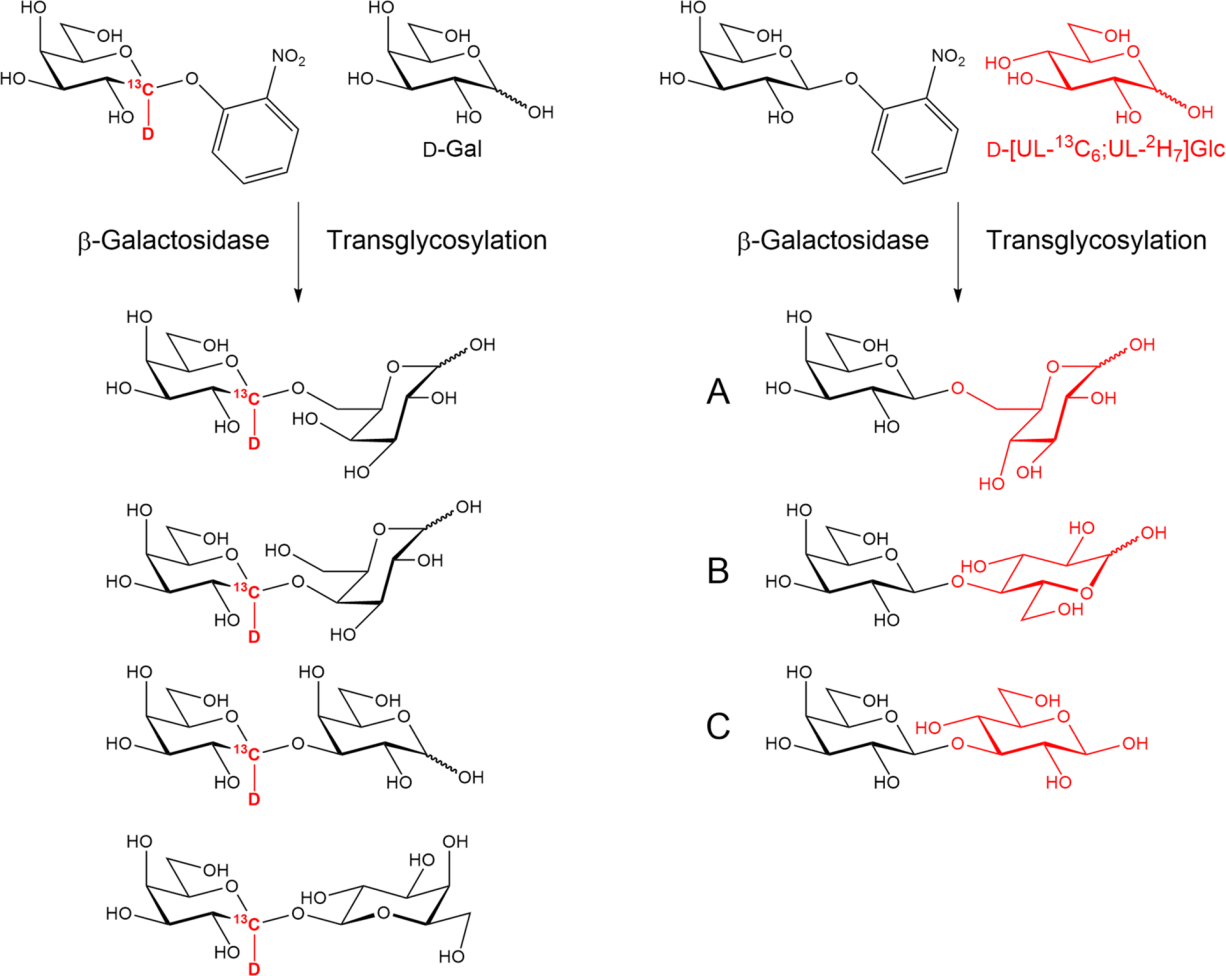
Enzymatic Transglycosylation Reactions Shown Schematically Using *ortho*-Nitrophenyl β-d-[1-^13^C;1-^2^H]galactopyranoside as the Donor and Galactose As the Acceptor (left) and *ortho*-Nitro-phenyl β-d-Galactopyranoside as Donor and d-[UL-^13^C;UL-^2^H]Glucopyranose as Acceptor (right) Isotope labeling is highlighted by red color. For the latter reaction, the disaccharide products referred to as A, B, and C have the corresponding labels for resonances from substitution positions in dDNP ^13^C NMR spectra; cf. Figure 43. Adapted with permission from refs (462 and 463). Copyright 2018 and 2020 American Chemical Society.

A disaccharide is formed when a hydroxyl group of one monosaccharide reacts with the hemiacetal/hemiketal group of another to form an acetal/ketal moiety. The newly formed carbon–oxygen bond is termed a glycosidic bond and, in biological systems, these linkages are built by a subclass of enzymes known as glycosyltransferases. The term oligosaccharide is used to refer to carbohydrate compounds that contain between two and a dozen monosaccharide residues, whereas larger structures are considered polysaccharides. Glycosyltransferases are key enzymes involved in the biosynthesis of oligo- and polysaccharides, and most of them perform their action by transferring an activated sugar moiety utilizing a nucleotide-, lipid-phosphate-, or phosphate-based donor to a sugar acceptor (a mono-, oligo-, or polysaccharide).^88−90^ In contrast, some glycosyltransferases are capable to use sucrose as nonactivated glucosyl or fructosyl donor, or accept non-natural activated donors (see *o*-nitrophenyl galactopyranoside in Scheme 1) as a convenient strategy for chemoenzymatic synthesis of oligosaccharides.^91,92^ These enzymes are not only highly specific to the aforementioned donors and acceptors, but they also display high regioselectivity toward the hydroxyl group of the acceptor and stereospecificity with respect to the resulting configuration of the anomeric carbon. A monosaccharide residue with an anomeric carbon that is not part of a glycosidic linkage in an oligosaccharide is referred to as the reducing end residue (see the glucose residue in the oligosaccharide of Figure 1a); in aqueous solution this residue is in an equilibrium between the different cyclic and open chain tautomeric forms. Internal (see galactose and *N*-acetylglucosamine residues in the oligosaccharide of Figure 1a) and nonreducing end monosaccharides (see sialyl and fucosyl residues in Figure 1a) are linked to other monosaccharides via glyosidic bonds; thus, they have defined tautomeric forms and anomeric configurations. If the reducing terminus of an oligosaccharide is linked to an aglycone moiety (see O- and N-glycans of panels c and e of Figure 1, respectively), this end is still referred as the reducing end because it has the potential to be released and recover its reducing capacity. Sucrose and trehalose are examples of nonreducing disaccharides in which the monosaccharide residues are linked to each other by their respective anomeric positions; furthermore, stachyose (vide infra, Figure 20) and raffinose are examples of nonreducing tetra- and trisaccharides, respectively.

The description of the primary structure of oligosaccharides comprises the identification of the component monosaccharides (viz., their identities, absolute configurations, tautomeric forms, anomeric configurations, and the presence of additional modifications), as well as their sequence in the oligomer and their linkage positions. In contrast to other biopolymers (such as nucleic acids and amino acids), the glycosidic linkage between two monosaccharides can take place in different arrangements, with the possibility to form branched structures. Considering all of these structural features, the number of possible oligosaccharides that can be generated with a given number of monosaccharide building blocks is by far larger than for any other biopolymer.^2,62^ Furthermore, once the conformation(s) adopted by each monosaccharide have been established, the global shape of an oligosaccharide can be described as a function of the torsion angles around each glycosidic linkage. For the analysis of NMR data, and in the case of aldoses, the most suitable definition of these torsion angles is as follows: ϕ = H1′−C1′−Ox−Cx and ψ = C1′−Ox−Cx−Hx, where the primed numbers denote atoms of the monosaccharide located toward the nonreducing end, and the letter x denotes the linkage position. In the case of (1→6)-linkages between aldohexopyranoses, the latter torsion angle is defined as ψ = C1′–O6–C6–C5, and an additional torsion angle definition ω = O6–C6–C5–O5 is required. Even though oligosaccharides are considered highly flexible structures, some preferred conformations around these torsion angles can be established. As will be exemplified below, the outcome of NMR spectra used for elucidation of sequential arrangement between sugar residues rely either on three-bond *trans*-glycosidic coupling constants or the spatial proximity of atoms located on different residues, both of which are strongly dependent on the torsion angle preferences.

Oligosaccharides composed of Glc, Gal, GlcNAc, Fuc, and/or Neu5Ac residues are one of the main components of human milk. To date, more than one hundred different oligosaccharide structures have been identified,^93^ most of which contain a lactose moiety at their reducing end, exceptions being β-d-GalNAc-(1→4)-d-Glc and β-d-Gal-(1→4)-d-GlcNAc.^94^ Lactose represent ∼85% of the carbohydrate mass in human milk, and 90% of the remaining oligosaccharides consist of a mixture of lacto-*N*-tetraose (LNT) and lacto-*N*-neotetraose (LNnT). The composition and concentration of oligosaccharides vary between different mammals, and some species have been shown to display relatively low ratios of lactose. Sulfated and UDP-oligosaccharides have been found as minor components of both human and some nonhuman mammal milk, whereas phosphorylated oligosaccharides have been found in the milk/colostrum of some herbivorous mammals.^3,94^

Osmoregulated periplasmic glucans refer to naturally occurring oligosaccharides that are produced by gram-negative bacteria. In some species, they are found as highly branched oligosaccharides consisting of 6–13 glucose residues, where the backbone and the branches are joined via β-(1→2) and β-(1→6) glycosidic linkages, respectively. They can also be found as cyclic compounds and, depending on the bacterial species, display different degrees of polymerization, type of glycosidic linkages, and substituents. In some cases, the glucose residues are linked through a variable number of β-(1→2) and β-(1→6) glycosidic linkages, whereas in other cases only β-(1→2)-linked oligosaccharides are present.^95^ Interestingly, *Ralstonia solanacearum*, *Xanthomonas campestris*, and *Rhodobacter sphaeroides* have been shown to produce cyclic glucans with a unique degree of polymerization (13, 16, and 18, respectively) containing mainly β-(1→2)-linkages in their structures.^96−98^ The enterobacterial common antigen is composed of conserved trisaccharide repeating units, produced by bacteria of the *Enterobacteriaceae* family, either as a linear polymer (ECA_LPS_ or ECA_PG_) or in a cyclic form (ECA_CYC_). In the latter case, different polymerization degrees have been observed, ranging from three to six trisaccharide repeating units. Excluding ECA_CYC-3_, which has only been identified using mass spectrometry,^99^ the other forms of ECA_CYC_ can readily be identified by their characteristic ^1^H and ^13^C NMR chemical shifts.^100,101^

### 2.3 Polysaccharides

**Figure 5:**
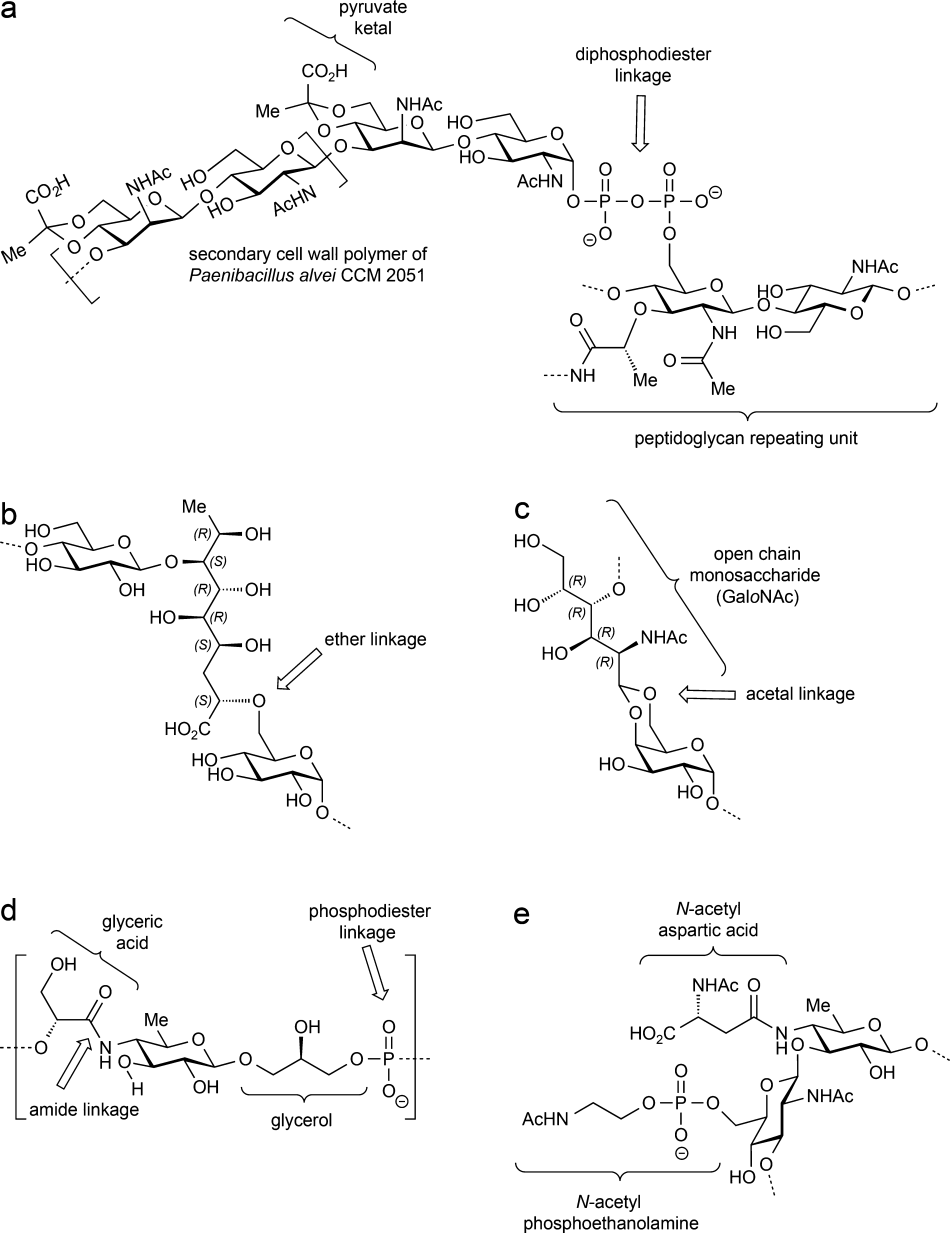
(a) Chemical structure of the secondary cell wall polymer of *Paenibacillus alvei* showing the diphosphodiester linkage to the bacterial peptidoglycan.^106^ (b) Representation of a selected region of the EPS from *Streptococcus thermophilus*, showing the residues that are connected to the 3,9-dideoxy-d-*threo*-d-altro-nononic acid moiety.^107,108^ (c) Representation of the open-chain d-GalNAc residue linked to a d-Galp residue via an acetal linkage, as present in the core oligosaccharides of *Proteus penneri* and *Shewanella oneidensis*.^109,110^ (d) Chemical structure of the WTA of *Bacillus subtilis*.^113^ (e) Selected region of the O-antigen polysaccharide of *Proteus mirabilis* O38 showing the *N*-acetyl-phosphoethanolamine and *N*-acetyl-l-aspartic acid substituents.^114^

Polysaccharides are carbohydrate polymers that contain more than a dozen monosaccharide residues, and they can be found either as homopolysaccharides (comprising only one type of monosaccharide) or heteropolysaccharides (composed of more than one type of monosaccharides). Homopolysaccharides are usually named after the monosaccharide building block they are made of (i.e., glucans, mannans, etc.), and indeed the largest synthetic polysaccharide reported to date is a branched homopolymer composed of 152 mannose residues.^102^ Many bacterial heteropolysaccharides are based on the assembly of preformed oligosaccharide units. Although most of the monosaccharide building blocks are usually connected exclusively via glycosidic bonds, in some cases phosphodiester linkages can also be present, either connecting two monosaccharide residues or a monosaccharide residue with an alditol (such as in the case of WTA and LTA). As an example, only six of the currently 182 recognized *E. coli* serogroups display O-antigen polysaccharides, in which two monosaccharides of their repeating units are linked together through phosphodiester bridges (viz., the O-antigens of serogroups O84, O152, O160, O172, O173, and O181), whereas eleven additional serogroups exhibit polysaccharides with one phosphodiester linkage between a monosaccharide residue and an alditol, such as ribitol or glycerol or glyceric acid.^26^ Furthermore, secondary cell wall polysaccharides can be anchored to the peptidoglycan via a phosphodiester or diphosphodiester linkage. Teichoic and teichuronic acids have been shown to display a specific disaccharide at their reducing end, which is connected via a phosphodiester linkage to O6 of a MurNAc residue in the peptidoglycan.^103^ The secondary cell wall polymer of *Geobacillus tepidamans* and the Lancefield group A antigen polysaccharide of group A *Streptococcus* are also attached to the peptidoglycan using this kind of linkage.^104,105^ In addition, diphosphodiester moieties have been observed to connect the reducing end of the secondary cell wall polymer of a *Paenibacillus alvei* strain directly into the bacterial peptidoglycan (Figure 5a).^106^ Only a few examples describing other kinds of linkages present in polysaccharide structures have been revealed during the last decades. For instance, an EPS from *Streptococcus thermophilus* have been described to contain a 3,9-dideoxy-d-*threo*-d-*altro*-nononic acid residue that is connected to two glucose moieties through its O7 and O2 atoms, with the latter linkage involving an ether bond (Figure 5b).^107,108^ The core oligosaccharides of *Shewanella oneidensis* and *Proteus penneri* contain an open-chain d-GalNAc residue linked through a cyclic acetal to O4 and O6 of a d-Gal*p* residue (Figure 5c) or a d-Gal*p*N residue, respectively.^109,110^ This kind of linkage had previously been described in the triterpenoid saponins Anemoclemoside A and B, in which the aldehyde group of an open-chain l-Ara residue forms a cyclic acetal with the atoms O3 and O23 of the aglycone moiety.^111,112^ Furthermore, a novel type of WTA involving an amide linkage in its backbone has been described in a *Bacillus subtilis* strain (Figure 5d).^113^

### 2.4 Glycoconjugates

Glycoconjugates are formed when a carbohydrate moiety is covalently attached to another biomolecule. Different groups can be distinguished according to the nature of the noncarbohydrate moiety, which can be a either a protein, peptide, lipid, or, as recently revealed, a small noncoding RNA. Glycosylation is the most important co- and post-translational modification of proteins, with more than fifteen different monosaccharides directly involved in the linkage to peptides and proteins.^28,115,116^ Even though N- and O-glycosylation represent the most widely distributed bonds between carbohydrates and proteins, C-mannosylation, phosphoglycosyl linkages, and glypiation have also been described. N-Glycosylation typically takes place when a GlcNAc residue is directly attached to the side-chain amino group of an asparagine (Asn) moiety of a protein (Figure 1d) or peptide (Figure 1e). Less commonly, other monosaccharides (i.e., Glc, Rha, GalNAc, and Bac) and amino acids (i.e., Arg, Lys, His, and Trp) residues can be involved in this kind of linkages.^115−119^ O-Glycosylation takes place when a monosaccharide is connected to a hydroxyl group of a serine (Ser), threonine (Thr), tyrosine (Tyr), hydroxyproline (Hyp), or hydroxylysine (Hyl). Besides GalNAc, GlcNAc, Gal, Man, Glc, Fuc, and Xyl, a few novel O-glycosidic linkages involving Pse, FucNAc, Leg, and Bac residues have recently been described in *C. jejuni*, *P. aeruginosa*, *C. botulinum*, and *N. gonorrheae* proteins, respectively.^120−123^ It has been estimated that almost one-third of the human peptide hormones are O-glycosylated.^32^ C-Mannosylation is less common and takes place when a mannosyl residue is covalently linked via its anomeric carbon to the C2 atom of the indole ring of a tryptophan (Trp) residue. This type of modification has been observed mainly in mammalian proteins, but recently its presence was confirmed in the glycoprotein of the Ebola virus and in a peptide hormone of the insect *Carausius morosus*;^124,125^ the latter has been fully characterized using NMR spectroscopy, and the conformational preferences of the mannopyranosyl residue C-linked to Trp have been investigated in different glycoproteins using a combination of molecular dynamics simulations and NMR spectroscopy data.^126,127^ Phosphoglycosylation is quite rare, but it has been reported in proteins from parasitic protozoa of the *Leshmania* and *Tripanosoma* genera; in this case, the reducing end residue of a glycan is linked to a Ser residue via phosphodiester bond. An additional kind of glycosylation has been observed in the glycopeptides sublancin and glycocin F, which are produced by *B. subtilis* and *L. plantarum*, respectively, and have been shown to display a single monosaccharide residue S-linked to a cysteine (Cys).^128,129^ Glypiation is a strategy used by eukaryotic cells to anchor proteins to the cell membrane and involves the attachment of a phosphatidylinositol-containing glycolipid (GPI) to a protein. In these structures, the C-terminal end of the protein is covalently tied to a phosphoethanolamine linker via an amide bond, whereas a phosphodiester bridge connects the linker to an oligosaccharide moiety through the O6 atom of a nonreducing end mannosyl residue; in turn, the reducing end monosaccharide of the glycan core is linked to an inositol moiety containing a phospholipid tail.^130^ The structure and dynamics of GPI analogous embedded into micelles structures have been investigated using a combination of NMR spectroscopy and molecular dynamics simulations.^131^ Lipopolysaccharides (LPS) are a particular case of glycolipids found in the external leaflet of the outer membrane of gram-negative bacteria. A smooth LPS consists of a polysaccharide structure (O-antigen) (Figure 1b) attached to a core oligosaccharide, which in turn is linked to a Lipid A moiety. The latter usually consists of a disaccharide moiety, made of GlcN or GlcN3N residues, linked to fatty acids chains through ester and/or amide bonds.^132^

## 3 NMR Spectral Characteristics of Glycans

### 3.1 NMR Active Nuclei and Spectral Ranges

NMR experiments on glycans commonly employ ^1^H, ^13^C, ^15^N, and ^31^P nuclei for detection of resonances and correlations between spins in multidimensional approaches. In ^1^H NMR spectra, nonexchangeable protons are usually found between δ_H_ ∼ 1.0–6.0 but, in H_2_O solutions, amine and amide protons can be observed at δ_H_ ∼ 8.0 and hydroxyl protons at δ_H_ ∼ 6.0–8.0. The limited spectral dispersion of proton chemical shifts usually leads to overlap of signals, and strong coupling effects may obscure the analysis in the bulk region of the ^1^H NMR spectrum. Alternatively, ^13^C resonances have a wider chemical shift dispersion (δ_C_ ∼ 15–180 ppm) and, consequently, the problem of overlap is less severe. In carbohydrates, NMR chemical shifts of ^31^P resonances are frequently found from ∼ 10 to −5 ppm (spectral region of phosphomonoester and phosphodiester groups) and ^15^N resonances can be observed at δ_N_ ∼ 30–125 (spectral region of amine, *N*-sulfate, and amide groups). Besides the direct recording of 1D ^13^C and ^31^P NMR spectra, in heteronuclear correlation experiments proton-detection is usually preferred for the analysis of carbohydrates at natural isotope abundance due to the higher sensitivity of proton nuclei. It may be noted that to obtain the maximum signal-to-noise ratio in an NMR experiment, sampling of the signal should be truncated at 1.26 times the transverse relaxation time constant *T*_2_ if it is assumed that the signal decays exponentially.^133^

In the case of naturally occurring glycans, NMR experiments are usually carried out in D_2_O solutions, and the residual HDO resonance can optionally be removed (or attenuated) using diffusion-edited experiments. Even though the best differentiation and performance (i.e., without a significant sacrifice of the signal-to-noise ratio with respect to a regular ^1^H NMR spectrum) are obtained in the case of large polysaccharides,^134^ this approach has also been employed in the analysis of oligosaccharides^135^ using both 1D ^1^H and 2D ^1^H,^1^H-TOCSY translational diffusion-filtered experiments. The strategy can also be used to remove interfering signals from low molecular weight impurities (vide infra section 5.3.3 on carbohydrate mixtures) or assign conspicuous signals that may correspond to moieties directly linked to polysaccharides and resonances from the terminal end of a polymer.^136^ In order to observe exchangeable protons of amide or hydroxyl groups, a H_2_O/D_2_O 98:2 mixture can be used as solvent instead of D_2_O; thus, a suitable water suppression scheme (e.g., presaturation, excitation sculpting, among others) has to be considered for recording proton-detected experiments. ^1^H and ^13^C NMR chemical shifts can be referenced to 3-trimethylsilyl-(2,2,3,3-^2^H_4_)-propanoate (TSP) at δ_H_ 0.0 and δ_C_ – 2.1 ppm, respectively, or to acetone.^137,138^ Alternatively, if the spectrometer sample temperature has been carefully determined, the HDO resonance can be used as a reference for ^1^H chemical shifts.^139^ Because chemical shifts of ionic compounds are pH dependent, anionic compounds or their substituents are recommended to be analyzed at pD ∼ 8–9,^140^ whereas cationic compounds may benefit from using low pH (such as detection of amino protons of GlcN, that have been shown to display the sharpest line width at pH 3.2–4.2).^141^ Furthermore, ^13^C and ^31^P NMR chemical shifts can be referenced externally using 5% (v/v) 1,4-dioxane in D_2_O (δ_C_ 67.4) and 2% (v/v) H_3_PO_4_ in D_2_O (δ_P_ 0.0), respectively. Moreover, ^15^N chemical shifts can be indirectly referenced^142^ using the TSP proton resonance as the primary reference and considering γ_N_/γ_H_ = 0.101329.^138,143^ Additional referencing strategies have been discussed previously.^144^

### 3.2 Characteristic Chemical Shifts

In the ^1^H NMR spectrum of carbohydrates, different groups of signals can be recognized: anomeric protons of aldoses resonate at δ_H_ ∼ 4.4–6.0, protons attached to carbons bearing hydroxyl or amide groups are usually found at δ_H_ ∼ 3.2–4.2, whereas methylene moieties can be observed at δ_H_ ∼ 1.6–2.8, methyl protons from *N*- or *O*-acetyl groups appear as singlets at δ_H_ ∼ 2.0–2.2, and methyl protons from 6-deoxy-hexoses as doublets at δ_H_ ∼ 1.2. Protons located at *O*-acylated positions are shifted by ∼ 0.5–1.7 ppm downfield when compared to nonsubstituted positions devoid of ester groups and may show up in the same region as the anomeric proton resonances.^134^ The signals of protons attached to carbons bearing unsubstituted and *N*-sulfated amine groups show variable chemical shifts at different pD,^66,105,145,146^ as exemplified for d-GlcN (δ_H2_ ∼ 2.6–3.3) and d-GlcN3S (δ_H2_ ∼ 1.8–3.5).^141^ The integration of the anomeric resonances can reveal the total number of monosaccharide residues, as well as the relative ratio of 6-deoxy-sugars, *N*- and/or *O*-acetylated derivatives when compared with the integrals of the corresponding methyl resonances. In H_2_O:D_2_O 98:2 mixture solutions, protons from amine and amide groups can be observed at δ_H_ ∼ 8.0, hydroxyl groups at δ_H_ ∼ 6.0–7.0 and anomeric OH at δ_H_ ∼ 7.0–8.0; because hydroxyl and amino protons exchange with the solvent at high rates, they are mainly observable at low temperatures,^141,147,148^ requiring in some cases supercooled aqueous solutions.^149^ The selection of the pH is also critical to obtain the best line width performance when observing exchangeable amino protons and anomeric OH resonances.^141^ The *cis*/*trans* isomerization of *N*-acetyl groups also has an influence on the amide proton chemical shifts as recently exemplified for d-Glc*p*NAc; the amide proton of the low populated *cis* isomer having the α-anomeric configuration is found ∼ 1 ppm upfield compared to the respective *trans* isomer, whereas for the β-anomeric configuration the chemical shift difference is ∼ 0.7 ppm.^150^

**Figure 6:**
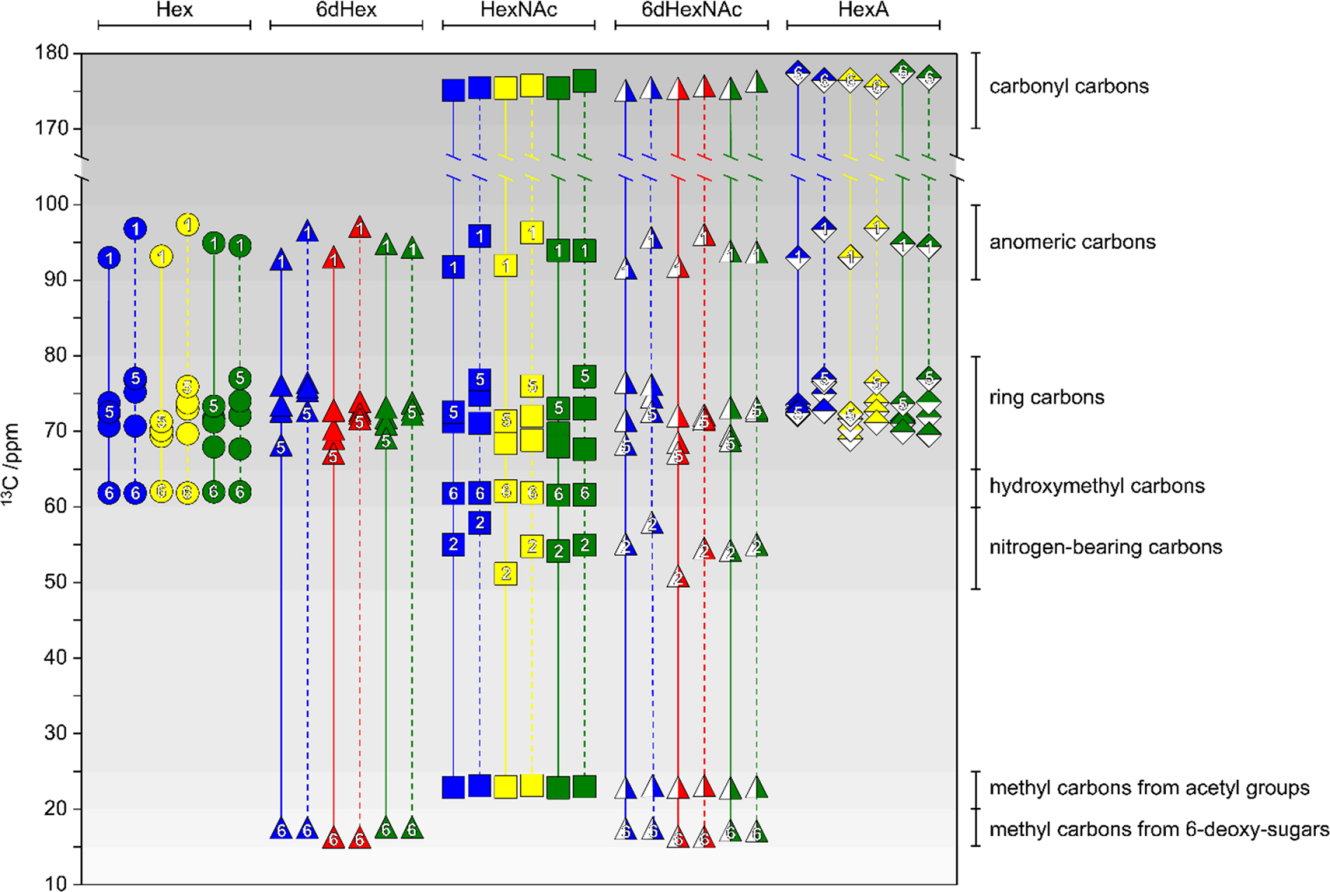
Plots of ^13^C NMR chemical shifts of common aldohexopyranoses. The marker shapes correspond to the respective monosaccharide SNFG symbols, whereas the solid and dashed lines are used to differentiate the α and β configuration, respectively. The anomeric positions (C1), C5, and C6 are annotated in all cases and nitrogen-bearing carbons (C2) are also indicated in the case of amino sugars.

^13^C NMR chemical shifts of common aldohexopyranoses are presented in Figure 6. The anomeric carbons of reducing pyranoses are found at δ_C_ ∼ 90–100, with pyranoses having the β-anomeric configuration being usually less shielded than pyranoses having the α-anomeric configuration. Carbons of secondary hydroxyl groups are observed in the spectral region δ_C_ ∼ 65–85, hydroxymethyl carbons resonate at δ_C_ ∼ 60–65, and nitrogen bearing carbons at δ_C_ ∼ 50–60. Carbonyl carbons from carboxylic acids, esters, or amide groups appear at δ_C_ ∼ 170–180, methyl carbons from acetyl groups at δ_C_ ∼ 20–25, and those from 6-deoxy-sugars at δ_C_ ∼ 15–20. In furanose rings the anomeric carbon resonances are typically less shielded than their respective pyranose counterparts as exemplified for Glc*f*, 6d-Alt*f*, 6d-Ido*f*, 6d-Ido*f*A, and All*f*NAc.^151−154^ When compared to the respective unsubstituted residues, glycosylated positions are perturbed downfield by Δδ_C_ ∼ 5–10, whereas the neighboring positions show an upfield displacement of Δδ_C_ ∼ 0–2.^155,156^

Splitting of ^13^C resonances may be evidence of the presence of phosphate groups. In the ^31^P spectrum, resonances from phosphomonoester groups are observed at δ_P_ ∼ 2–10, whereas phosphodiester groups are observed between 0 and −5 ppm. ^15^N chemical shifts from amido groups of Glc*p*NAc, Gal*p*NAc, Fuc*p*NAc, Fuc*p*3NAc, Qui*p*NAc, All*p*NAc, and All*f*NAc residues have been found in the spectral region δ_N_ ∼ 115–125.^141,143,146,154,157,158^ When the amino groups are *N*-sulfated, the chemical shifts are expected at lower chemical shifts than their respective *N*-acetylated counterparts (e.g., δ_N_ ∼ 93 in the case of *N*-sulfo-glucosamine).^146^ Furthermore, the ^15^N chemical shifts of unsubstituted amino groups are observed at δ_N_ ∼ 30, as exemplified for GlcN, GlcN6S, and GlcN3S.^141,159,160^

### 3.3 Scalar Spin–Spin Coupling Constants

Scalar spin–spin coupling constants can be employed to establish the anomeric configuration, identity, and/or conformation of pyranose residues using NMR spectroscopy. For instance, the magnitude of the ^3^*J*_H1,H2_ coupling constants have classically been used to assign the stereochemistry of the anomeric carbon of aldopyranoses that have the *gluco*-, *allo*-, *galacto*-, and *gulo*-configuration. When these residues have d or l absolute configuration, they are expected to adopt the ^4^*C*_1_ or ^1^*C*_4_ conformations, respectively; thus, a large ^3^*J*_H1,H2_ ∼ 7.8–8.5 Hz indicates that the anomeric proton is oriented axially (thus, in an antiperiplanar orientation with respect to H2), whereas a small ^3^*J*_H1,H2_ ∼ 3.7 Hz implies that the anomeric proton is orientated equatorially (*gauche* orientation with respect to H2), defining the β- and α-anomeric configuration, respectively. Nevertheless, this approach is less suitable for residues with the *manno*-, *altro*-, *talo*-, and *ido*-configuration, where the H2 atoms are found in an equatorial orientation. In these cases, the ^1^*J*_C1,H1_ couplings (cf. NMR experiments below) can be used to assess the configuration of the anomeric carbons because their magnitudes are inversely influenced by the length of the carbon–proton bond, which depends on its *s*-character, as a result of the axial/equatorial orientation of the anomeric proton. Axially oriented C1–H1 bonds are longer due to a vicinal lone-pair effect^161^ than the respective equatorial ones and, as a consequence, in the aforementioned examples the β-anomeric configuration displays smaller ^1^*J*_C1,H1_ couplings (∼ 160 Hz) than those having the α-anomeric configuration (∼ 170 Hz). However, because residues with the α-d-*ido*-configuration tend to prefer the ^1^*C*_4_ conformation instead of the ^4^*C*_1_ conformation, a smaller ^1^*J*_C1,H1_ value of ∼160 Hz is observed for the α-anomeric configuration.^162^ This has also been observed in the case of l-IdoA and 6d-l-Ido, where the magnitude of the ^1^*J*_C1,H1_ couplings are consistent with axially oriented anomeric protons in both α- and β-anomeric forms in pyranose residues.^152^ Furanoses are generally more flexible than pyranoses, and the anomeric protons are typically displayed in pseudoaxial orientations, independently of the configuration of the anomeric carbons; consequently, the one-bond carbon–proton couplings are less variable, with differences < 4 Hz between the α- and β-furanose forms of a given monosaccharide (with values in the range 170–180 Hz).^152,154,162^ Furthermore, the one-bond carbon–proton couplings of nonanomeric atoms are ∼ 145 Hz on average;^151,163,164^ this information has to be taken into consideration when setting up the delays for optimum magnetization transfer from protons to directly attached carbons in NMR experiments that employ INEPT transfer schemes, or when one-bond carbon–proton couplings are to be suppressed using low-pass filters.

**Figure 7:**
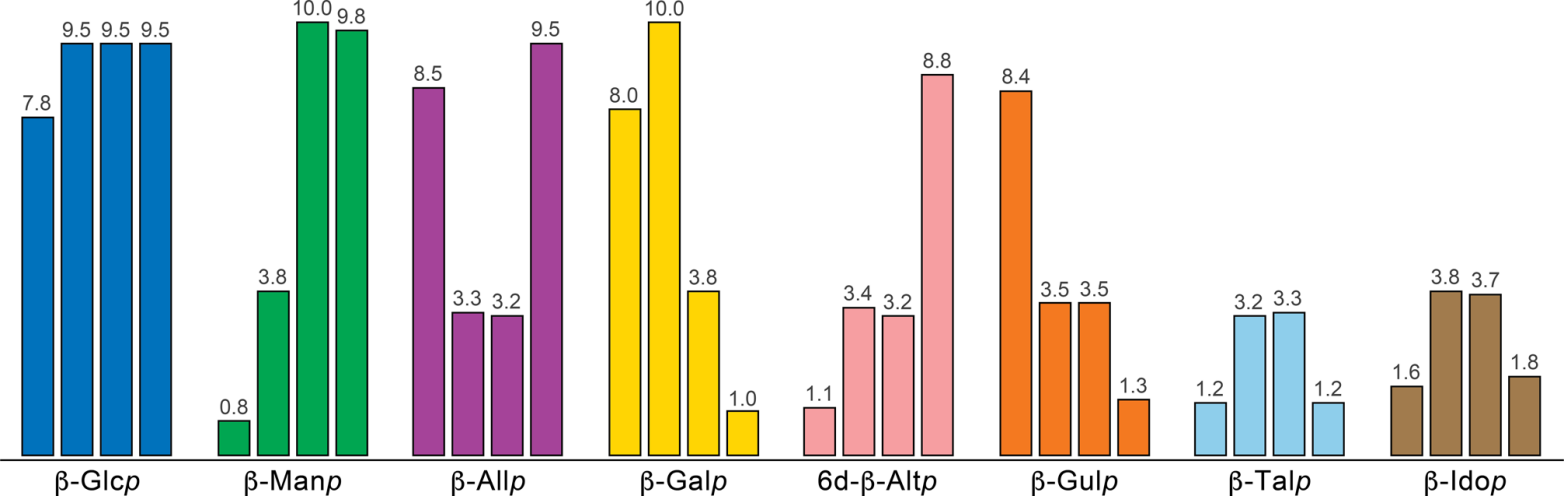
Representation of the ^3^*J*_H1,H2_, ^3^*J*_H2,H3_, ^3^*J*_H3,H4_, and ^3^*J*_H4,H5_ coupling constant values (left to right, respectively) of β-anomeric and pyranose ring forms of selected monosaccharides with *gluco*-, *manno*-, *allo*-, *galacto*-, *altro*-, *talo*-, and *ido*-configurations using bar charts.^153,162,165−167^ The coupling constants values (Hz) are indicated at the top of each bar.

**Figure 8:**
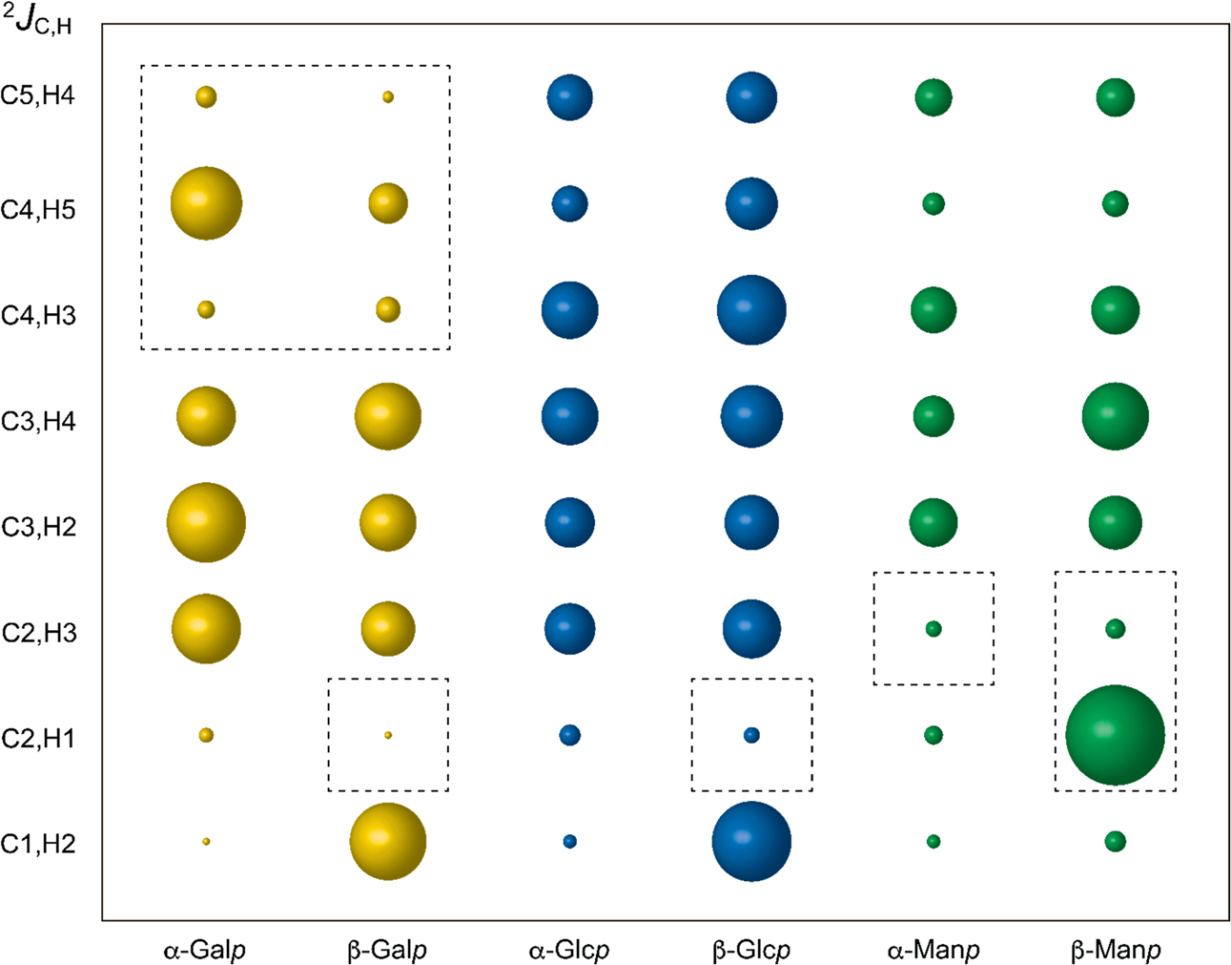
Representation of ^2^*J*_CH_ of the α- and β-anomeric and pyranose ring forms of (^4^*C*_1_ conformer) of galactose, glucose, and mannose, where the magnitude of the coupling constants values correlate with the width of the bubbles.^170^ The dashed lines indicate ^2^*J*_CH_ with positive signs.

When establishing the identity of pyranose residues, a useful approach is to consider the monosaccharides in groups, according to the relative configuration of their asymmetric carbons. Monosaccharides with *gluco*-, *manno*-, *allo*-, *galacto*-, *altro*-, *gulo*-, *talo*-, and *ido*-configurations have a characteristic set of ^3^*J*_H1,H2_, ^3^*J*_H2,H3_, ^3^*J*_H3,H4_, and ^3^*J*_H4,H5_ coupling constant values, as illustrated for selected β-anomeric forms in pyranoses (Figure 7).^153,162,165−167^ These coupling constants can also be used to assess the conformation of pyranose rings and, when deviations from canonical three-dimensional structures are observed, they can reveal the identity of the different conformers taking part in the conformational equilibria.^168,169^ Furthermore, the patterns of the eight possible ^2^*J*_CH_ couplings related to endocyclic pyranose carbon atoms have also proven valuable to establish the identity and anomeric configuration of residues with the *galacto*-, *gluco*-, and *manno*-configuration (Figure 8); this distinction can be made either by considering the magnitude and/or the sign of the ^2^*J*_CH_ couplings profiles, and the approach has the potential to be extended to residues with other configurations.^170^ Due to the low natural abundance of ^13^C nuclei, scalar carbon–carbon coupling constants are in practice only relevant in the case of ^13^C-isotopically labeled glycans (vide infra), and an average ^1^*J*_CC_ ∼ 45 Hz is frequently observed for carbons of cyclic aldoses (with ^1^*J*_C1,C2_ ∼ 42–48 Hz and nonanomeric ^1^*J*_CC_ ∼ 37–45 Hz), whereas the magnitude of endocyclic ^2^*J*_CC_ and ^3^*J*_CC_ are commonly < 5 Hz.^171^

Transglycosidic ^3^*J*_CH_ couplings are of key relevance for sequence analysis of natural abundance glycans, whereas ^2^*J*_CC_ and ^3^*J*_CC_ couplings can be useful in the sequence analysis of ^13^C-labeled oligosaccharides.^148^ Different theoretical approaches have been developed to estimate the magnitude of these couplings based on the conformational preferences around the glycosidic linkage.^161,172^ In addition, when a phosphate group is linked to the anomeric position of aldoses, vicinal phosphorus–proton coupling constants can readily be measured from the respective anomeric proton resonances (^3^*J*_P,H1_ ∼ 6–7 Hz), and different sets of carbon–phosphorus couplings (^2^*J*_PC_ ∼ ^3^*J*_PC_ ∼ 2–8 Hz) can be obtained from the ^13^C NMR spectrum of phosphorylated glycans.^173−176^ In the case of amide groups ^1^*J*_NH_ ∼ 93 Hz, and a small temperature dependence may indicate the presence of hydrogen bonds.^177,178^ Even though *N*-acetyl groups mainly prefer the *trans*-conformation, the minor *cis*-forms of α- and β-d-Glc*p*NAc have recently been characterized in solution, with the latter displaying slightly smaller ^1^*J*_NH_ coupling constants (87–89 Hz) than the former (91–93 Hz).^150^

## 4 Identification of Structural Parts

### 4.1 Constituent Monosaccharides

Component analysis of oligo and polysaccharides requires the identification of the constituent monosaccharides. This analysis can be carried out by hydrolysis or methanolysis of the polymer to obtain a mixture of free monosaccharides or methyl glycosides, respectively, which can then be analyzed using NMR spectroscopy.^179^ Each monosaccharide will show distinctive sets of ^1^H and ^13^C resonances in the NMR spectra, corresponding to different cyclic and open chain forms. When NMR spectroscopic information on these compounds is available in literature or databases, the identity of the monosaccharide can be assigned readily, and preparation of reference samples for data comparison is not required. The intensity of each set of proton resonances will reflect the relative population of each species in the mixture; thus, integration of the anomeric resonances in the ^1^H NMR spectrum can be used to determine the relative proportion of each monosaccharide in the mixture. This strategy has gained popularity in the determination of monosaccharide contents of polysaccharides isolated from biomass because it offers an improvement in the analysis time when compared to classical chromatographic techniques such as GLC and HPLC.^180,181^ Because the residual HDO signal can interfere with the integration of the anomeric proton resonances in neutral D_2_O solution, the 3.2–4.0 ppm region of the ^1^H NMR spectrum can also be included in the analysis; in the latter case, partial least-squares models have been implemented to overcome problems due to the severe spectral overlap in the region where most of the carbohydrate resonances reside.^182^

When NMR spectroscopic information on a specific monosaccharide is not available, detailed analysis of chemical shifts and coupling constant patterns can be used to assess its identity. For instance, the structure of the bicyclic monosaccharide bradyrhizose could be established using this approach.^81^ However, signal overlap accompanied by strong coupling effects usually hamper the interpretation of the bulk region in the ^1^H NMR spectrum of monosaccharides, as well as the extraction of accurate *J*_HH_ coupling constants; thus, if the monosaccharide can be isolated and purified, NMR spin simulation may assist to retrieve information on chemical shifts and coupling constants (cf. section 5.3.2 on NMR spin simulations). Once the structure of a novel monosaccharide has been inferred from the NMR analysis, the synthesis of authentic standards may help to confirm or revise its identity, as exemplified for Leg, 4eLeg, 8eLeg, and 6d-d-Alt.^82,153,183^ Sometimes, the depolymerization process can lead to the formation of unexpected bicyclic products, which conveniently can facilitate the assignment of the relative configuration of key asymmetric carbons. Examples of bicyclic compounds are the 1,5-intramolecular lactone of β-Aci5Ac7Ac that is formed under the acidic hydrolysis conditions^83^ and the intramolecular glycoside of the 4-*C*-branched monosaccharide isolated from the hydrolysate of the O-antigen polysaccharide from *R. palustris*.^80^ Interestingly, three different types of intramolecular hemiacetal ring closures have been observed in the case of bradyrhizose involving the aldehyde group at position 1 and the hydroxyl group of positions 4 (furanose) or 5 or 9 (pyranoses).^184^

Sugar analysis of glycosides can also be performed by hydrolyzing the native material directly in the NMR tube using deuterated sulfuric acid (2 M D_2_SO_4_), as was demonstrated for a flavonoid, a saponin, and two aminoglycosides.^185^ Even though the signal-to-noise of acidic samples is reduced when compared to neutral samples (i.e., due to the increased conductivity of the sample), an advantage with this solvent is that the HDO peak resonates at ∼ 6 ppm in the ^1^H NMR spectrum. This facilitates straightforward identification of the anomeric resonances at lower chemical shifts in the spectral region ∼ 4.5–5.5 ppm as an α/β-mixture of each monosaccharide with characteristic chemical shifts, as well as their ^3^*J*_H1,H2_ coupling constants. The hydrolysis is typically carried out at an elevated temperature of ∼ 95 °C, but the duration depends to a great deal on the ease of release of the monosaccharides from the native material, as well as on the changes that may occur to the sugar residues during the strong acidic conditions, which for some sugars lead to formation of 1,6-anhydro derivatives, degradation, or complete decomposition. The optimum reaction conditions can be investigated through a time-course monitoring of the hydrolysis process directly in the NMR tube.^186^

Frequently, the identity of the monosaccharides residues can directly be assessed by analysis of NMR spectroscopic data of each monosaccharide spin system in the native oligo- or polysaccharide (vide infra section 4.3). In the case of bacterial polysaccharides, this kind of analysis is facilitated when biosynthetic information is available prior to the NMR analysis.^26,187,188^

### 4.2 Absolute Configuration

**Figure 9:**
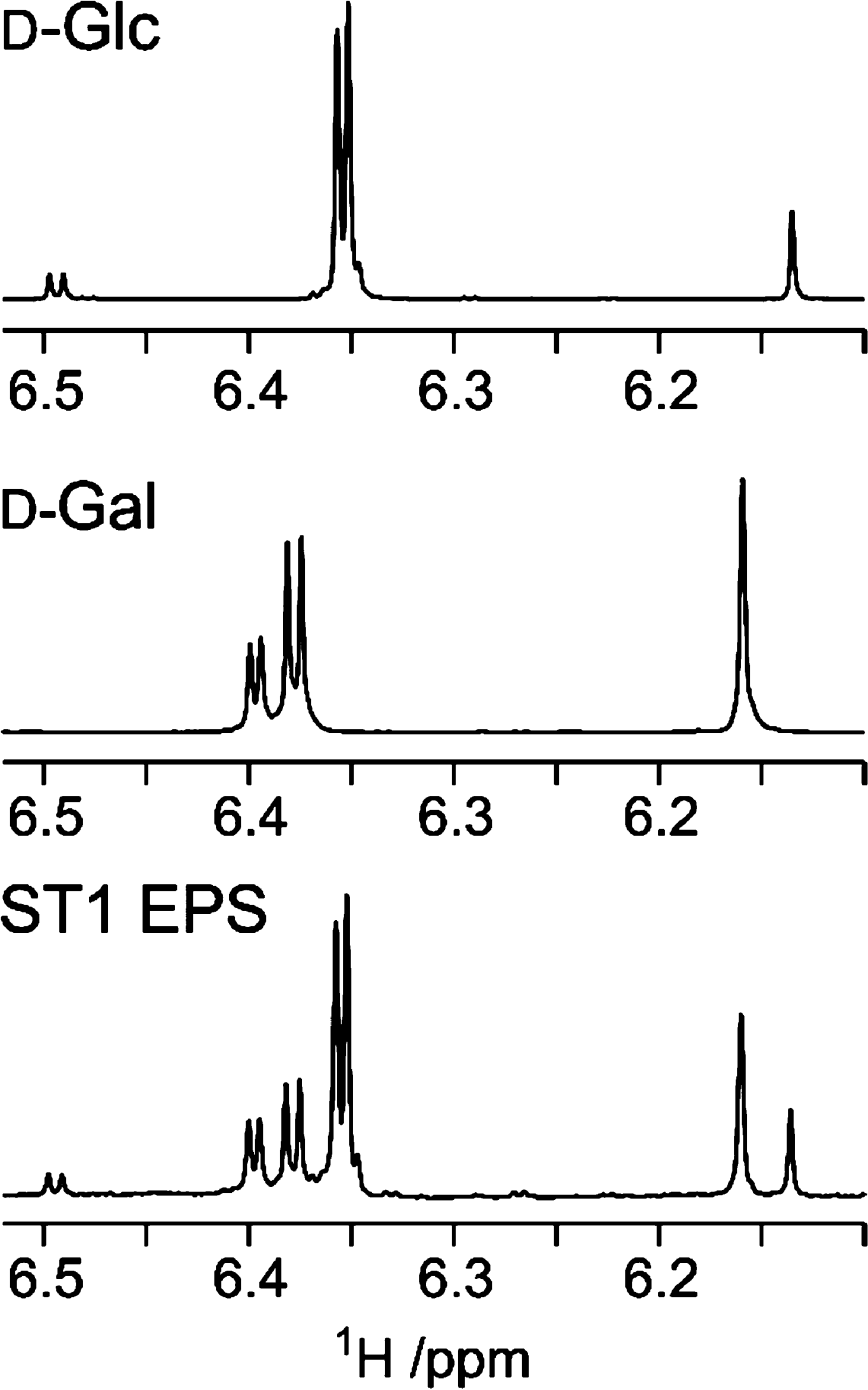
Component analysis of the ST1 exopolysaccharide (EPS) from *Streptococcus thermophilus* by derivatization with chiral (*S*)-(+)-2-methylbutyryl (SMB) groups. ^1^H NMR spectra of the EPS-SMB hydrolysate (bottom), d-galactose-SMB (middle), and d-glucose-SMB (upper). Adapted and reproduced with permission from ref (191). Copyright 2010 Springer.

The absolute configuration of monosaccharide residues can be determined by NMR spectroscopy after derivatization of the hydrolyzed glycan (∼1 mg) with an optically active reagent. These reactions usually yield a mixture of products for each monosaccharide component (i.e., pyranosides and/or furanosides in α/β-anomeric configuration), which results in a characteristic set of ^1^H and ^13^C resonances in the NMR spectra. When the same enantiomeric form of the reagent is employed in the derivatization of an enantiomeric pair of monosaccharides, or vice versa, diastereomeric products are obtained; consequently, the respective sets of NMR resonances can be used as a fingerprint to identify both the identity and absolute configuration of each component. Even though the analysis of the anomeric region of a ^1^H NMR spectrum is usually enough to perceive the chemical shift differences of diastereomeric pairs, in some cases the analysis of ^13^C chemical shifts through an ^1^H,^13^C-HSQC spectrum will offer better resolution. A derivatization process that uses (*S*)-(+)-2-methylbutyric anhydride as reagent was first proposed by York et al.^189^ and has successfully been applied in the determination of the absolute configuration of different neutral monosaccharide components (viz., d-Glc, d-Gal, l-Rha, d-Rib, d-Xyl, l-Ara, and d-GlcN) present in the EPS of *Nostoc commune* DRH-1, *S. thermophilus* ST1 (Figure 9), the O-antigen polysaccharide of *B. holmesii* strain ATCC 51541, and glycosides from *M. salicifolia* bark.^190−193^ An alternative derivatization method that involves the glycosylation of the free monosaccharides with (*R*)- or (*S*)-2-butanol was reported by Lundborg et al.,^179^ and has been employed in the absolute configuration analysis of the O-antigen polysaccharides of *E. coli* O59 and O155, and the EPS of *L. plantarum* C88.^187,194,195^ In the latter procedure, and when NMR spectroscopic data of standard derivatives are available, the analysis of the NMR data of the mixture can be carried out in a semiautomated manner using the component analysis module of the CASPER program.^179^

More recently, an in-NMR tube derivatization method was developed to determine the absolute configuration of monosaccharides whereby a hydrolysate was reacted with d- or l-cysteine methyl ester in pyridine-*d*_5_ at 60 °C for 1 h, resulting in thiazolidine derivatives.^196^ The characteristic pair of ^1^H NMR resonances from each diastereomeric derivative are in the spectral region ∼ 5–6 ppm, originating from the anomeric proton of the sugar residue, which makes it possible to determine the absolute configuration of sugars under the condition that the monosaccharides have been determined prior to the identification of their absolute configuration; the methodology was exemplified for sugar constituents of a saponin.

In some cases, when the absolute configuration of a specific monosaccharide residue of a glycan is known, the absolute configuration of the directly attached monosaccharides can be established through the analysis of ^13^C NMR glycosylation shifts.^197,198^ Interestingly, this strategy has proven useful to assign the absolute configuration of Rha residues linked to a structurally conserved N-glycan moiety of the major capsid protein of some chloroviruses. Depending on the virus species, the rhamnose residue that is linked to O3 of a trisubstituted l-Fuc*p* residue can have the d- or l-configuration; as predicted, a larger glycosylation shift (displacement) is observed for C1 of the d-Rha residue when compared to that of l-Rha.^199^ Likewise, in some cases, the absolute configuration of an aglycone moiety directly attached to a glycan can be inferred from ^1^H chemical shifts analysis, provided that the absolute configuration of the reducing end monosaccharide of the glycan is known; this approach has been explored in the analysis of marine steroid glycosides linked to β-d-Glc*p* or α-l-Ara*p* residues.^200^

### 4.3 Ring Forms, Open Form, or Alditol

**Figure 10:**
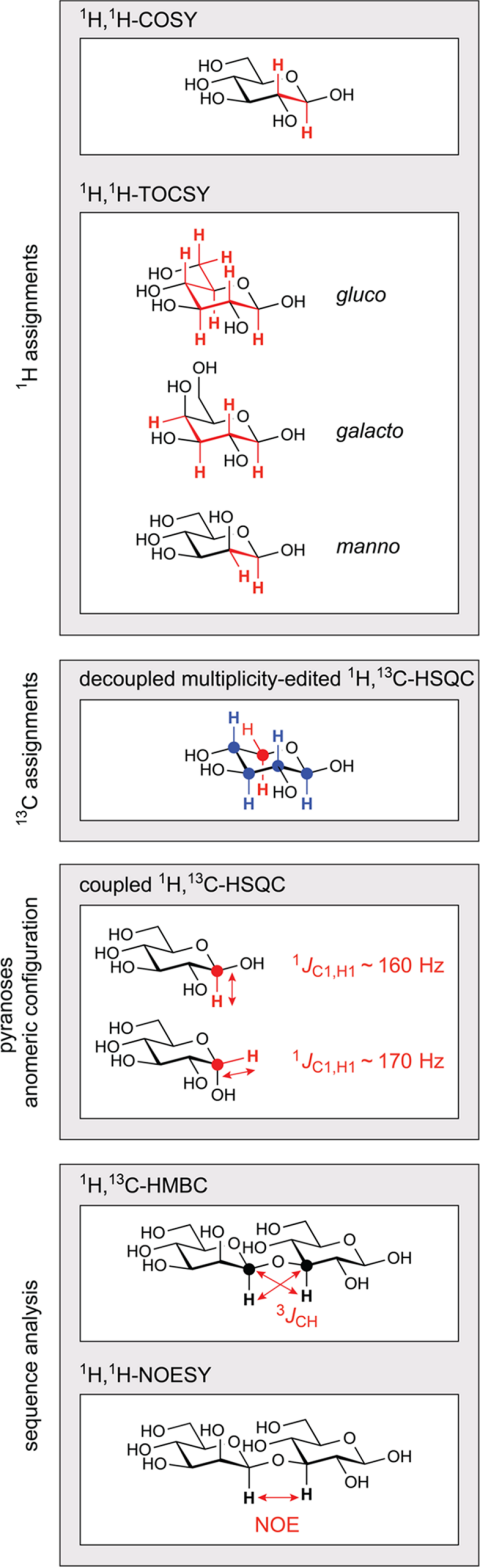
Summary of classical NMR experiments used for ^1^H and ^13^C chemical shifts assignments of carbohydrates, anomeric configuration determination, and sequence analysis; the key correlations observed in each spectrum are indicated in red and/or blue color.

Once the identity and absolute configuration of the monosaccharide components of a glycan have been established, different NMR experiments can be used to assess the form in which these residues are present in the native oligo- or polysaccharide (i.e., pyranose, furanose, or open chain). Even though ^1^H,^1^H-COSY correlations are useful to establish two- and three-bond proton–proton connectivities (Figure 10 top) from signals found in regions of the spectra devoid of spectral overlaps (such as correlations from anomeric protons, and those from methylene and methyl groups of deoxy sugars), ^1^H,^1^H-TOCSY experiments have proven more advantageous in assigning resonances in the crowded areas of the spectra, commonly found in carbohydrates. In this regard, the analysis of ^1^H,^1^H-TOCSY spectra recorded with increasing mixing times (usually in the range from 10 to 120 ms) is highly informative because the magnetization is progressively transferred from the neighboring to the most distant protons within each monosaccharide spin system. Considering that the propagation of the magnetization between protons occurs via direct scalar coupling constants, a characteristic pattern of correlations can be observed from the anomeric proton resonances of each monosaccharide depending on the magnitude of the set of ^3^*J*_HH_ coupling constants present in the spin system.^201^ For instance, in pyranose residues with the *gluco*-configuration, where all the ^3^*J*_HH_ coupling constants are large enough, complete magnetization transfer can be observed throughout the whole spin system when long mixing times (τ_mix_ ∼ 100 ms) are employed (see red colored protons in second panel from the top in Figure 10). Consequently, the patterns of correlations observed in these spectra are not only useful for spin system assignments, but also to assess the relative stereochemistry of the ring carbons. However, in monosaccharides with *galacto*- and *manno*-configuration, the magnetization is not easily transferred from H1 to the most distant proton atoms due to the small magnitude of ^3^*J*_H4,H5_ and ^3^*J*_H1,H2_, respectively (Figure 7). In pyranose residues with *manno*-configuration, the whole spin system can be traced in the ^1^H,^1^H-TOCSY spectra using the H2 resonance as a starting point for the assignments, which usually present a distinctive downfield chemical shift when compared to other ring protons. In spin systems with the *galacto*-configuration, the assignments of the H5 and H6 resonances can be achieved using ^1^H,^1^H-NOESY spectra, which correlate spins that are close in space such as H4–H5, H4–H6, and/or H3–H5; furthermore, aldohexopyranose residues with the β-anomeric configuration can display correlations between H1 and H5 (such as in the case of the β-d-Fuc*p*3NAc residue of the O-antigen PS from *E. coli* O187 shown in Figure 1,^7^ and the β-d-Gal*p*A and β-d-Gal*p*NAc residues of the O-antigen PS from *E. coli* O155,^194^ as shown in Figure S4 in the Supporting Information of the original article). The proton resonances of the methyl groups of 6-deoxy-sugars are equally as useful as the anomeric resonances as starting points for the assignments because they are found in a characteristic region of the spectrum that is not overlapping with the ring protons. Likewise, the characteristic resonances from the axial and equatorial H3 protons of 3-deoxy-2-ulosonic acids can be used for the same purpose, as well as the H2 protons of 2-deoxyaldoses. In addition, ^1^H,^1^H-TOCSY correlations from amido protons can be employed for the assignment of proton spin systems of *N*-acetylated aminosugars dissolved in H_2_O:D_2_O 98:2 solution, using a water suppression scheme.^158,175^

Once the ^1^H signals have been assigned, correlations to the resonances of their directly attached ^13^C atoms can be achieved using ^1^H,^13^C-HSQC spectra. The multiplicity-edited version of this experiment is useful to discriminate resonances from hydroxymethyl groups because the carbons attached to an even and odd number of protons are phased with opposite sign (see red and blue colored proton–carbon pairs, respectively, in the third panel from the top in Figure 10). Alternatively, when a better resolution is needed in the ^13^C dimension, the ^13^C,^1^H-HETCOR experiment can be used instead but with the inherently lower sensitivity associated with a ^13^C detected experiment.^140,202^ In cases where severe overlap is observed in the ^1^H NMR spectrum, heteronuclear experiments such as ^1^H,^13^C-HSQC-TOCSY can facilitate the assignments of the individual spin systems due to the higher dispersion of chemical shifts in the carbon dimension. In addition, the ^1^H,^13^C-H2BC experiment (vide infra) can be used to correlate proton and carbon spins separated by two covalent bonds.^203^ Furthermore, the ^1^H,^13^C-HMBC experiment can give additional information on proton and carbon spins separated by two or three covalent bonds. The correlations observed in the latter experiment are based on heteronuclear ^2^*J*_CH_ and ^3^*J*_CH_ coupling constants, revealing complementary information to that of the aforementioned experiments. It is worth pointing out that this experiment also plays an important role in the assignment of ^13^C chemical shifts of nonprotonated carbon atoms such as anomeric carbon atoms of ketoses, carbonyl signals in uronic acids, and non-2-ulosonic acids, and quaternary carbons in branched monosaccharides.^76,79^ Moreover, the magnitude and the sign of the ^2^*J*_CH_ couplings within each spin system can also give insights into the differentiation of aldohexopyranose residues (Figure 8), and this information can be retrieved from ^1^H,^1^H-HETLOC, ^1^H,^13^C-HSQC-HECADE, and/or spin-edited ^1^H,^13^C-HSQC-TOCSY experiments.^170,202,204,205^

Additionally, nitrogen bearing carbons can be identified by their characteristic ^13^C chemical shifts (∼ 50–60 ppm). Residues in pyranose and furanose form can usually be differentiated because of the characteristic chemical shifts of their anomeric carbon resonances. In addition, the C4 resonances of aldofuranose residues are found at distinctive downfield ^13^C chemical shifts (∼ 78–86 ppm). In the ^1^H,^1^H-NOESY spectra of aldohexopyranoses, correlations can be observed between axially oriented H1 and H5 protons, whereas in aldohexofuranoses, correlations from H1 to H4 protons located on the same face of the ring may also be detected. Likewise, in the ^1^H,^13^C-HMBC spectrum, correlations between H1–C5 and/or C1–H5 could be observed for aldohexopyranose residues, whereas correlations between H1–C4 and/or C1–H4 are characteristic of aldohexofuranoses. The presence of open-chain monosaccharide residues linked to other moieties via cyclic acetals can be inferred when inter-residue ^1^H,^13^C-HMBC correlations are observed from the anomeric resonances of this monosaccharide to two different positions of the same neighboring monosaccharide residue or aglycone moiety.^109−112^ Alditols such as glycerol, ribitol, erythritol, mannitol, arabinitol, and glucitol can be found as components of teichoic acids;^206^ some of them can also be found as components of lipoteichoic acids, CPS, and repeating units of O-antigen polysaccharides. They can be identified by their lack of anomeric resonances and the presence of two sets of hydroxymethyl groups in their spin system. Even though all of the proton resonances of such residues are usually found in the bulky region of the ^1^H NMR spectrum, ^1^H,^31^P-hetero-TOCSY and/or ^1^H,^31^P-HMBC experiments can assist in the proton chemical shifts assignments because at least one of the hydroxyl groups is involved in a phosphodiester linkage to another residue.^158,207−209^

### 4.4 Anomeric Configuration

**Figure 11:**
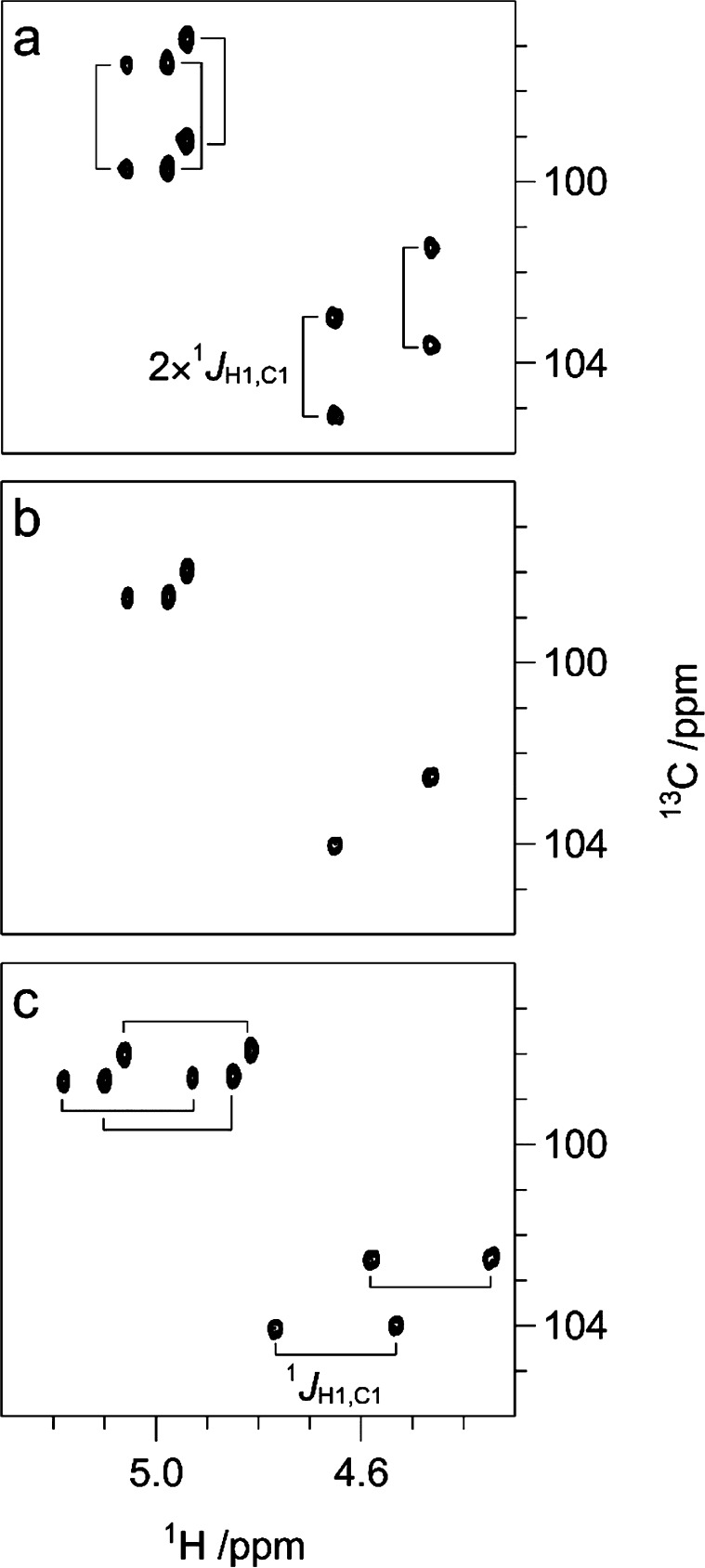
Comparison of the anomeric region of the ^1^H,^13^C-CT-CE-HSQC spectrum (a), ^13^C-decoupled ^1^H,^13^C-HSQC spectrum (b) and coupled ^1^H,^13^C-HSQC spectrum (c) of a polysaccharide of *Vibrio parahemolyticus* AN-16000.^158^

The anomeric configuration of pyranoses that have an H2 proton positioned in an axial arrangement can be deduced from the magnitude of the ^3^*J*_H1,H2_ coupling constants observed in the ^1^H NMR spectrum. As discussed previously, this distinction is difficult to make in polysaccharides where the H2 proton is oriented equatorially because both α- and β-anomeric configurations give rise to small ^3^*J*_H1,H2_ coupling constants (e.g., ∼1.8 and ∼0.8 Hz, respectively, in the case of mannopyranose residues). Consequently, assessing whether a hexopyranose sugar residue has the α- or β-anomeric configuration is often readily performed by determining the magnitude of the ^1^*J*_C1,H1_ coupling constant from an *F*_2_-coupled ^1^H,^13^C-HSQC NMR spectrum (Figure 11c). As a rule of thumb, one has ^1^*J*_C1,H1_ < 168 Hz for an aldohexopyranose residue that has the β-anomeric configuration, whereas ^1^*J*_C1,H1_ > 168 Hz is observed for an α-anomeric configuration. Alternatively, these couplings constants can also be measured using an *F*_1_-coupled ^1^H,^13^C-CT-CE-HSQC NMR spectrum (Figure 11a), in which a scaling factor is used in the experiment to favor the accuracy of the measurement.^158,210,211^

**Figure 12:**
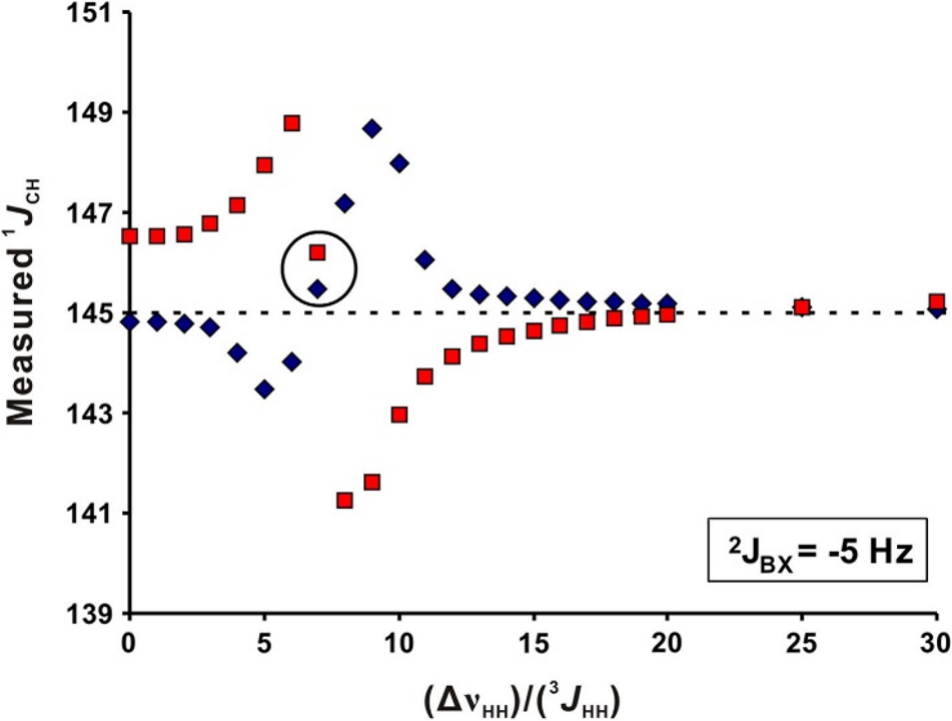
Detailed analysis of strong coupling effects on ^1^*J*_CH_ values measured in the ^1^H dimension (blue) and ^13^C dimension (red) compared to the value predicted from theory. An A^H^B^H^X^C^ spin system is used to simulate 2D coupled ^1^H,^13^C-HSQC spectra. The measured ^1^*J*_CH_ values are plotted against (Δν/^3^*J*_HH_). ^1^*J*_AX_ = 145 Hz, ^3^*J*_AB_ = 10 Hz, and ^2^*J*_BX_ = −5 Hz; a dashed black line is drawn at 145 Hz, the ^1^*J*_CH_ value used in simulation. A significant discrepancy between ^1^*J*_CH_ values measured in the ^1^H and ^13^C is found when 3 ≤ (Δν/^3^*J*_HH_) ≤ 12. Reproduced with permission from ref (164). Copyright 2011 Elsevier.

Heteronuclear carbon–proton one-bond coupling constants in saccharides can also be determined with high resolution from pure absorptive clean in-phase *F*_2_-coupled CLIP-HSQC spectra.^212,213^ However, the apparent ^1^*J*_CH_ value measured from a ^1^H,^13^C-HSQC spectrum can deviate by as much as 7 Hz (Figure 12) and either under- or overestimation of the true coupling constant may occur regardless of whether it is measured in the ^1^H or ^13^C dimension.^164^ The peak separation corresponding to ^1^*J*_CH_ for a proton H_A_ is severely affected when the upfield satellite H_A_^α^(^13^C) significantly overlaps with the resonance from a scalar coupled proton H_B_(^12^C), resulting in a strongly coupled system. Because the ^1^H NMR chemical shifts of anomeric (H1) and vicinal (H2) protons coupled by ^3^*J*_H1,H2_ most often differ quite a bit the strong coupling artifact is seldom a problem. Nevertheless, for some sugar residues such as β-d-Man*p*NAc, one may need to take caution to avoid misinterpretation of data if the chemical shift difference results in spectral overlap and strong coupling as seen from the following example for β-d-Man*p*NAc-OMe, the ^1^H NMR chemical shifts of which were predicted by the CASPER program,^152^ resulting in δ_H1_ ∼ 4.7 and δ_H2_ ∼ 4.5, i.e., Δδ_H_ = 0.2. Assuming a ^1^*J*_C1,H1_ of 160 Hz and a ^1^H spectrometer frequency of 400 MHz, this would result in spectral overlap and strong coupling between H1^α^(^13^C) and H2(^12^C) resonances with potential deviation of ^1^*J*_C1,H1_ from its true value, emphasizing the fact that the anomeric configuration may need to be further supported by information from additional NMR experiments. The presence of strongly coupled spin systems can also affect the outcome of other types of NMR experiments, resulting in artifacts in spectra (cf. section 5.1.7 on pure shift experiments).

The α- and β-anomeric configuration of aldohexopyranosyl residues can also be inferred from the sign of their ^2^*J*_C2,H1_ coupling constants, which can be determined using either ^1^H,^13^C-HETLOC, ^1^H,^13^C-HSQC-HECADE, or spin-edited ^1^H,^13^C-HSQC-TOCSY experiments.^170,204,205^ As illustrated by Oikawa et al., residues with the β-anomeric configuration are expected to display ^2^*J*_C2,H1_ > 0 Hz, whereas those with the α-anomeric configuration have ^2^*J*_C2,H1_ < 0 Hz.^170^ Furthermore, the α- and β-anomeric configuration in residues with the *manno*-configuration can also be deduced from the magnitude of these coupling constants (−1.5 and +8.0 Hz, respectively), as depicted in Figure 8. In the case of Neu5Ac derivatives, the anomeric configuration of the C2 can be determined from heteronuclear geminal and vicinal coupling constants involving the axially oriented proton at position 3 (H3ax); thus, residues with the α-anomeric configuration are characterized by large ^2^*J*_C2,H3ax_ ≈ −8 Hz and ^3^*J*_C1,H3ax_ ≈ 7 Hz, whereas those with a β-anomeric configuration display medium ^2^*J*_C2,H3ax_ ≈ – 4 Hz and small ^3^*J*_C1,H3ax_ ≈ 1 Hz.^214,215^

Analysis of chemical shifts of key proton and carbon resonances can also provide information about the stereochemistry of the anomeric carbon. In aldohexopyranoses with the α-anomeric configuration the resonances of the C5 atoms are found ∼4–7 ppm upfield when compared to their respective counterparts with the β-anomeric configuration (Figure 6). The characteristic chemical shifts differences of the H3ax and H3eq protons of non-2-ulosonic acids can also be used to assess the stereochemical configuration of the anomeric C2 carbon; thus, a large Δδ_H_ value of ∼ 0.7–1.0 ppm is indicative of the C1 carboxylic group oriented axially, whereas a small Δδ_H_ value of ∼ 0.0–0.4 ppm usually implies that this moiety is oriented equatorially. Because the configuration of the anomeric C2 carbon in non-2-ulosonic acids is defined, using as reference the configuration of the C7 atom,^216^ a larger Δδ_H_ between H3ax and H3eq protons is observed in the case of Neu5Ac, Leg, 4eLeg, and 8eLeg residues with the α-anomeric configuration, and Aci, 8eAci, Fus, Pse, and 8ePse residues with the β-anomeric configuration, when compared to their respective C2 epimers.^75,82−84,140,183,217^ However, it is worth pointing out that approaches involving chemical shifts comparisons and ^3^*J*_C1,H3ax_ may fail in some cases because these values can be affected by the aglycone substituents, particularly in synthetic derivatives.^218^

Moreover, experiments based on the nuclear Overhauser effect (such as ^1^H,^1^H-NOESY or ^1^H,^13^C-HSQC-NOESY) may also reveal information related to the configuration of the anomeric carbon, using key through-space correlations from the anomeric proton to nonvicinal intraresidue ring protons. Thus, all of the aldohexopyranoses with the β-anomeric configuration depicted in Figure 3 have the potential to show direct through-space correlations between H1 and H5, whereas only those with the *gluco*-, *manno*-, *galacto*-, and *talo*-configuration could show correlations from the anomeric proton to the axially oriented H3 atom.

### 4.5 Linkage Positions and Sequential Arrangement between Sugar Residues

Linkage positions in oligo- and polysaccharides may be identified by large ^13^C NMR glycosylation shifts, i.e., the difference in chemical shift of the substituted position when compared to that of the corresponding monosaccharide, being on the order of Δδ_C_ ∼ 4–11 ppm.^156,197^ Because the resonances of carbon atoms adjacent to glycosylated positions can be shifted upfield by ∼ 2 ppm when compared to the corresponding monosaccharides, the combined effect of a double substitution at neighboring positions of the same monosaccharide residue may result in glycosylation shifts smaller than anticipated, as observed for the C4 carbon resonance of the →3,4)-α-d-Gal*p*NAc-(1→ residue of the O-antigen PS of *E. coli* O181 (Δδ_C4_ ∼ 2.6), and the C3 resonances of the →3,4)-α-l-Fuc*p*NAc-(1→ and →3,4)-β-d-Glc*p*NAc-(1→ residues of the PS of *V. parahemolyticus* AN-16000 (Δδ_C3_ ∼ 2.2) and the O-antigen PS of *E. coli* O115 (Δδ_C3_ ∼ 2.8), respectively.^134,158,175^ Furthermore, when monosaccharide residues are linked to other moieties via a phosphodiester bonds, the linkage position can also be inferred from the splitting of proton and/or carbon resonances due to ^3^*J*_HP_ and ^2^*J*_CP_ coupling constants.

An alternative approach to distinguish linkage positions relies on the observation of hydroxyl protons in oligosaccharides in supercooled aqueous solutions, at −14 °C, and to use these as starting points for resonance assignments and structural determination. These protons resonate in the spectral region 5.5–8.5 ppm and the presence and absence of proton–proton correlations in ^1^H,^1^H-COSY or ^1^H,^1^H-TOCSY spectra with a long mixing time of 140 ms facilitates the determination of linkage positions.^219^ The hydroxyl-based approach was recently extended by using also ^13^C NMR correlations from ^1^H,^13^C-HSQC experiments resulting in regular one-bond correlations to nonexchangeable protons and ^1^H,^13^C-HSQC-TOCSY experiments, where the latter first used a short mixing time of only 8 ms to obtain correlations between hydroxyl groups and adjacent ^13^C nuclei, which taken together form constituent pieces, H–C–O–H, of a jigsaw puzzle.^149^ A series of longer isotropic mixing times of up to 90 ms enables both resonance assignments and identification of linkage positions, due to lack of correlations in the ^1^H,^13^C-HSQC-TOCSY spectrum when the short mixing time is used. Interestingly, to improve the signal-to-noise ratio three capillary NMR tubes were packed inside a 3 mm NMR tube. It is noteworthy that the ^1^H,^13^C–H2BC experiment offers an alternative for detection of correlations between hydroxyl groups and adjacent ^13^C nuclei, as demonstrated for the O-antigen PS of *E. coli* O142, where the OH signals readily could be assigned at 2 °C in a H_2_O/D_2_O 95:5 solution.^148^

The sequence of monosaccharide residues in glycans, and their linkage positions, can be established using proton–proton through-space inter-residue correlations from ^1^H,^1^H-NOESY, ^1^H,^1^H-ROESY, ^1^H,^13^C-HSQC-NOESY and/or ^1^H,^13^C-HSQC-ROESY experiments, as well as three-bond inter-residue proton–carbon correlations from ^1^H,^13^C-HMBC experiments (Figure 10 bottom). The outcome of these experiments is strongly influenced by the torsion angles preferences around the glycosidic linkage and, in the case of the ^1^H,^13^C-HMBC experiment, the intensity of the observed cross-peaks will depend on both the magnitude of the corresponding inter-residue ^3^*J*_CH_ coupling constant and the selected long-range coupling evolution delay (Δ) of the experiment, with the maximum intensity observed when Δ = 1/(2·^3^*J*_CH_).^220^ In this regard, loss of magnetization due to fast *T*_2_ relaxation may limit signal-to-noise performance of small ^3^*J*_CH_-based correlations in the ^1^H,^13^C-HMBC spectrum of large polysaccharides. In ketose residues, the sequence analysis is restricted to the detection of ^1^H,^13^C-HMBC correlations from the anomeric carbons to the proton(s) located at the substitution positions. When different anomeric carbon resonances of a glycan fall close together in the ^13^C NMR spectrum, a ^1^H,^13^C-HMBC spectrum with improved resolution in the indirect dimension may be required to unambiguously assign the corresponding cross-peaks; thus, a band-selective constant-time version of this experiment can be employed (cf. selective excitation experiments in section 5.1.2).^221^ Experiments based on the nuclear Overhauser effect may be useful to establish through space correlations between an anomeric proton and a hydrogen atom at the substitution position; however, because the dipolar interaction depends on the proximity of the proton spins, additional cross-peaks could be observed to close in space neighboring positions and may lead to misinterpretation of the data. For instance, in the trisaccharide moiety, α-d-Gal*p*NAc-(1→2)-β-d-Qui*p*3NAc-(1→3)-β-d-Rib*f*, present in the O-antigen PS of *E. coli* O5ac, three different inter-residue ^1^H,^1^H-NOESY correlations were observed from the anomeric proton of the β-d-Qui*p*3NAc residue to the H2, H3, and H4 atoms of the Rib*f* residue.^222^ In addition, two trough-space correlations from the anomeric proton of the Gal*p*NAc residue are observed to H1 and H2 of the directly attached Qui*p*3NAc residue, and an additional correlation is observed to H4 of the nondirectly linked β-d-Rib*f* residue. It is worth pointing out that to avoid misinterpretation of the data due to spin-diffusion artifacts, the ^1^H,^1^H-NOESY spectra employed for the analysis of large polysaccharides should be recorded using short mixing times of ∼ 50 ms and can be complemented by the ^1^H,^13^C-DDCCR experiment to correlate inter-residue proton–carbon pairs based on cross-correlated dipolar relaxation (cf. section 5.2.2).^223,224^ In oligo- and polysaccharides, where a monosaccharide residue is connected via a phosphodiester group to another monosaccharide residue or alditol, ^1^H,^31^P-hetero-TOCSY and/or ^1^H,^31^P-HMBC experiments can be useful to establish the sequence of the disaccharide moiety.^105,175^

Determination of the biological repeating unit of a polysaccharide can be achieved by NMR spectroscopy if the low intensity signals corresponding to the terminal nonreducing end of the polysaccharide can be identified, particularly in the anomeric proton region. These signals are sometimes sharper than those of the internal monosaccharides due to the higher flexibility of the polysaccharide terminus, facilitating the spin system assignments. Comparison of these resonances’ intensities with those of the respective internal residues can be used to estimate the degree of polymerization of the PS.^136,194,225^ Additionally, the average molecular weight of neutral polysaccharides can be estimated from their translational self-diffusion coefficients, measured using ^1^H NMR diffusion experiments and the approximation developed by Viel et al.^226,227^

### 4.6 Substituents Appended to Sugars

The diversity of glycans found in nature is increased by different structural alterations of the basic monosaccharide structures.^228^ In polysaccharide repeating units, these modifications can be homogeneously or heterogeneously distributed throughout the polymer or confined to the terminal end of the polysaccharide. Modification by hydroxyl groups through *O*-acetylation is quite common in glycans and, because acetyl migration^229^ or partial hydrolysis may take place in the natural environment in which these biomolecules are found, or during extraction and purification procedures, heterogeneous structures containing nonstoichiometric amounts of *O*-acetyl groups can be produced. For instance, different populations of *O*-acetylated rhamnose and galacturonic acid residues, six and two respectively, could be identified in the O-antigen PS of *E. coli* O115 (viz. Rha*p*2Ac, Rha*p*3Ac, Rha*p*4Ac, Rha*p*2Ac3Ac, Rha*p*2Ac4Ac, Rha*p*3Ac4Ac, Gal*p*A2Ac, and Gal*p*A3Ac) besides the corresponding non-*O*-acetylated moieties.^134^ In sialic acid derivatives, *O*-acetylation of the hydroxyl groups may typically be found at the O4, O7, O8, and/or O9 positions,^230^ and the presence of these substituents can be inferred by the observation of methyl proton resonances in the spectral region between ∼2.1–2.2 ppm. Additionally, other substituents such as lactic, succinic, and long-chain aliphatic acids can be linked via ester bonds to hydroxyl groups of monosaccharide residues. Sucrose esters isolated from fruits of different plant genera, and monosaccharide fatty acid esters obtained through enzymatic reactions are currently being explored as potential surfactants and antibacterial reagents in food industry applications.^231−233^ In all of these cases, the chemical shifts of the hydrogen atoms located at the *O*-acetylated position are shifted downfield by Δδ_H_ ∼ 0.5–1.7 when compared to the respective nonsubstituted monosaccharides and, in some cases, may show up in the same spectral region where the anomeric proton resonances reside; in the case of the O-antigen PS of *E. coli* O115, the H2 resonances of Rha*p*2Ac, Rha*p*2Ac3Ac, and Rha*p*2Ac4Ac are found at δ_H2_ 5.49, 5.61, and 5.54, respectively, whereas H3 of Gal*p*A3Ac is found at δ_H3_ ∼ 5.26.^134^ The location of these substituents can be determined through heteronuclear three-bond ^1^H,^13^C-HMBC correlations from the carbonyl resonances of the substituent to the proton spins located at the respective substitution positions. Because the carbonyl resonances appear in a narrow region of the ^13^C NMR spectrum (δ_C_ ∼ 170–180), a band-selective constant-time version of this experiment can be used to improve *F*_1_ resolution (see section 5.1.2).^134,175,187,234^

Pyruvic acid can be linked to monosaccharide constituents of polysaccharides and glycoconjugates via ether or cyclic acetal linkages. In the latter case, six-membered rings are formed when the O4 and O6 atoms of aldohexopyranose residues are involved in the linkage (such as in the case of the secondary cell wall polymer of *P. alvei* CCM 2051 illustrated in Figure 5a),^106^ whereas five-membered rings are characteristic of 2,3- or 3,4-*O*-linked ketal pyruvates. The occurrence of these entities can be inferred when resonances of methyl groups are present at δ_H_ ∼ 1.3–1.7 and δ_C_ ∼ 17–30, and quaternary ketal carbon resonances (C2) are observed at δ_C2_ ∼ 100 (six-membered rings) or ∼110 ppm (five-membered rings). Furthermore, the linkage positions in the monosaccharide residue can be identified by analysis of glycosylation shifts, and the presence of three-bond ^1^H,^13^C-HMBC correlations from the ketal carbon of the pyruvic acid moiety to the hydrogen atoms at the substitution positions. Additionally, through space ^1^H,^1^H-NOESY correlations from the methyl proton resonances can be used to retrieve this information and the stereochemistry of the C2 carbon atom. In the case of 4,6-*O*-linked pyruvates, analysis of ^1^H and ^13^C chemical shifts of the methyl group resonances can also give insights into the stereochemistry of the ketal carbon (i.e., typical values of δ_H_ ∼ 1.65–1.68 and δ_C_ ∼ 17 are observed in the case of axially oriented CH_3_ groups, whereas δ_H_ ∼ 1.46–1.52 and δ_C_ ∼ 26 are observed when the methyl group is equatorially orientated).^235^

Substituents attached via ether bonds to hydroxyl groups of glycan structures are less common. For example, *O*-methylation of glycans have only been reported in bacteria, fungi, algae, plants, worms, and mollusks but not in mammals.^236^ In sialic acids derivatives, *O*-methylation has been observed at the O8 and O9 positions, such as in the case of the recently reported Kdn8Me and Neu5Gc8Me residues found in the glycome of the cephalochordate *B. belcheri*,^237^ and the Kdn9Me residue found in the O-antigen PS from *P. sedimentorum* KMM 9023^T^.^238^ The proton and carbon resonances of this substituent are observed at characteristic δ_H_ ∼ 3.4 and δ_C_ ∼ 59.^239^ Besides amide and ester linkages, lactic acid residues can be attached to glycans through ether bonds, and the *N*-acetyl muramic acid residue found in the peptidoglycan is an example of the latter (Figure 5a); in that case, the proton resonances of the lactyl group are observed at δ_H_ ∼ 1.4 (CH_3_) and 4.4 (CH), and the ether linkage can be recognized by the characteristic downfield chemical shift of the C2 resonance of the lactic acid moiety (δ_C2_ ∼ 78 ppm).^152^ Interestingly, the repeating unit of the EPS from *S. thermophilus* contains a glucose residue that is 6-*O*-substituted with a nononic acid moiety through an ether linkage (Figure 5b).^107,108^ Furthermore, it has recently been demonstrated that hemicellulose chains can be covalently linked to lignin through ether bonds because long-range proton–carbon correlations could be observed in the ^1^H,^13^C-HMBC spectrum of a lignin–hemicellulose complex from the α-proton/α-carbon atoms of the lignin subunits to the C6/H6 nuclei of mannose residues present in a glucomannan polymer.^240^ Besides ^1^H,^13^C-HMBC experiments, ^1^H,^1^H-NOESY, and/or ^1^H,^13^C-HSQC-NOESY correlations can be useful to establish proton–proton correlations at the ether linkage.

Aminosugars can be recognized by the characteristic upfield chemical shifts of their nitrogen-bearing carbons (δ_C_ ∼ 50–60). The presence of methyl group resonances at δ_H_ ∼ 2.0 ppm and δ_C_ ∼ 23 ppm may indicate that the amino groups are *N*-acetylated, a modification that is widely distributed in nature. Amide linkages with carboxylic acid containing structures such as glyceric (GroA, see Figure 5d), glycolic (Gc), 3-hydroxybutyric (3Hb), 4-hydroxybutyric (4Hb), and succinic acid (Suc) can also be found in nature, in addition to diverse aliphatic acids and amino acids moieties (see *N*-acetyl aspartic acid moiety in Figure 5e).^114^ In an analogous manner to what was described for ester linked substituents, the location of these substituents can be determined through heteronuclear three-bond ^1^H,^13^C-HMBC correlations from the carbonyl resonances of the *N*-acyl groups to the protons at the substitution positions. Furthermore, the amide protons can be observed in H_2_O:D_2_O solution at δ_H_ ∼ 8 ppm and assist in the assignment of proton and carbon resonances of the directly attached moieties (i.e., monosaccharide and substituent) by employing homo- and heteronuclear 2D experiments. Lactyl groups linked via amide groups are rare, but some examples were recently reported in polysaccharides produced by bacteria of the *T. fructosivorans*, *S. litorea*, and *P. marincola* species, where they are respectively linked to Rha4N, Fuc3N, and Pse residues.^241−243^ In contrast to what is observed for ether-linked lactyl groups, the C2 resonance of *N*-lactyl moieties is found upfield at δ_C2_ ∼ 70. Furthermore, glyceric and 4-hydroxybutyric acid structures, connected via amide bonds to Qui4N (Figure 5d) and Pse residues, respectively, have been found as components of the backbone of some bacterial polysaccharides.^113,244^ The presence of *N*-acetimidoyl groups in *E. coli* O-antigen PS is restricted to only three serogroups (viz. O118, O145, and O151),^26^ and the corresponding methyl resonances are observed at δ_H_ ∼ 2.2 and δ_C_ ∼ 20.^245^ In some glycans, Pse, Leg, and 8eLeg residues can display this type of substituents at the N5 position and, in the particular case of the LPS of the wild-type strain RC1 of *L. pneumophila*, three further *N*-methylated derivatives of this substituent were reported: 5-*N*-(*N*,*N*-dimethylacetimidoyl), 5-*N*-(*N*-methylacetimidoyl), and 5-*N*-acetimidoyl-5-*N*-methyl.^246^*N*-Formyl groups (Fo) display a characteristic resonance in the ^1^H NMR spectrum at δ_H_ ∼ 8.0, and they can also be found as substituents of Pse residues.^121^ In addition, amino groups can be *N*-sulfated, such as in the case of heparan sulfate.^247^ Nitrogen–proton correlations from ^1^H,^15^N-HSQC and ^1^H,^15^N-HSQC-TOCSY spectra recorded using a H_2_O:D_2_O 98:2 solution, or long-range ^1^H,^15^N-HMBC or ^1^H,^15^N-HNMBC spectra recorded using D_2_O, can be employed to establish the substitution positions of amino, *N*-acyl, and *N*-sulfated derivatives; in the IMPACT version of the latter two experiments, constant-time (see description of constant-time ^1^H,^13^C-HMBC experiment in section 5.1.2) and ASAP features (see fast NMR experiments in section 5.1.5) have been implemented in order to offer improved sensitivity as well as enhanced resolution in the *F*_1_ dimension.^159,160^ In these spectra, the ^15^N chemical shifts of amido and *N*-sulfated moieties can be found at δ_N_ ∼ 115–125 and ∼93 ppm, respectively, whereas unsubstituted amino groups can be observed at δ_N_ ∼ 30.^141,146,157,159,160^

Hydroxyl groups of monosaccharide residues can undergo phosphorylation, or form phosphodiester bonds with other phosphorylated monosaccharides or substituents such as alditol phosphates, phosphoethanolamine (Figure 5e), phosphocholine, glycerol-1-phosphate (Figure 5d), glycerol-2-phosphate, 3- and 2-phosphoglyceric acid, among others.^248^ The number of phosphorus-containing moieties present in a glycan structure can readily be identified using a 1D ^31^P NMR spectrum; thus, phosphomonoesters (singlet at δ_P_ ≈ 10 to 0), phosphodiesters (singlet at δ_P_ ≈ 0 to −5), diphosphomonoesters (doublets at δ_P1_ ≈ −5 and δ_P2_ ≈ −10) and diphosphodiesters moieties (doublets at δ_P_ ≈ −10) can be differentiated because of their characteristic ^31^P chemical shifts and the presence/absence of a ^2^*J*_PP_ coupling constant ∼21 Hz.^249,250^ Even though 1D ^31^P-decoupled ^1^H experiments can be employed to reveal the proton resonances at the substitution positions,^106,248,251^ 2D experiments such as ^1^H,^31^P-HMBC^105,252^ and ^1^H,^31^P-hetero-TOCSY are more suitable when the target proton resonances are found in the bulk region of the ^1^H NMR spectrum or when multiple phosphate groups are present in the glycan. The latter experiment is also useful for assignment of proton spin systems connected to the phosphorus-containing group; in this case, magnetization transfer can be promoted from the neighboring to the more distant protons using mixing times of increased duration.^158,175,207,208,248^ Considering the relatively good sensitivity of ^31^P nuclei, heteronuclear detected versions of the aforementioned 2D experiments can be implemented when better resolution of the phosphorus resonances is required. It is noteworthy that some phosphate groups can characteristically be found at terminal ends of different polysaccharides, such as in the case of the methylated phosphate group linked to the anomeric carbon of the reducing end monosaccharide residue of *Leptospira* lipid A.^251^ Analogously, the O3 position of the mannose residue located at the nonreducing terminus the O-specific chains of the LPS from *K. pneumoniae* O3, *H. alvei* PCM 1223, and *E. coli* O9 is capped with a methyl phosphate group, which acts as a signal for termination of the chain elongation.^253^ In both cases, the methyl resonances are found at the characteristic chemical shifts of δ_H_ ∼ 3.6 (d, ^3^*J*_HP_ = 11 Hz) and δ_C_ ∼ 54. In addition, pyrophosphate moieties can be found in the lipid A-core region of some LPS of gram-negative bacteria,^249^ whereas in gram-positive bacteria, both phosphodiester and diphosphodiester linkages (Figure 5a) can be involved in the covalent attachment of cell wall polysaccharides to the peptidoglycan.^105,106^

Lipoarabinomannans from *M. tuberculosis* and *M. kansasii* are capped with an unusual 5-deoxy-5-methylthio-d-xylofuranose residue and its corresponding oxidized sulfoxide derivative.^72,73^ The resonances of the methyl groups in these moieties are found at δ_H_/δ_C_ ∼ 2.1/ ∼ 17 for SCH_3_ and δ_H_/δ_C_ ∼ 2.8/∼ 40 for S(O)CH_3_, whereas the ^13^C chemical shifts of the C5 resonances of the xylofuranose residue were found at δ_C_ ∼ 34 and ∼ 56 for the thioether and sulfoxide derivatives, respectively. When unaccounted ^1^H and ^13^C chemical shift displacements are observed at specific positions of monosaccharide residues and key resonances and correlations from other substituents are absent in the ^1^H, ^13^C, ^15^N, and ^31^P based experiments, the presence of *O*-sulfation can be suspected.^254^ This modification can typically be found at O8 and O9 positions of some Neu5Ac derivatives and is heterogeneously distributed over different Fuc and GalNAc residues in the fucosylated chondroitin sulfate isolated form the sea cucumber *P. pygmaea* and Gal residues in the sulfated galactan obtained from the red alga *B. occidentalis*.^255,256^ Residues displaying this type of modification have also been found in several O-antigen polysaccharides isolated from marine bacteria, including α-d-Glc*p*A2S3Ac in the PS from *P. sedimentorum* KMM 9023^T^, β-d-Qui*p*2S3N(4Hb) in the PS of *I. abyssalis* KMM 227^T^, α-d-Glc*p*2Ac3S and β-d-Gal*p*3S residues in the PS of *C. pacifica* KMM 3879^T^, and β-d-Gal*p*2S3S residue in the PS of *C. pacifica* KMM 3878.^238,257−259^

## 5 NMR Experiments for Resonance and Sequence Assignments

### 5.1 General Considerations

2D NMR spectroscopy techniques were developed during the 1980s, followed by 3D and nD experiments, which were advanced during the 1990s, in particular, with ^13^C and/or ^15^N isotope labeling of proteins and nucleic acids, but also for carbohydrates.^260−262^ On-cell NMR spectroscopy by high-resolution magic angle spinning was described for bacterial polysaccharides,^263^ 1D ^1^H pure-shift NMR spectroscopy was developed and applied to sucrose,^264^ and Hadamard NMR spectroscopy was put forward as an efficient technique during 1990s.^265^ With these developments in hand, new NMR methods and applications emerged and have become established during the last two decades, described below for oligo- and polysaccharides as well as for glycopeptides and glycoproteins.

#### 5.1.1 H2BC NMR Experiments

**Figure 13:**
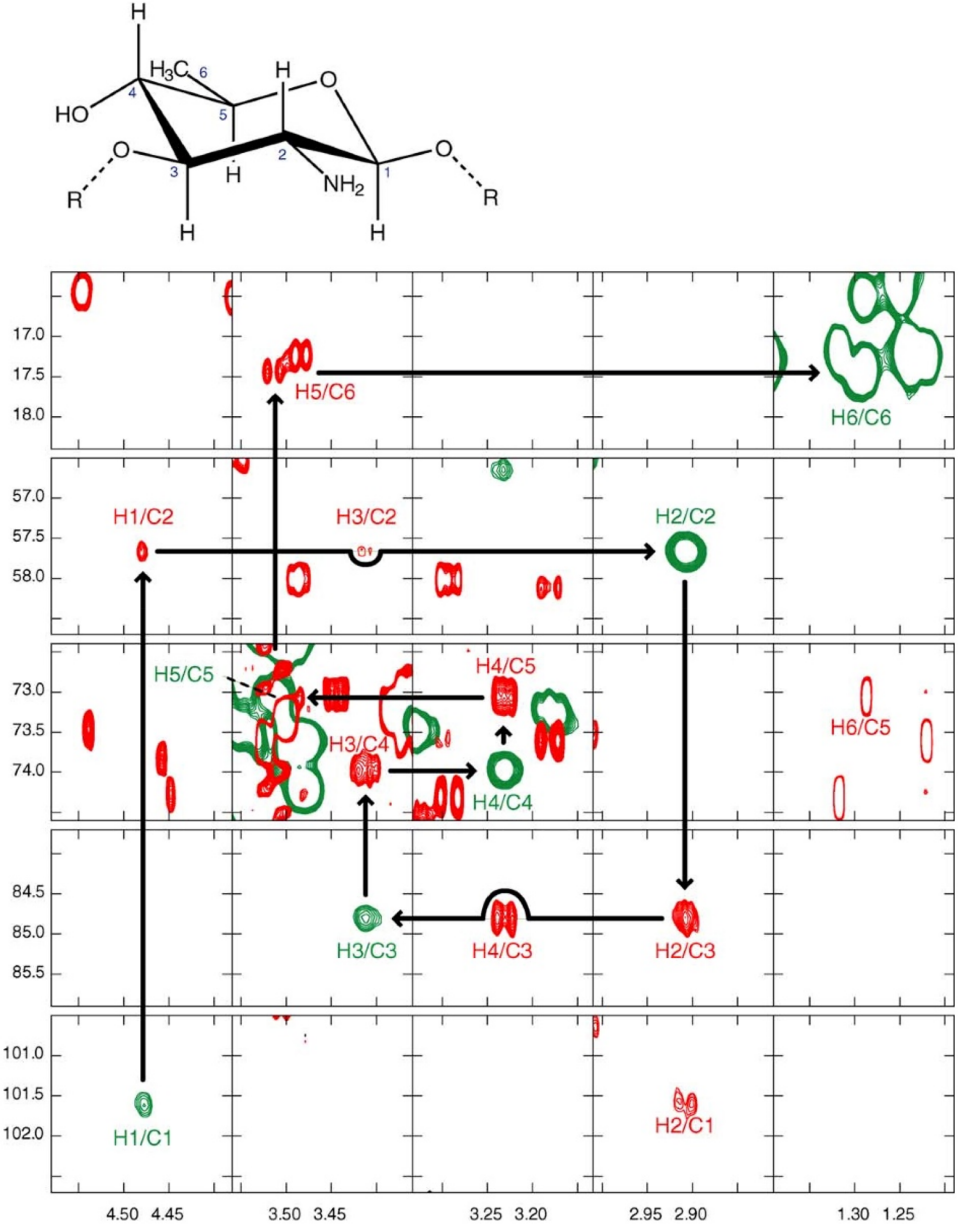
Illustration of a ^1^H,^13^C-heteronuclear “sequential walk” in an H2BC spectrum for the assignment process of the complete spin system for a 3-substituted β-d-QuiN residue in an oligosaccharide from the LPS of *Francisella victoria* based on the overlaid HSQC (green) and H2BC (red) spectra starting from the anomeric H1/C1 cross-peak in the HSQC spectrum via correlations in the H2BC spectrum to H1/C2, but also to C1/H2, all the way to the H6/C6 cross-peak of the methyl group. Reproduced with permission from ref (267). Copyright 2011 Elsevier.

The classical ^1^H,^13^C-HMBC NMR experiment shows correlations over two and three bonds based on heteronuclear scalar coupling between the nuclei, which gives essential information in assigning NMR resonances in carbohydrates. However, because one a priori does not know how far, i.e., over how many bonds, these correlations occur, the interpretation of the data is still requiring additional information. A solution to this problem could be to carry out a complementary 2D ^1^H,^13^C-HMQC-COSY or a ^1^H,^13^C-HSQC-TOCSY experiment with a very short mixing time (τ_mix_ 10 ms), but the spectra may still be quite crowded when oligosaccharides or larger structures are to be analyzed. A remedy to this was proposed based on the HMQC-COSY combination and is referred to as a ^1^H,^13^C-H2BC NMR experiment relying on ^1^*J*_CH_ and ^n^*J*_HH_ couplings for coherence transfer.^266^ The experiment, which is of constant-time type, suppresses both homo- and heteronuclear couplings in the indirect dimension and heteronuclear decoupling is carried out during the acquisition. Notably, the H2BC spectra show only cross-peaks involving ^13^C spins *j*, for which there is a nonvanishing ^n^*J*_HH_ coupling between spins H_*j*_ and H_*k*_, where H_*k*_ is a vicinal or geminal proton. In the analysis of intraring correlations in sugar residues, one typically observes two correlations for each atom position *n* in the sugar residue along the *F*_2_ dimension corresponding to H(*n* – 1)/C*n* and H(*n* + 1)/C*n* and also two correlations along the *F*_1_ dimension corresponding to C(*n* – 1)/H*n* and C(*n* + 1)/H*n*. This information then facilitates a ^1^H,^13^C-heteronuclear “sequential walk” in the H2BC spectrum (Figure 13),^267^ unraveling in many cases the complete spin system of a monosaccharide entity in a glycan molecule. The limitation of H2BC technique is similar to that of ^1^H,^1^H-TOCSY experiments in that small coupling constants, such as ^3^*J*_H4,H5_ in pyranose sugars having the *galacto*-configuration, may hamper magnetization transfer. Further developments and applications of the H2BC experiment have been reported,^268,269^ one of which was a 3D H2BC NMR experiment.^203,270^

Combining the ^1^H,^13^C-H2BC NMR experiment with one-bond correlations from a ^1^H,^13^C-HSQC-type of experiment would obviate the separate acquisition of the latter and the 2BOB (two-bond one-bond) experiment with editing possibilities has been proposed to this end.^271^ If the experiment is performed such that all peaks appear in the spectrum, it is referred to as H2OBC (heteronuclear two-bond one-bond correlations), where the cross-peaks due to one or two bonds can be distinguished based on the π/2 phase difference in the proton dimension (*F*_2_).

#### 5.1.2 Selective Excitations

Selective pulses as part of an NMR experiment can be used to efficiently carry out excitation at a specific resonance frequency, thereby facilitating, e.g., 1D experiments instead of relying on full 2D or higher dimensions in acquisition of NMR data. The selective excitation, inversion or refocusing may employ Gaussian, SNOB or BURP shaped pulses, or other profiles that efficiently pick out a narrow spectral region.^272^ Subsequent application of a ^1^H,^1^H-TOCSY spin-lock sequence may be used to identify carbohydrate components, such as those from polysaccharides of biofilms from *Staphylococcus epidermidis*.^273^ When applying these selective pulses at ^1^H frequencies where anomeric protons reside, multistep correlations can be identified from 2D selective-TOCSY-DQFCOSY and selective-TOCSY-NOESY experiments as shown by Sato et al. for lactose, di-, and triantennary N-glycan oligosaccharides.^274−276^

**Figure 14:**
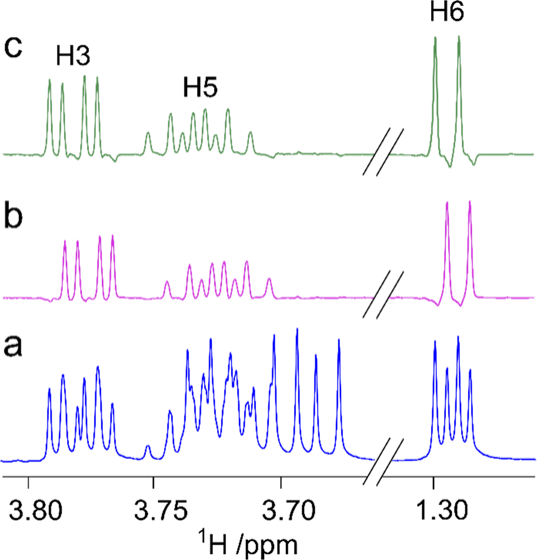
(a) Selected regions of the ^1^H NMR spectrum of rutinose, α-l-Rha*p*-(1→6)-d-Glc*p*. (b) The corresponding regions of the 1D ^1^H,^1^H-CSSF-TOCSY spectrum in which the H6 resonance of rhamnose at 1.290 ppm was targeted. The mixing time used was 80 ms. (c) The corresponding 1D ^1^H,^1^H-CSSF-TOCSY spectrum in which the H6 resonance of rhamnose at 1.295 ppm was targeted. The intensities of the H6 resonances are reduced relative to those from the ring protons. Reproduced with permission from ref (279). Copyright 2011 Elsevier Publisher.

However, if the frequency difference between resonances that are to be targeted is minute, the above-described selective pulses may not be sufficient in resolving peaks that only differ by a few hertz. Provided that the peaks from protons still have distinct chemical shifts, then a chemical shift selective filter (CSSF) may be utilized.^277,278^ In the pseudo 2D experiment, the variable time (VT) chemical shift evolution period is incremented up to a maximum value, which defines the selectivity of the filter, typically set as *t*_max_ = 0.5/Δν, where the chemical shift difference between the overlapping peaks is given by Δν and may be as low as 1.4 Hz.^277^ When the FIDs are added, the on-resonance magnetization is constructively enhanced, while the off-resonance magnetization, which differs in phase, is eliminated by destructive interference. The application of the CSSF technique is shown for the resonance assignments of the disaccharide rutinose in which the methyl group resonances of the terminal rhamnosyl residue differed by only ∼ 3 Hz, and application of a 1D ^1^H,^1^H-VT-CSSF-TOCSY readily resolved the NMR chemical shifts of both its H3 and H5 resonances of the anomeric mixture (Figure 14).^279^ The CSSF methodology has successfully been applied to assign chemical shifts in arabino-containing oligosaccharides using TOCSY experiments,^485^ and to resolve closely resonating methyl groups in an *N*-acetyl-containing trisaccharide using NOESY experiments.^280^

The VT-CSSF NMR experiments are effective but not efficient, and a constant-time method referred to as GEMSTONE (gradient-enhanced multiplet-selective targeted observation NMR experiment)^281^ was proposed to resolve the limitation of performing a pseudo-2D NMR experiment. GEMSTONE uses swept-frequency pulses to produce spatially dependent chemical shift evolution, which in turn leads to destructive interference of the off-resonance signals. The spatial encoding in the different parts of the sample in effect performs the incrementation used in the CSSF experiment and can be seen as a single scan analogue of the CSSF experiment. Subsequent to the GEMSTONE module, NOE^281^ or TOCSY^282^ modules can be added. The latter experiment was applied to the aminoglycoside antibiotic amikacin and the flavone glycosides hesperidin and naringin, containing α-l-Rha*p*-(1→6)-β-d-Glc*p* and α-l-Rha*p*-(1→2)-β-d-Glc*p* disaccharide structural elements.

**Figure 15:**
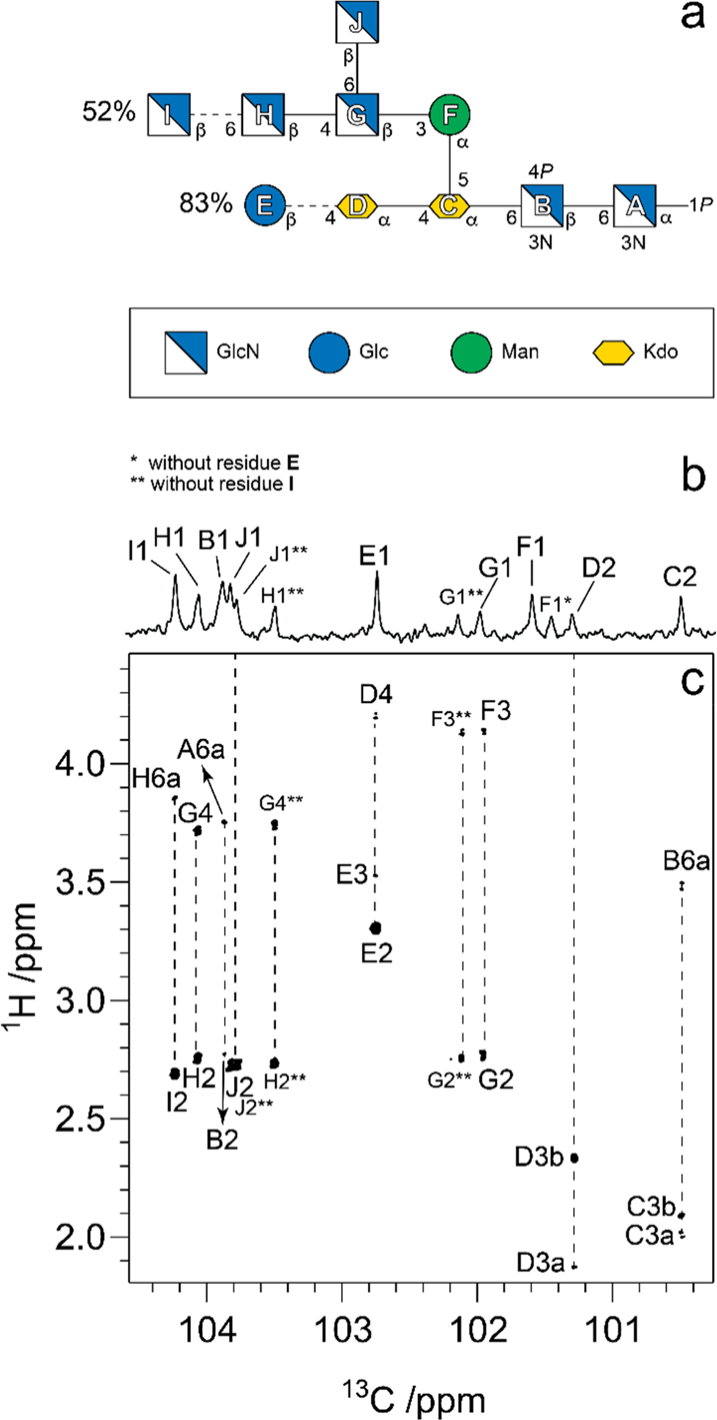
(a) Structure of the deacylated R-LPS of *Brucella melitensis* strain Bm_*wbkD* in SNFG format.^135^ (b) Selected section of the anomeric region of the ^13^C NMR spectrum and (c) band-selective constant-time ^1^H,^13^C-HMBC spectrum recorded over a spectral region of 5.4 ppm × 9.0 ppm with 2048 × 256 data points, using a selective ^13^C excitation pulse applied at the center of the region for anomeric carbons.

The monosaccharide entities of glycans often contain a carbonyl group as part of a functional group, in particular *N*-acetyl groups, carboxylic acids, amino acids linked via an amide bond to the monosaccharide, *O*-acetyl, and more complex ester groups as substituents. The ^13^C NMR resonances of the different carbonyl groups reside in the quite narrow spectral region of ∼ 170–180 ppm and the ^1^H,^13^C-HMBC experiment can reveal important correlations to unravel structural information. To increase the resolution in the *F*_1_-dimension of a ^1^H,^13^C-HMBC spectrum, Nuzillard and co-workers developed a band-selective experiment, which covered a narrow spectral region of 16 ppm and applied it to an arabinoxylan pentasaccharide.^283^ This facilitated improved spectral interpretation and cross-peak identification thanks to improved resolution in the *F*_1_-dimension. However, by reducing the spectral width, a conspicuous and undesirable skew becomes evident along the *F*_1_-dimension of the ^1^H,^13^C-HMBC spectrum. A band-selective constant-time ^1^H,^13^C-HMBC experiment,^221^ which, e.g., uses a Q3 Gaussian cascade for the selective π-pulse on the ^13^C channel, alleviates the problem because no net modulation by *J*_HH_ coupling evolution takes place, and as a result, the fine structure of the proton–proton couplings do not appear in the *F*_1_-dimension. The ^1^H,^13^C-BS-CT-HMBC experiment has been useful in elucidating carbohydrate-containing natural products and polysaccharides from different bacterial species.^158,284,285^ The concept of band-selective excitation was used to resolve complex NMR spectra of the core region of the LPS from *Brucella melitensis*, where the ^1^H,^13^C-BS-CT-HMBC experiment instead was applied on resonances at the anomeric region of an oligosaccharide mixture (Figure 15).^135^

#### 5.1.3 TILTed NMR Spectra

**Figure 16:**
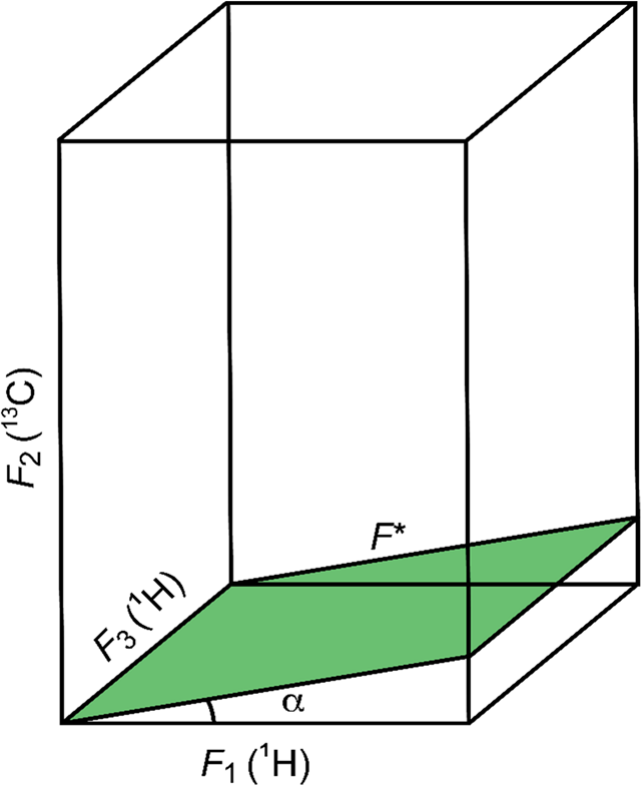
Schematic representation of a 3D ^1^H,^1^H-NOESY-^1^H,^13^C-HSQC NMR spectrum, where the *F*_2_ axis displays the carbon-13 frequencies and the tilted plane F*/*F*_3_ contains contributions from both ^1^H and ^13^C NMR chemical shifts, thereby resolving spectral overlap present in a regular 2D NMR spectrum. Adapted with permission from ref (287). Copyright 2007 Elsevier Publisher.

The limited ^1^H NMR spectral dispersion of glycans pose problems due to degenerate chemical shifts or highly overlapped spectral regions. The crowded spectra make assignment of resonances difficult when using ^1^H,^1^H-TOCSY experiments as well as for obtaining sequence information between sugar residues relying on ^1^H,^1^H-NOESY experiments. A conceptually straightforward remedy will be to record 3D spectra in which the proton resonance overlap can be resolved by distributing the 2D-planes along a third dimension, typically containing resonances from ^13^C nuclei, in, e.g., a 3D ^1^H,^1^H-TOCSY-^1^H,^13^C-HSQC NMR experiment. However, this will increase the experimental time significantly, by up to 2 orders of magnitude (unless nonuniform sampling is employed, vide infra). By still using the ^13^C-dimension, but to a lesser extent, and record a tilted projection where the plane makes an angle α to the *F*_1_/*F*_3_ plane (TOCSY or NOESY), the spectral overlap may be alleviated. The tilted projection is accomplished by linking the evolution increments Δ*t*_1_ and Δ*t*_2_ in a predefined ratio, given by tan α = Δ*t*_1_/Δ*t*_2_, and the methodology was thus dubbed time-domain increments linked together (TILT).^286^ The projection plane is labeled F*/*F*_3_ (Figure 16), where the F* dimension contains mixed ^1^H and ^13^C frequencies, and therefore it is not displayed as ppm using the standard chemical shift scale. The *F*_2_/*F*_3_ plane (α = 90°) corresponds to the ^1^H,^13^C-HSQC spectrum, and the choice of the tilt angle α depends on the differences in ^13^C chemical shifts; the smaller the chemical shift difference is the larger is the tilt angle needed to bring about a chemical shift displacement significant enough to resolve spectral overlap. By acquiring two experiments, one with a positive tilt angle and one with the corresponding negative tilt angle (typically in the range α = ±10° – ±30°), the pure ^1^H and ^13^C frequencies can be derived by using trigonometric functions, and subsequently the NMR chemical shifts will be obtained.

The TILT methodology was applied to the O-antigen polysaccharide from *Escherichia coli* O147 to resolve spectral overlap using a ^1^H,^1^H-NOESY-^1^H,^13^C-HSQC NMR experiment with a tilt angle α = +10°, facilitating displacement of resonances as a result of the small degree of ^13^C frequencies mixed into the projection plane F*/*F*_3_.^288^ Not only will correlations be resolved by shifting resonances, but correlated peaks that are not shifted to any large extent may also be identified in the TILT spectrum due to the absence of cross-peaks with previously degenerate chemical shifts. For the exopolysaccharide (EPS) from *Streptococcus thermophilus* ST1 that only contains glucose and galactose residues in the repeating unit (RU), the TILT NOESY-HSQC experiment was carried out with α = ±15°, which aided ^1^H and ^13^C NMR chemical shift assignments of the EPS.^191^ It may be noted that the symmetrical diagonal present in a NOESY spectrum no longer exists in the TILT spectra but that a “pseudodiagonal” may be identified in the F*/*F*_3_ plane being dependent on the tilt angle (Figure 16). The TILT approach facilitates time savings in data acquisition of at least an order of magnitude.

#### 5.1.4 Acquisition with Multiple Receivers

Acquiring FIDs from different nuclei can be made more efficient if two or several independent receivers are tuned to the specific Larmor frequencies of the individual nuclei. The experiments may be divided into three categories, viz., (i) parallel, (ii) sequential, and (iii) and interleaved acquisition executed such that several spectra are obtained in a single measurement.^289,290^ The parallel acquisition NMR spectroscopy (PANSY) methodology was shown for 2D NMR spectroscopy experiments, with ^1^H-detection by receiver 1 and ^13^C-detection by receiver 2, in which ^1^H,^1^H-COSY and ^13^C,^1^H-correlated experiments were recorded and detected in parallel.^291^ The resulting spectra showed not only *^n^J*_HH_ and ^1^*J*_CH_ (coupled) correlations but also long-range ^*n*^*J*_CH_ correlations; thus, the NMR experiments would in principle be useful for resonance assignments of mono- and disaccharides. A 2D NMR experiment with sequential acquisition is the combination of ^1^H,^1^H-TOCSY and ^13^C,^1^H-correlated experiments in which the latter ^13^C-detected FID is recorded during the spin-lock period (122 ms) of the first one, which leads to a ^1^H-decoupled ^13^C,^1^H-HETCOR spectrum from which ^1^*J*_CH_ correlations are readily identified.^291^

**Figure 17:**
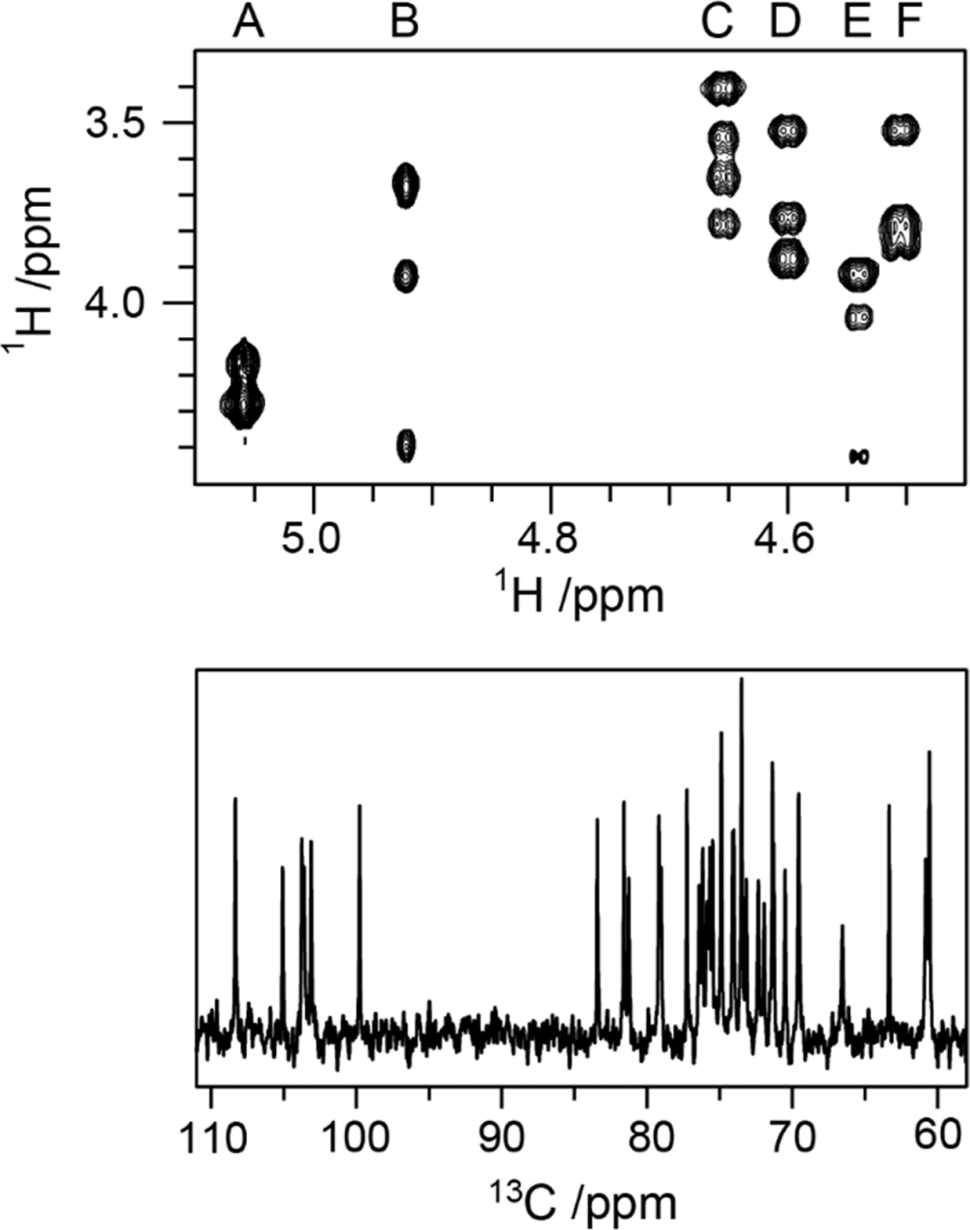
Spectra from a PANSY NMR experiment utilizing dual receivers. The ^1^H,^1^H-TOCSY spectrum, resulting from a 120 ms isotropic mixing time in the experiment, shows correlations from the six anomeric protons of the repeating unit in the ST1 EPS from *Streptococcus thermophilus* (top). During the spin-lock time, a separate one-dimensional ^13^C experiment with proton decoupling was acquired (bottom). Reproduced with permission from ref (191). Copyright 2010 Springer Publisher.

An interleaved experiment utilizing two receivers is the 2D double-COSY experiment in which the ^1^H,^1^H-COSY FID is first recorded, without or with ^19^F-decoupling, followed by the ^19^F,^19^F-COSY experiment detected by the second receiver,^292^ which should be useful for characterization of ^19^F-substituted saccharides.^293^ For the α/β-anomeric mixture of 2-deoxy-2-fluoro-d-glucose the COCOHOESY experiment, in which both experiments share the same *t*_1_ evolution period and consequently the same *F*_1_-axis, was carried out by sequential acquisition whereby the ^1^H,^1^H-COSY FID is recorded by using the first receiver during the HOESY mixing time, followed by recording of the ^19^F FID employing the second receiver, leading to a time-saving by a factor of 2.^292^ Using the ^1^H,^1^H-TOCSY mixing period for decoupling of another spin-1/2 nucleus recorded as a 1D spectrum has been carried out with ^13^C-detection for polysaccharides (Figure 17),^158,191,195^ where the two types of NMR spectra give highly valuable information at the initial stages of a structural elucidation process. For guanosine triphosphate, four receivers were used to simultaneously record ^1^H, ^13^C, ^15^N, and ^31^P NMR spectra,^289^ i.e., from nuclei present in many polysaccharides,^158^ which underscores the potential of acquisition with multiple receivers.

#### 5.1.5 Fast NMR Experiments

**Figure 18:**
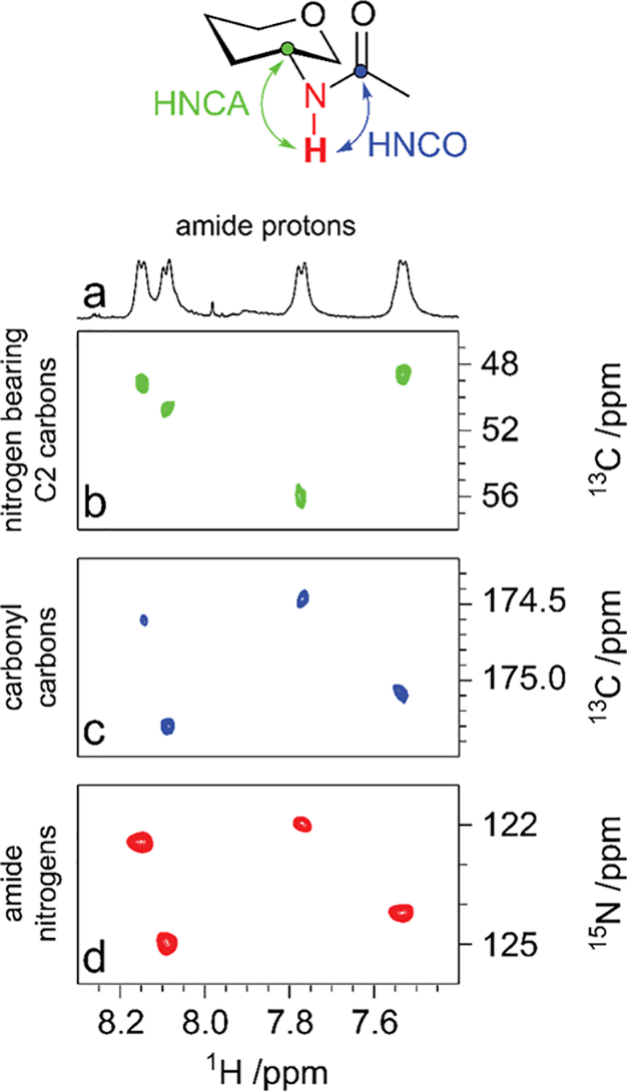
(a) The amide proton region of the ^1^H NMR spectrum of the ^13^C-enriched O-specific polysaccharide from *E. coli* O142. Selected regions of the (b) ^1^H,^13^C plane of the 3D BEST-HNCA, (c) ^1^H,^13^C plane of the 3D BEST-HNCO, and (d) ^1^H,^15^N-SOFAST-HMQC spectra showing correlations from the amide protons. Adapted and reproduced with permission from ref (148). Copyright 2014 Springer.

Recording NMR spectra more efficiently will lead to shorter experimental times and/or higher resolution in spectra. Polarization-enhanced fast-pulsing techniques^294^ have facilitated the acquisition of 2D NMR spectra of [UL-^13^C;UL-^15^N]-labeled proteins in only a few seconds. The ^1^H,^15^N-SOFAST-HMQC band-selective optimized flip-angle short transient heteronuclear multiple quantum coherence (SOFAST-HMQC) experiment as well as the corresponding ^1^H,^13^C-correlated experiment relies on standard data sampling in the indirect *F*_1_ dimension and has been optimized for very short interscan delays.^295^ Notably, the first ^1^H excitation pulse is applied with a flip angle α ≈ 2π/3, which in the HMQC experiment leads to an effective flip angle β ≈ π/3 (cf. Ernst angle excitation), where the selective manipulation targets amide protons in the protein, while leaving all other protons unperturbed, resulting in significantly shortened *T*_1_ relaxation times. The duration of the acquisition time and recycle delay combined, is kept short, ∼ 100 ms, on the order of *T*_1_. Correlation of ^1^H, ^15^N, and ^13^C nuclei by band-selective excitation short-transient (BEST) 3D HNCA and HNCO experiments can be performed in a few minutes using [UL-^13^C;UL-^15^N]-labeled proteins. The ^1^H pulses target again the amide protons and dipolar interactions between these and other unperturbed protons ensure efficient longitudinal relaxation between successive scans, leading to an increased signal-to-noise ratio per unit time. These fast NMR experiments were applied to the ^13^C-labeled O-antigen from *E. coli* O142,^148^ in which four out of the five sugar residues in the RU of the polysaccharide were 2-acetamido-2-deoxy hexoses, i.e., having an *N*-acetyl group at position two of the sugar ring. The three types of experiments successfully revealed anticipated correlations and chemical shifts of the pertinent ^1^H, ^13^C, and ^15^N nuclei (Figure 18); it may be noted that the latter was present at its natural abundance of just 0.37%.

**Figure 19:**
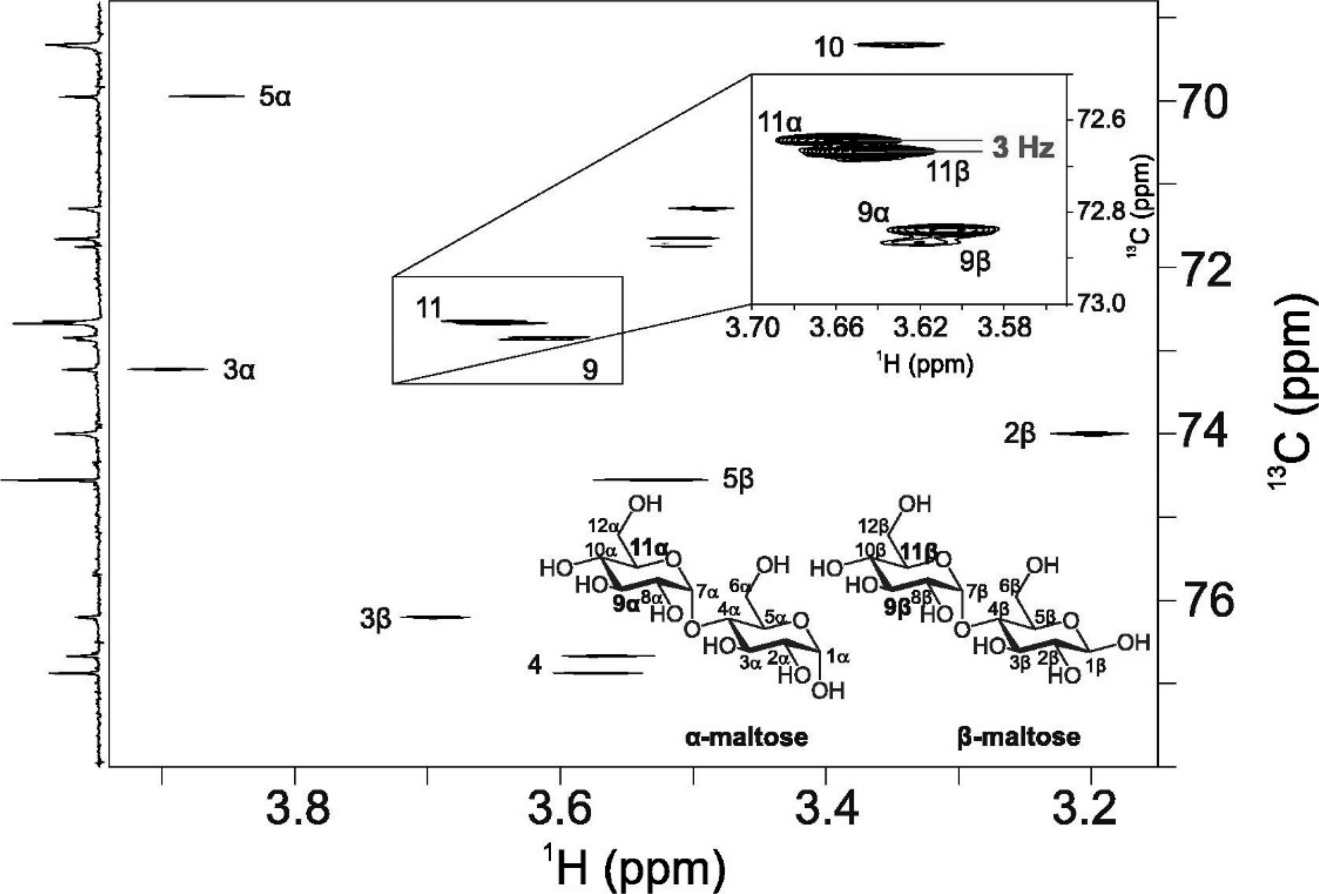
^1^H,^13^C-ASAP-HSQC NMR spectrum of a 200 mM maltose sample in D_2_O. The experiment was acquired in ∼ 7 min using one scan per *t*_1_ increment and 15% NUS sampling. The spectrum was processed using compressed sensing, linear prediction as well as zero filling. The high resolution thus obtained allows for the distinction of cross-peaks from 9α/β and from 11α/β of maltose, which both are approximately 3 Hz apart. Reproduced with permission from ref (298). Copyright 2017 Elsevier.

**Figure 20:**
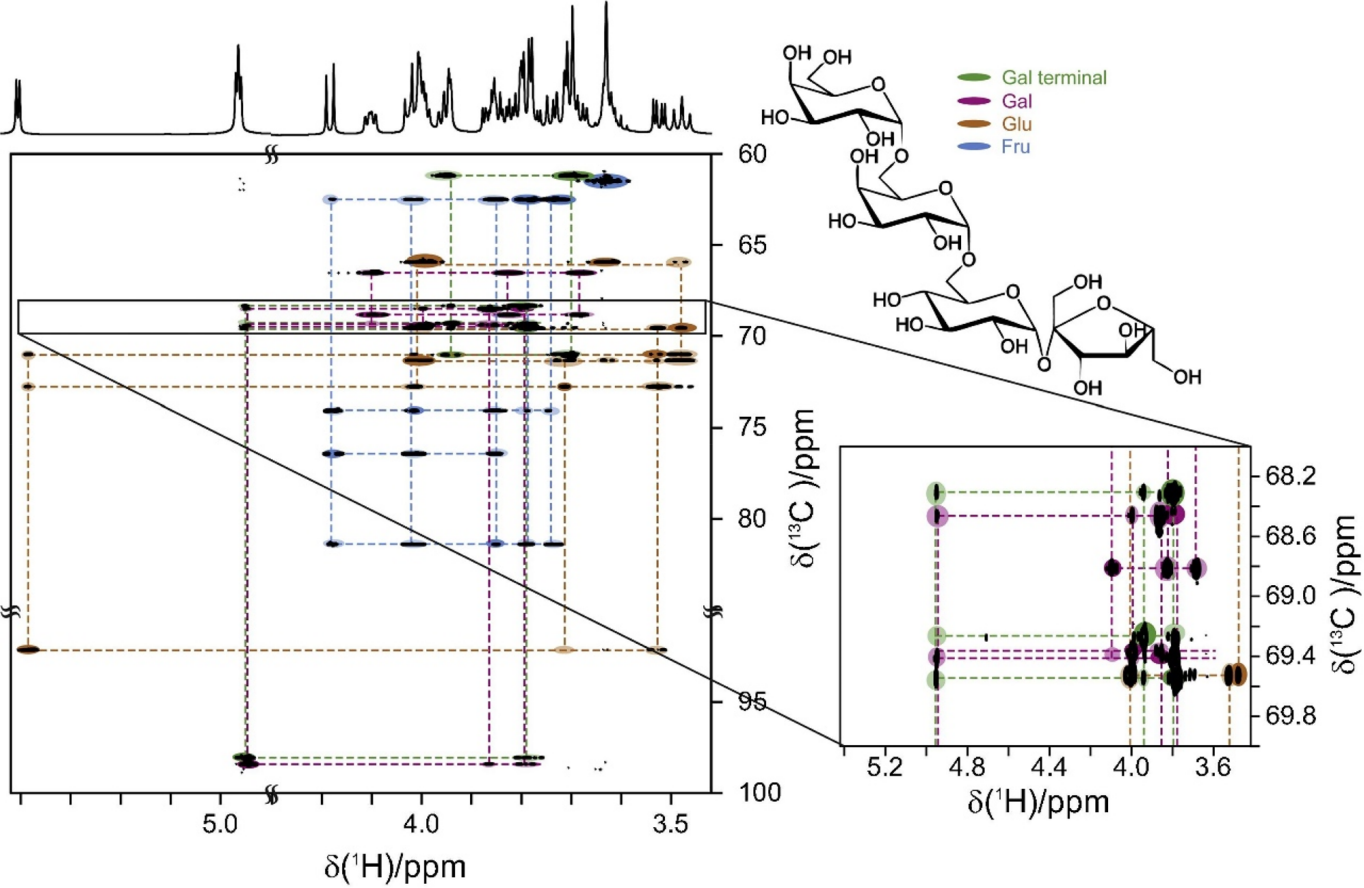
^1^H,^13^C-ASAP-HSQC-TOCSY spectrum of a 250 mM stachyose sample in D_2_O for which 512  ×  1024 (*t*_2_, *t*_1_) data points were recorded. The experiment was acquired using one scan per *t*_1_ increment in ∼ 3.5 min and processed to give a digital resolution in the indirect dimension of 3.7 Hz. The patterns of correlation for the four sugars are highlighted with the color code given next to the structure of stachyose. Reproduced with permission from ref (300). Copyright 2019 Elsevier.

2D ^1^H,^13^C-HMQC NMR spectra may also be recorded more rapidly by having the protons not directly bound to ^13^C nuclei sharing polarization through Hartmann–Hahn mixing with protons at ^13^C sites. This nonselective excitation and short cross-polarization within the spin-coupled network was dubbed acceleration by sharing adjacent polarization (ASAP).^296^ The short mixing sequence with a duration on the order of 40 ms achieves essentially the equivalent result of the relaxation delay *d*_1_ and may partially or in whole replace it. Relying on the polarization sharing principle the ^1^H,^13^C-ASAP-HSQC experiment was proposed, where unused proton magnetization is flipped by a π/2 pulse and stored along the *z* axis during acquisition.^297^ Additional efficiency was achieved by Ernst-angle type excitation, where instead the delays in the initial INEPT transfer module were optimized. The technique facilitates rapid recording of ^1^H,^13^C-HSQC spectra of high resolution in the *F*_1_ dimension when utilizing 15% nonuniform sampling (NUS, vide infra) as shown for the disaccharide maltose (Figure 19).^298^ Further developments led to the ^1^H,^13^C-ASAP-HSQC-TOCSY experiment in which the isotropic mixing sequence was shifted to provide the TOCSY period prior to the acquisition, resulting in both transfer of magnetization through the spin system and fast buildup of polarization sharing, as exemplified for the tetrasaccharide stachyose (Figure 20). It may be noted that in these ASAP experiments the close to continuous high-power pulsing will be demanding for the spectrometer hardware and an undesired heating of the sample may also take place. To mitigate these drawbacks, the extended acquisition time (EXACT) approach has been proposed and implemented in the ^1^H,^13^C-EXACT-ASAP-HSQC experiment in which time periods are introduced where the receiver is gated off.^299^ Broadband heteronuclear decoupling is turned off during the receiver-gated periods, and a pair of ^13^C π pulses are introduced to refocus the heteronuclear coupling during the discontinuity of the acquisition of the FID. The missing data points in the FID are then reconstructed using methods analogous to those used for NUS applications. The EXACT acquisition protocol thus reduces the high duty cycle imposed by the ASAP experiments.

Rapid acquisition of homonuclear correlated spectra can be obtained by a clean in-phase experiment dubbed CLIP-COSY.^301^ The in-phase to in-phase coherence transfer between directly *J* coupled spins relies on the perfect echo^302^ as the mixing element and a duration Δ = 15–25 ms is suitable, although for smaller coupling constants, a longer delay may be required. The CLIP module has been utilized in CLIP-COSY relayed, ^1^H,^13^C-HSQC-CLIP-COSY and ^1^H,^13^C-HSQC-CLIP-COSY relayed experiments applied to tri- and pentasaccharides for NMR resonance assignments.^303^ Furthermore, the possibility to obtain ^1^H,^13^C-HSQC-CLIP-COSY spectra, where cross-peaks have different signs depending on whether they originate from direct HSQC responses or from COSY cross-peaks, have been described as an additional improvement.^304^

Hadamard NMR spectroscopy^265^ offers an efficient way to obtain correlations without incrementing indirect dimensions in *n*-dimensional experiments. Briefly, a Hadamard matrix contains entities of different signs (++ and +– for order 2) that can be combined to extract each encoded component via a decoding scheme. Direct frequency-domain multichannel excitation of NMR signals using an array of radiofrequencies encoded according to a Hadamard matrix^305^ has been used for rapid recording of ^1^H,^1^H-COSY spectra.^306^ The *J* evolution takes place during a soft polychromatic excitation pulse of fixed duration, and at the decoding stage, the components of the FIDs are separated. A number of *N* scans are required to decode the *N* columns of the Hadamard matrix. An order of 8 was used with Gaussian-shaped radiofrequency pulses at separate frequencies with a duration of ∼ 70 ms in the Hadamard ^1^H,^1^H-COSY experiment that was used to assign the five proton resonances from α-d-Glc*p*A-OMe in just 23 s, in contrast to the ^1^H,^1^H-COSY experiment with a duration of > 10 min.^152^ 2D ^1^H,^13^C-HSQC NMR Hadamard transform (HT) experiments on disaccharides using encoding matrices of order 12 or 16 have been performed, leading to acquisition of spectra ∼ 45 times faster than for a regular *t*_1_-incremented experiment.^265,279^ The pure shift ^1^H,^13^C-HSQC NMR HT spectrum of the anomeric mixture of d-glucose was recorded one order of magnitude faster than the conventional 2D NMR experiment, with corresponding signal-to-noise ratios in the two spectra.^307^ Using Hadamard-encoded magnetic transfer (HMT) for ^1^H,^1^H-TOCSY or ^1^H,^1^H-NOESY experiments addressing solely the fast-exchanging labile hydroxyl protons by polychromatic saturations (NOESY only) or looped polychromatic inversions pulses signal-to-noise enhancement of almost one order of magnitude can be obtained, as exemplified for a sialic acid-containing tetrasaccharide.^308^ During these long magnetization-transfer processes, e.g., 600 ms in NOESY experiments, a three-way polarization transfer is effectuated, where the targeted protons are constantly repolarized by water resulting in magnetization transfer to nonlabile protons in the oligosaccharide.

#### 5.1.6 Concatenated NMR Sequences

The developments by which ^1^H,^1^H-COSY and ^1^H,^1^H-NOESY pulse sequences were combined and concatenated into a single 2D NMR experiment were carried out independently (C. A. G. Haasnoot, personal communication) by Haasnoot et al., who dubbed it COCONOSY, and by Gurevich et al., who described it as a combined COSY-NOESY experiment. Interestingly, the two manuscripts were received by the *Journal of Magnetic Resonance* just a few days apart in 1983, and the two articles were published in the same volume of the journal the following year.^309,310^ Notably, the FID from the COSY experiment is collected during the mixing time of the NOESY experiment, whose FID is then acquired. The efficiency and time-saving are in particular due to the fact that the two experiments share a common recovery delay *d*_1_ prior to each subsequent scan of the 2D NMR experiment.

The concept of concatenation NMR experiments beyond the COCONOSY experiment by combining a series of multiple 2D experiments using modules and entangle these into supersequences with only one recovery delay was recently demonstrated, where each acquisition is based on ^1^H detection for optimum sensitivity referred to as NMR by ordered acquisition using ^1^H-detection (NOAH).^311^ Both heteronuclear ^1^H,X- and homonuclear ^1^H,^1^H-correlation modules can be incorporated as part of the supersequence that uses samples at natural isotope abundance. The least sensitive module is typically placed first in the sequence and bulk magnetization that is not used, is as far as possible preserved for subsequent NOAH sequence modules by keeping it along the +*z* axis. By extending the COCONOSY experiment by additional modules, where FID detection is carried out after each one, a NOAH-4 supersequence can be made by, e.g., a ^1^H,^15^N-HMQC followed by a ^1^H,^13^C-HSQC, a ^1^H,^1^H-COSY, and a ^1^H,^1^H-NOES*Y* experiment denoted MSCN; a single letter notation is used for basic experiments and, when required, sub- and superscripts denote nuclei or subtype experiments, respectively.

**Figure 21:**
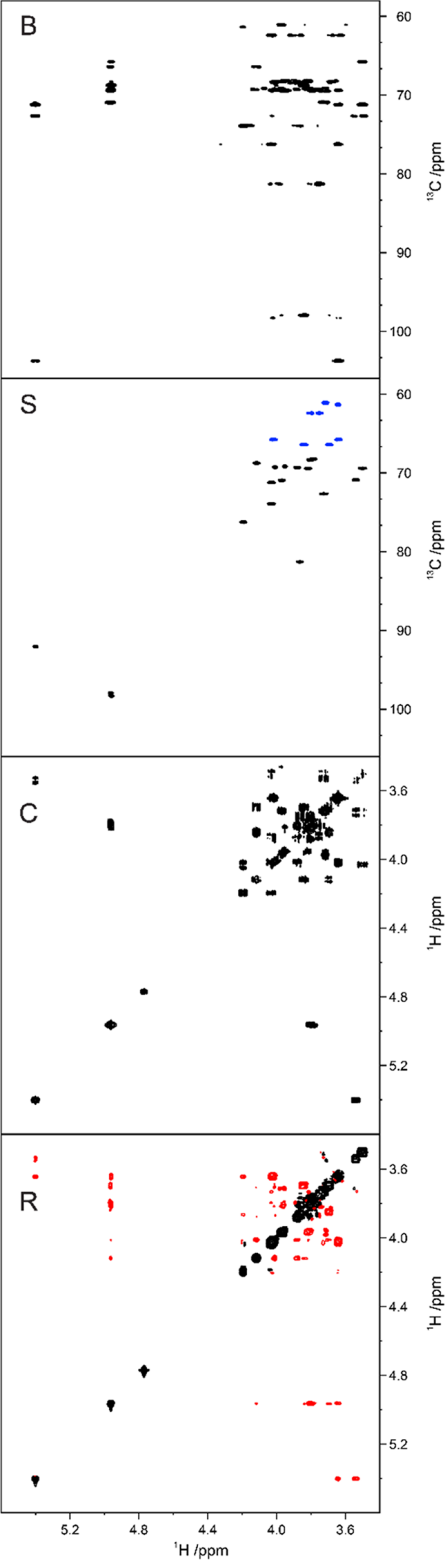
NMR by ordered acquisition using ^1^H-detection (NOAH-4) supersequence BSCR records 2D spectra in a single experiment: (B) ^1^H,^13^C-HMBC, (S) multiplicity-edited ^1^H,^13^C-HSQC (cross-peaks from hydroxymethyl groups, at δ_C_ < 67, are shown in blue color), (C) ^1^H,^1^H-COSY, and (R) ^1^H,^1^H-ROESY with a mixing time of 300 ms (cross-peaks from dipolar interactions are shown in red color). The tetrasaccharide stachyose with a concentration of 48 mM in D_2_O was used for the experiment, which was performed in 22 min on a 600 MHz NMR spectrometer equipped with a cryoprobe.

The number of possible combinations for a NOAH experiment becomes very large as additional modules are added, although not all permutations are suitable from an NMR experimental point of view. The efficiency of the technique was exemplified on sucrose with NOAH-2 (SC), NOAH-3 (SCN), and NOAH-4 (SBCN).^311^ Another NOAH-4 experiment (BSCR) that gives a good deal of spectral and structural information for carbohydrate molecules is exemplified for the tetrasaccharide stachyose (Figure 21); note the vertical presentation of the 2D NMR spectra highlighting that all the experiments were ^1^H-detected using a single channel. The NOAH experiments can be optimized in different ways, e.g., by ordering and in the BSC experiment the initial part uses a *zz*-filter for the HMBC module acting as a π/2 excitation pulse on protons bound to ^12^C, while the protons bound to ^13^C are left along the +*z* axis.^312^ Furthermore, by applying a short spinlock^296^ to the COSY module prior to the start of the experiment differences in peak intensity due to variation in the longitudinal relaxation rates can be reduced^313^ as well as to decrease the presence of fast pulsing artifacts.^312^ Further developments of NOAH experiments include using nonuniform sampling schemes^313^ and multiple receivers.^314,315^

By recording the NOAH experiments in parallel^316^ using time-sharing schemes^317^ in conjunction with the sequential data acquisition, the efficiency of the single measurement using ten modules was further improved. The time-shared modules for the *p*-NOAH-10 included, e.g., HSQC-COSY/HSQC, HSQC-TOCSY/HSQC, and IP-HSQC/AP-HSQC, and interleaved modules such as HMBC and TOCSY with different sets of mixing times in the heteronuclear and homonuclear experiments, respectively, as well as COSY and ROESY experiments. A tailor-made *p*-NOAH-5 supersequence relying on ^1^H and ^13^C nuclei, BS^C^S^J^T/S, consisting of HMBC, multiplicity-edited HSQC-COSY, *F*_2_-coupled HSQC, TOCSY, and multiplicity-edited HSQC was developed, resulting in NMR spectral data optimal for use with the structural elucidation program CASPER^318^ and presented using a methyl glycoside of the milk oligosaccharide lacto-*N*-neotetraose.^316^ Moreover, using a room temperature inverse detection probe a *p*-NOAH-10 experiment was carried out on a cyclic peptide at a concentration of 50 mM in less than 10 min. As different applications require different NMR experiments, the modular program generation of supersequences in silico (GENESIS) has been created, which systematically produces NMR pulse programs for arbitrary NOAH supersequences.^319^

A reminiscent approach to obtain two or more 2D NMR spectra from a single scan sharing the same *t*_1_ evolution period is to use multiple-FID acquisition and has been dubbed MFA.^320^ The technique was implemented for multiple relay ^1^H,^1^H-COSY experiments, where subsequent to the initial COSY experiment a pulse sequence train of [Δ – π/2]_3_ was applied, where Δ is the proton–proton relay transfer time (∼90 ms) during which also the FID is acquired. Each transfer step is stored and analyzed separately, and the stepwise development of cross-peaks in the 2D spectra can conveniently be followed as was shown for sucrose. The MFA methodology was additionally used to construct ^1^H,^1^H-COSY/TOCSY, ^1^H,^13^C-HMBC/HMBC-COSY and ^1^H,^13^C-HMBC/HMBC-TOCSY experiments. Furthermore, the MFA approach has been used in interleaved dual NMR acquisition of equivalent transfer pathways whereby each magnetization component is monitored by independent FIDs starting from either a ^1^H,^1^H-TOCSY or a ^1^H,^13^C-HSQC experiment.^321^ The methodology is based on the fact that, after the first acquisition of the FID (transverse plane component), the second nonobservable I_*z*_ component is utilized by a DIPSI-2 spin-lock for a TOCSY module or rotated to the transverse plane to be utilized in an HSQC module. The MFA experiments that have been created in this way are, e.g., ^1^H,^1^H-TOCSY/TOCSY, ^1^H,^13^C-HSQC/pure shift HSQC, ^1^H,^13^C-HSQC/HSQC-TOCSY, and ^1^H,^13^C-HSQC(*F*_2_-coupled)/HSQC, where for the latter dual experiment the *F*_2_-coupled spectrum is recorded first due to the intrinsically higher sensitivity of the decoupled version and that reduction in sensitivity takes place for the second FID due to translational diffusion of the molecules during acquisition of the first FID.

Concatenation of SEA X-LOC, which can distinguish between two- and three-bond correlations based on different multiplet widths in the indirect dimension,^322,323^ with H2OBC,^271^ thus sharing the relaxation delay, *d*_1_, can provide complete correlation maps, as shown for an *O*-methylated and *O*-sulfated trisaccharide with a complex substitution pattern.^324^ The concatenation approach was further extended to give a SEA XLOC-HMBC-H2OBC experiment for resonance assignments based on heteronuclear one-bond and long-range correlations.^325^ Interestingly, the modules of these experiments were subsequently utilized so that the magnetization of the first experiment relaxes toward equilibrium during the second one and vice versa. The experimental approach was dubbed NO relaxation delay (NORD),^326^ devoid of the commonly used relaxation delay, *d*_1_, and the NORD HMBC-H2OBC experiment was used for NMR spectral analysis of tri- and pentasaccharides.

#### 5.1.7 Pure Shift Experiments

One-dimensional ^13^C NMR experiments are most often acquired with broadband ^1^H-decoupling and 2D ^1^H,^13^C-HSQC experiments are typically acquired such that the heteronuclear ^1^*J*_CH_ couplings are refocused and broadband ^13^C-decoupled in the *F*_1_- and *F*_2_-dimensions, respectively. In the ^1^H NMR spectrum of a glycan, the homonuclear scalar coupling constants are a source of important structural information, but the low dispersion of signals from carbohydrate compounds in conjunction with the ^n^*J*_HH_ couplings further complicate resonance identification and assignments. The severe signal overlap in ^1^H NMR spectra of carbohydrates may be mitigated by homonuclear broadband (HOBB) decoupling as devised by Zangger and Sterk, who demonstrated the technique on sucrose.^264^ The original ZS experiment to obtain pure shift ^1^H NMR spectra is based on selective pulses and weak pulsed-field-gradients (PFGs), where each chemical shift arises from a different slice of the sample and a delay is incremented in the pseudo 2D experiment. Midway between excitation and acquisition, a homodecoupling block is implemented consisting of a combination of a nonselective π pulse and a selective inversion element in the presence of a PFG that only affects the active spins and leads to refocusing of the homonuclear coupling(s) between the active and the passive spins.^327,328^ At the beginning of the FID, the effects of *J* coupling have been refocused, and on the order of 32 data points are collected and saved as chunks. From each subsequent increment, a chunk is collected and 32–64 chunks are then concatenated to a new FID, followed by FT, resulting in a HOBB decoupled ^1^H NMR spectrum.

However, the need to reconstruct the FID poses practical problems in processing. This may be alleviated by instead using an approach whereby the acquisition is interrupted by decoupling blocks, approximately every 1/3(*^n^J*_HH_), which makes it possible to acquire the FID in real time essentially like in a regular 1D ^1^H NMR spectrum.^329^ In the ZS experiment, during the acquisition for a short period of time amounting to chunks of a few tens of ms a small amount of *J* evolution will take place. This causes a weak modulation of the signal with a period of 1/*sw*_1_, where *sw* is the width of the spectrum and *sw*_1_ is an integer submultiplier of *sw*. On FT, the spectrum will show small artifact sidebands periodically showing up at intervals *sw*_1_. The ZS-based NMR experiment sideband averaging by periodic phase incrementation of residual *J* evolution (SAPPHIRE) suppresses these sideband artifacts by manipulating the phase of the modulation by small changes in timing, such that an extra echo can be shifted slightly forward and backward in time as part of an averaging process, leading to ultraclean pure shift NMR spectra.^330^ To carry out the HOBB decoupling in a ZS experiment for *J* coupled spin-pairs with small chemical shift differences, long and highly selective pulses are required to obtain narrow individual slices along the NMR tube. This limitation, as well as HOBB decoupling for strongly coupled spin systems, may be resolved by utilizing a perfect echo in conjunction with the ZS experiment, referred to as perfect echo pure shift improved experiment (PEPSIE).^331^

In the pure shift yielded by chirp excitation (PSYCHE) NMR experiment, which belongs to the ZS class of broadband-decoupled ^1^H NMR experiments, all spins in the sample are irradiated and the refocusing of *J* couplings is carried out by low flip angle (β) swept-frequency pulses in the presence of weak PFGs, whereby the effect of the two β pulses is to refocus the active spins in a stimulated echo, whereas the passive spins are unaffected.^332,333^ Furthermore, as the frequency-swept pulses and the concomitant PFGs effectively lead to different ZQC evolution times throughout the sample, effects of ZQCs are suppressed. The extension to a 1D selective TOCSY-PSYCHE experiment makes it possible to differentiate peaks separated by just a few parts per billion because in the resulting pure shift ^1^H NMR spectrum, only resonances from a spin-system originating from a specific chemical shift will be identified, a finding that otherwise would have gone unnoticed in the PSYCHE spectrum, despite the fact that homonuclear *J* coupling had been removed.^334^ Furthermore, by combining the two modules in reverse order to the above, a 2D *F*_1_-PSYCHE-TOCSY experiment can be devised in which the homonuclear *J* evolution during *t*_1_ will be suppressed.^335^ This then facilitates conventional acquisition during *t*_2_, allowing for high resolution in the *F*_2_ dimension, where the multiplet structure of cross-peaks will remain. Notably, to benefit from the decoupling in the *F*_1_ dimension, a large number of *t*_1_ increments should be used. Subsequent indirect covariance processing can generate a 2D ^1^H,^1^H-TOCSY spectrum where all couplings have been removed and cross-peaks are singlets in both dimensions. The covariance processing technique has also been used to obtain decoupled 2D NMR spectra in both dimensions from *F*_2_-ZS-NOESY and CT-*n*QF-COSY experiments, where the latter is a multiple quantum-filtered constant time experiment resulting in decoupling in the *F*_1_-dimension, whereas the former results in decoupling in the *F*_2_-dimension by concatenating the NOESY sequence with a ZS block during the *t*_2_ period.^336^

**Figure 22:**
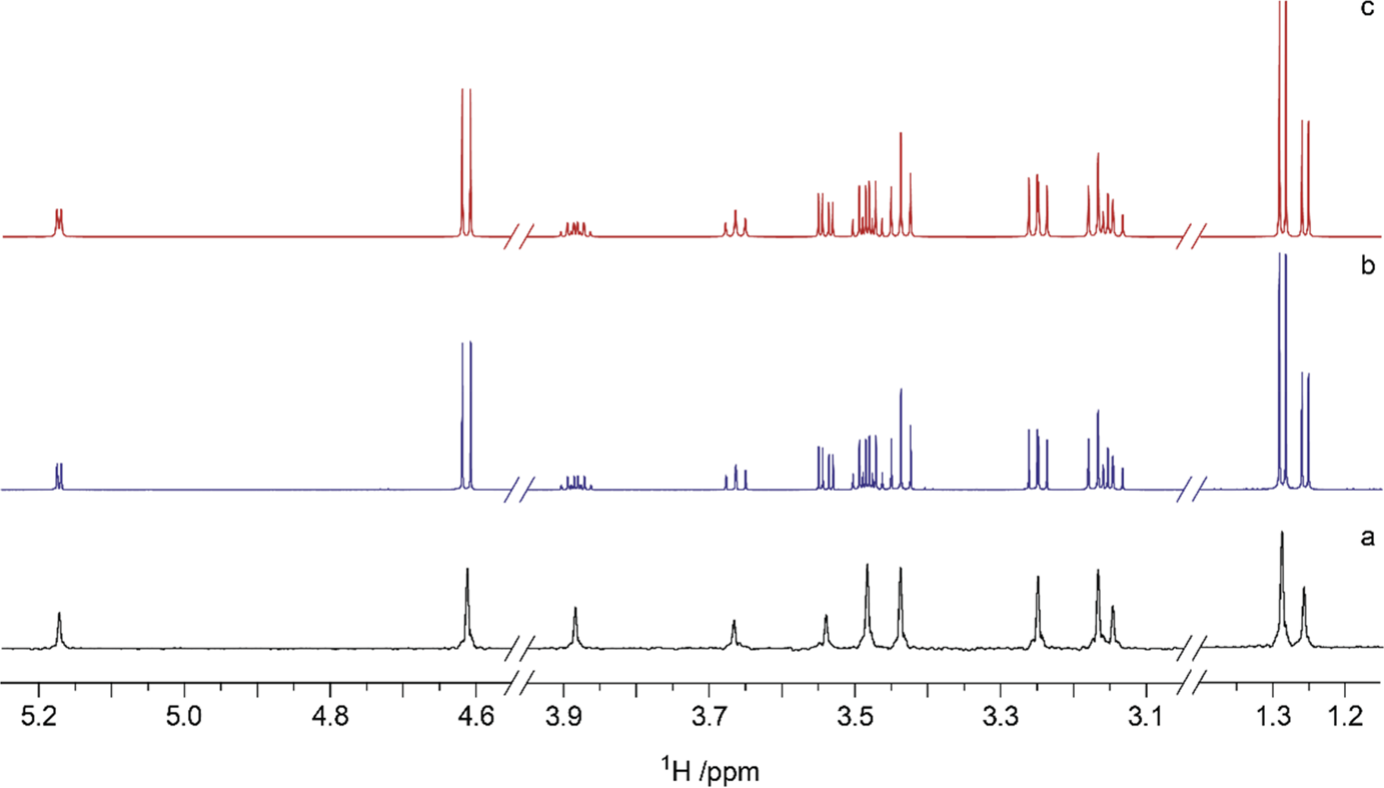
^1^H NMR analysis at 700 MHz of d-Qui (6-deoxy-d-glucose) in D_2_O 70 °C. The monosaccharide is present in the pyranose ring form with an anomeric α:β ratio of 1:2. Highlighted regions of (a) the experimental pure shift ^1^H spectrum (black), (b) the experimental ^1^H spectrum (blue), and (c) the corresponding simulated ^1^H spectrum by total-line shape analysis using the PERCH NMR software (red). Reproduced with permission from ref (140). Copyright 2013 Elsevier.

**Figure 23:**
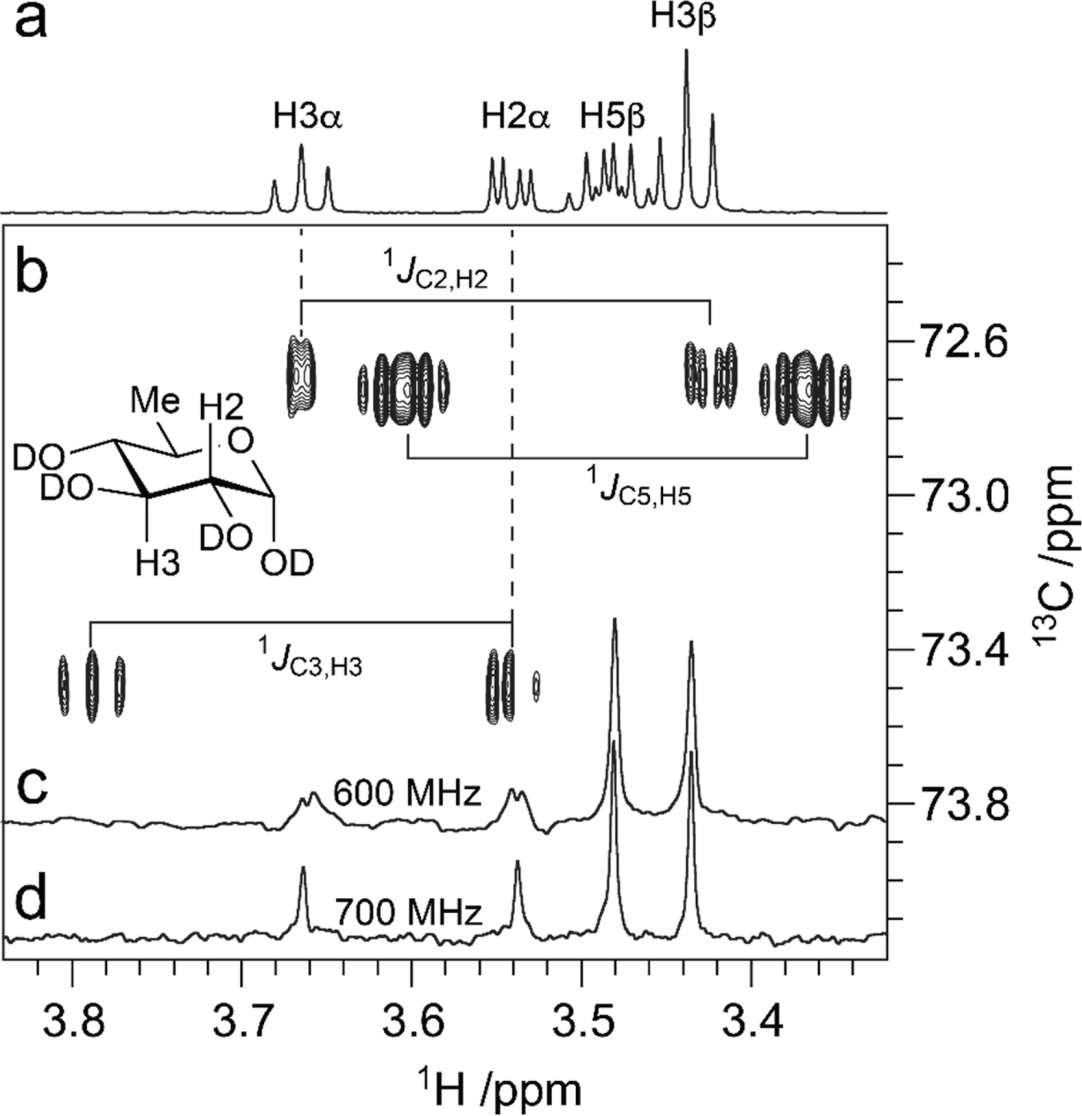
(a) Selected region of the 1D ^1^H NMR spectrum of d-Qui*p* showing the H2 and H3 resonances of the α-anomeric form (minor) and H3 and H5 resonances of the β-anomeric form (major). (b) Selected region of the ^13^C-coupled ^1^H,^13^C-HSQC spectrum showing one-bond proton–carbon correlations from the H2α, H3α, and H5β protons. The spectra of (a,b) were both recorded at a ^1^H frequency of 600 MHz. In the 2D NMR spectrum, strong coupling artifacts are observed for the higher frequency component of the ^13^C-coupled H2α resonance, and the lower frequency component of the ^13^C-coupled H3α resonance; these respective signals are ^3^*J* coupled to H3α and H2α protons attached to ^12^C atoms (see dashed lines). Note that this strong coupling phenomenon occurs at this specific magnetic field because Δ(ν_H3α_ – ν_H2α_) ∼ ^1^*J*_C3α,H3α_/2 ∼ ^1^*J*_C2α,H2α_/2. Comparison of pure shift ^1^H NMR spectra recorded at a ^1^H frequency of 600 MHz (c) and 700 MHz (d) using the ZS real-time BIRD pulse sequence described by Aguilar et al.,^337^ employing 64 data chunks; the spectral region is the same as that in (a). Note that in the pure shift ^1^H spectrum of (c), the H2 and H3 resonances of α-d-Qui*p* are not fully homodecoupled due to strong coupling effects mentioned above.

**Figure 24:**
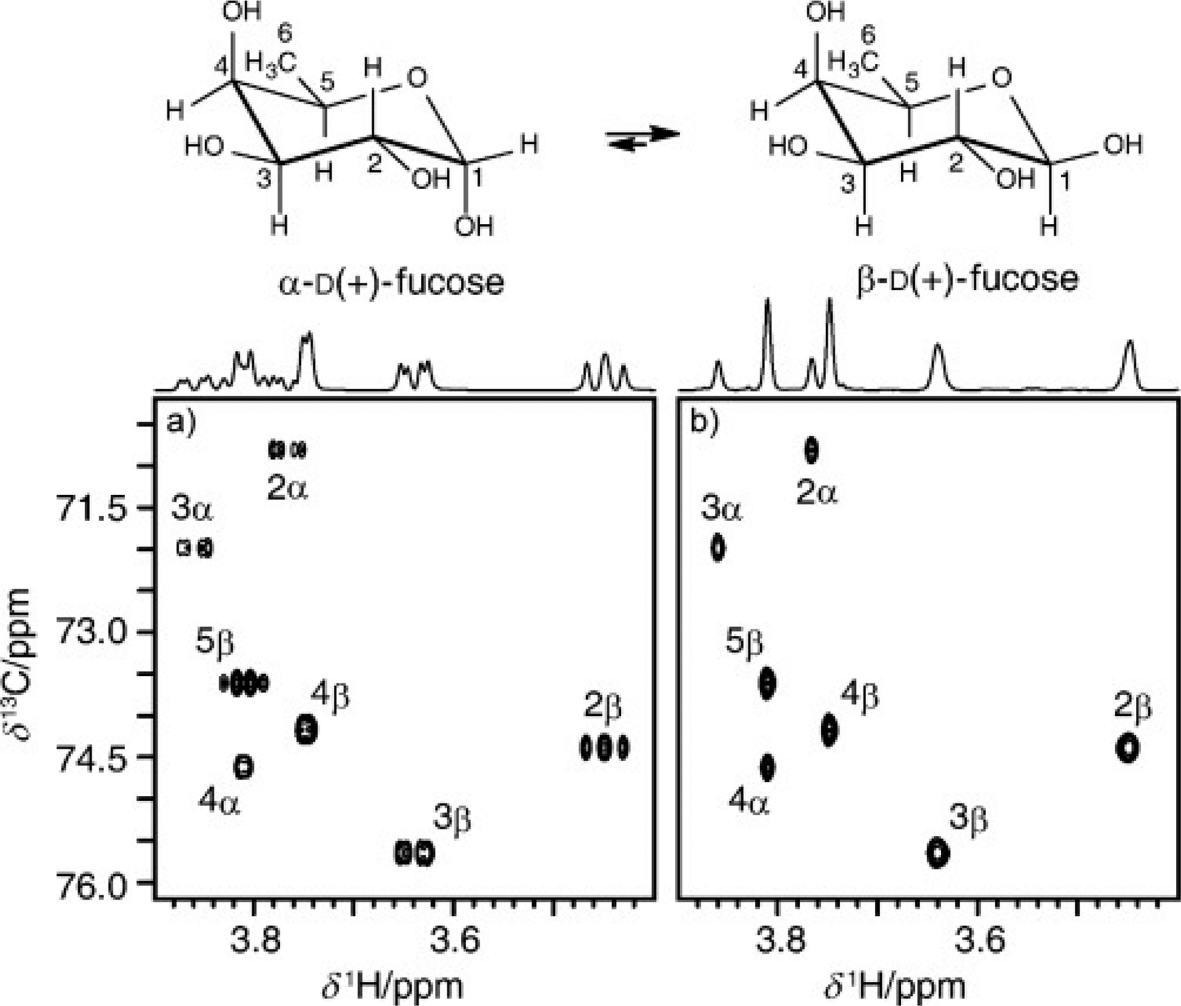
Selected regions of ^1^H,^13^C-HSQC spectra of d-fucose in D_2_O: (a) conventional gHSQC and (b) real-time pure shift gHSQC. 1D traces are integral projections onto the *F*_2_ (^1^H) axis. Reproduced with permission from ref (339). Copyright 2013 Wiley.

Pure shift 1D ^1^H NMR spectra can alternatively be obtained by incorporating a BIRD module into the pulse sequence, which relies on utilizing the low natural abundance ^1^H,^13^C spin-pairs in a ZS-type pseudo 2D fashion, where halfway during the *t*_1_ evolution period, a ^1^H π pulse and the BIRD pulse sequence element have the effect that the *J* evolution of the passive spins (protons on ^12^C) is reversed during the second half of the *t*_1_ evolution period. Heteronuclear coupling of the ^1^H,^13^C spin-pairs will then be refocused at the beginning of the acquisition and homonuclear couplings refocus at 1/(2*sw*_1_); broadband ^13^C decoupling is applied during the acquisition period. Concatenation of the acquired data with different *t*_1_ evolution periods then leads to a 1D HOBB decoupled ^1^H NMR spectrum, as illustrated for *n*-hexanol^337^ and exemplified herein for d-quinovose (Figure 22a). However, the effects of strong coupling can be severe if one of the ^1^H,^13^C satellite components is *J* coupled to another proton, of which there are 99% bound to ^12^C nuclei. If the ^1^H NMR chemical shifts of both of these protons are degenerate at a specific spectrometer frequency, strong coupling can lead to artifacts in spectra acquired by pure shift ZS real-time BIRD experiments as observed for the H2 and H3 resonances from α-d-quinovose at a ^1^H NMR frequency of 600 MHz but not at, e.g., 700 MHz (Figure 23). Another approach using instead HOBB one-shot BIRD decoupling in a 1D fashion employing an initial INEPT-type isotope filter to select for ^13^C-bound protons followed by an array of looped BIRD-modules and acquisition blocks in a 1D NMR experiment also results in a pure shift ^1^H NMR spectrum. The importance of using frequency-swept ^13^C pulses and efficient heteronuclear decoupling during the experiment was stressed in order to obtain maximum resolution of signals in the HOBB decoupling experiment.^338^ It may be noted that a limitation of the BIRD approach is that proton decoupling fails for diastereotopic protons bound to the same ^13^C nucleus, where they exhibit a geminal ^2^*J*_HH_ coupling, such as for H6_pro-*R*_ and H6_pro-*S*_ of glucose, as illustrated in the latter study. In contrast to pure shift ^1^H NMR spectra relying on the natural abundance ^1^H,^13^C spin-pairs, the 2D ^1^H,^13^C-HSQC NMR experiment can be performed without loss in sensitivity, and the acquisition is performed in real time using a windowed scheme. Acquisition is looped *n* times with *n* ≈ 30 under ^13^C-decoupling and a *J* refocusing element, consisting of a BIRD module and a ^1^H π pulse, is inserted midway during the acquisition period. The resulting pure shift 2D ^1^H,^13^C-HSQC NMR spectrum of d-fucose shows well resolved cross-peaks devoid of homonuclear ^1^H,^1^H couplings in comparison to the conventional counterpart (Figure 24).^339^ Additional improvements and recommendations have been made to decrease artifacts resulting from the chunked acquisition in order to obtain high-quality data for these types of real-time pure shift NMR experiments.^340^

#### 5.1.8 Isotope Labeled Oligo- and Polysaccharides

Stable isotope labeling or enrichment in oligo- or polysaccharides have relied in particular on ^13^C and/or ^15^N nuclei for structural studies and analysis of NMR chemical shifts. These key isotopes were incorporated into the glycosaminoglycan polysaccharide hyaluronan (HA), [→4)-β-d-Glc*p*A-(1→3)-β-d-Glc*p*NAc-(1→]*_n_*, using an *E. coli* transfected with a recombinant HA synthase, ^15^NH_4_Cl and d-[UL-^13^C_6_]glucose.^157^ The shed polymeric material was purified and digested to give after purification ^13^C,^15^N-isotopically labeled even-numbered HA_4_–HA_12_ oligosaccharides with *N*-acetyl-d-glucosamine at the reducing end. In the 2D ^1^H,^1^H-TOCSY spectra of HA_6_ only cross-peaks from NH resonances of the reducing end residue (in a mixture α- and β-anomeric forms) could be resolved. From a 3D ^1^H,^15^N-TOCSY-HSQC experiment not only the resonances emanating from the reducing end residue but also the internal as well as d-Glc*p*NAc resonances in the terminal disaccharide element of HA_6_ were possible to identify. The ^13^C- and ^15^N-labeling permitted resonance assignments for the *N*-acetyl groups of HA_6_ using the triple resonance experiments HNCA and HNCO (vide infra) commonly used in NMR studies of ^13^C,^15^N-labeled proteins; in this particular HNCA experiment, standard ^13^C *t*_1_-incrementation was used to allow for a long acquisition time, resulting in ^1^*J*_CC_ coupled multiplets. Subsequent studies of the [UL-^13^C;^15^N]-labeled HA_4_ and HA_6_ oligosaccharides focused on exploring the unique ^13^C NMR chemical shifts and *J* couplings of the carboxylate moiety of the β-d-Glc*p*A residues in order to filter out coherences from β-d-Glc*p*NAc residues.^341^ The 2D NMR experiments of the “out and back” type correlate via one-bond couplings the carboxylate carbon C6 with C5 and H5 (HC^5^C^6^ experiment) or C6 with C1 via a long-range coupling ^3^*J*_C1,C6_ ≈ 5 Hz (cf. the significant cross-peak between C1 and C6 in the ^13^C,^13^C-COSY spectrum from the terminal β-d-glucopyranosyl residue of [UL-^13^C_12_]cellobiose, Figure 39, vide infra), and H1 (HC^1^C^6^ experiment). An additional modification by insertion of a ^1^H,^1^H-TOCSY block with a mixing time duration of ∼ 40 ms in the HC^5^C^6^ experiment resulted in correlations between C6 of the carboxylate group and H2–H5 protons within the same residue (HC^5^C^6^-TOCSY experiment).

^13^C-Direct detection NMR experiments of partially or uniformly enriched biomolecules facilitates, inter alia, identification of quaternary carbons without additional transfer steps to adjacent protons, whether one-bond or over multiple bonds, as well as avoiding solvent suppression schemes.^342^ For ^13^C NMR resonance assignments of oligo- and polysaccharides, a number of NMR experiments are available, making use of the fact that for glycopyranose residues ^1^*J*_CC_ ≈ 45 Hz which facilitates homonuclear correlations of uniformly labeled glycans to be traced via ^13^C nuclei by relying on either ^13^C-based experiments only (oligosaccharides), or “proton-start” (polysaccharides in particular) implementation.^148^ Thus, the ease of magnetization transfer can be used to unravel the spin-systems using ^13^C,^13^C-COSY or ^13^C,^13^C-TOCSY experiments with short mixing times, τ_mix_ = 5–20 ms, in the latter case.

**Figure 25:**
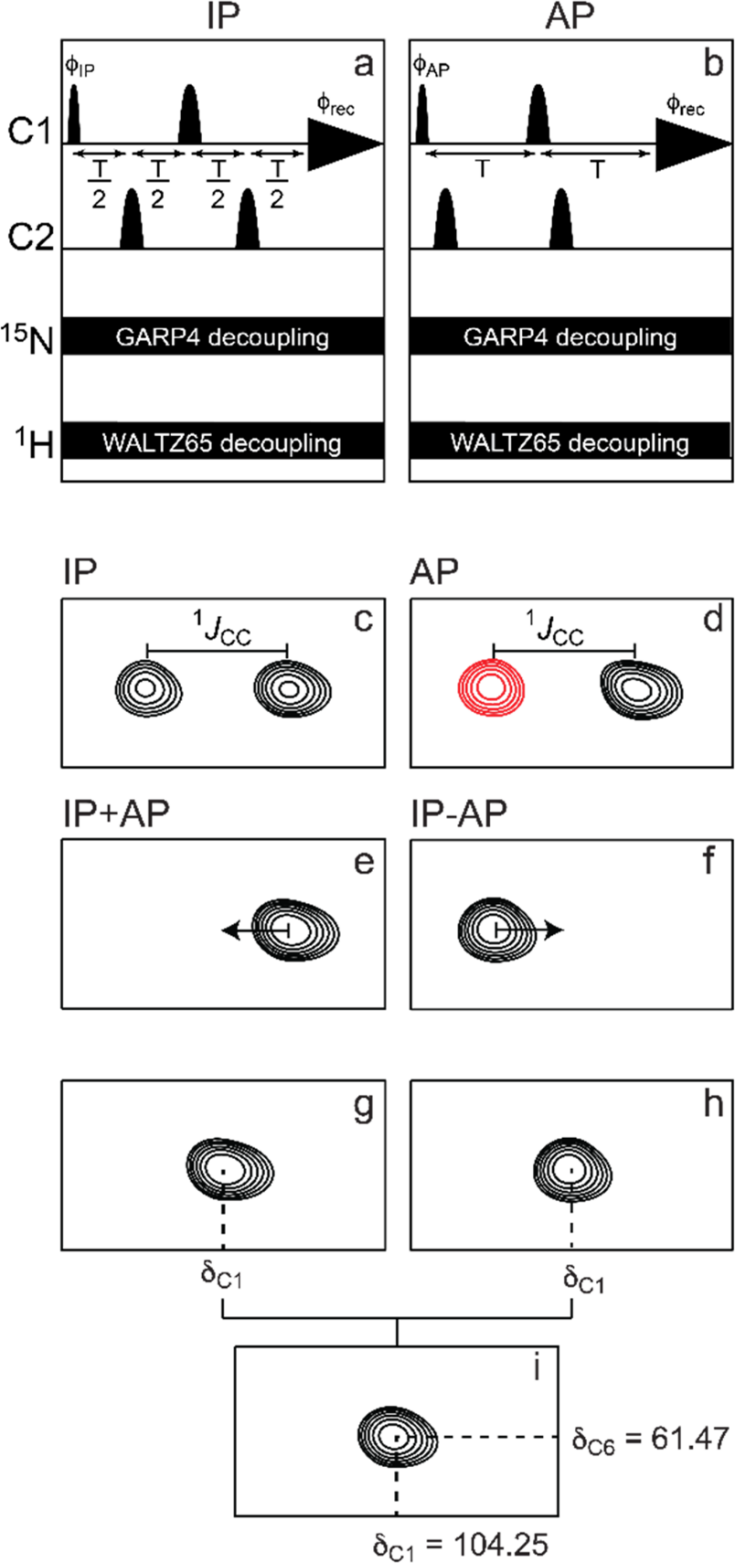
Graphic representation of (a) in-phase (IP) and (b) antiphase (AP) schemes employed in the virtual decoupling of the ^13^C,^13^C-TOCSY spectrum of the ^13^C-enriched O-antigen PS of *E. coli* O142,^148^ where *T* = 1/4·^1^*J*_CC_. Selected region of the IP (c) and AP (d) ^13^C,^13^C-TOCSY spectra (τ_m_ = 20 ms) showing the cross-peak correlation between the C6 and C1 of the β-d-Glc*p*NAc residue. In (d) the cross-peak in red color has an opposite sign than the cross-peak indicated in black. (e,f) Spectra resulting from the linear combinations of the IP and AP spectra of (c,d). The cross-peaks are then shifted downfield (e) and upfield (f) by 0.5·^1^*J*_CC_; the resulting spectra (g and h, respectively) are added up to achieve the homonuclear virtual decoupled spectrum (i).

**Figure 26:**
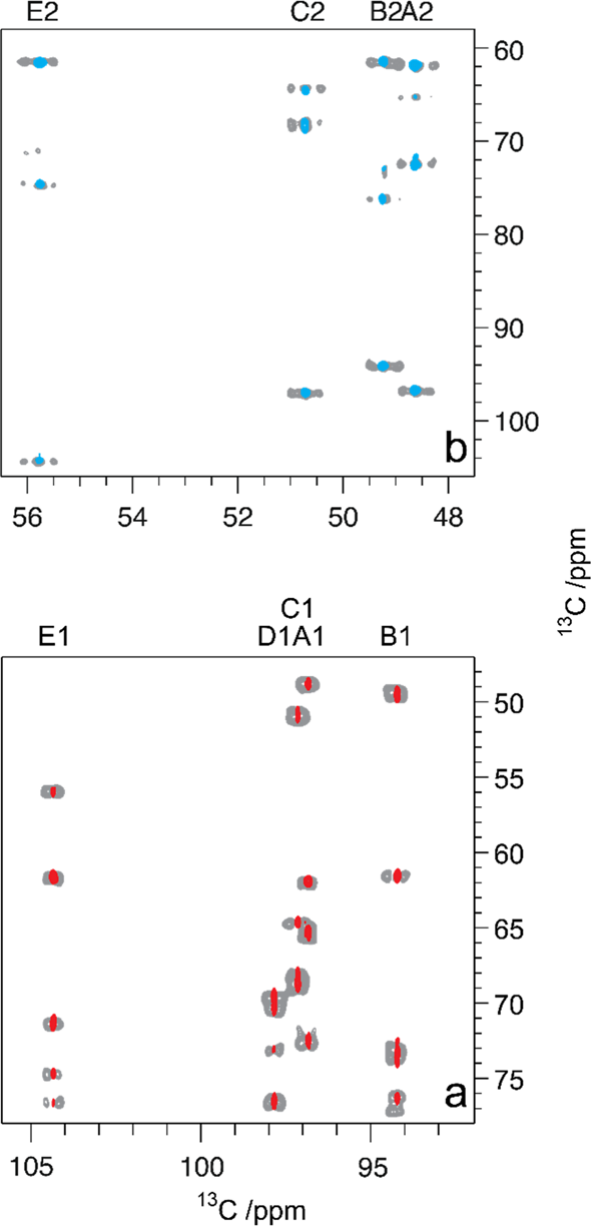
Overlay of selected regions of ^13^C,^13^C-TOCSY spectra (τ_m_ = 20 ms) of the ^13^C-enriched O-antigen PS of *E. coli* O142 showing correlations from (a) anomeric carbons and (b) nitrogen-bearing C2 carbons. The spectrum recorded using the IPAP scheme with virtual decoupling of ^1^*J*_C1,C2_ in the direct dimension is shown in red color, whereas the spectrum recorded using the DIPAP scheme with simultaneous decoupling of ^1^*J*_C1,C2_ and ^1^*J*_C2,C3_ in the direct dimension is shown in cyan color. The classical ^13^C,^13^C-TOCSY spectrum is shown in gray color.

**Figure 27:**
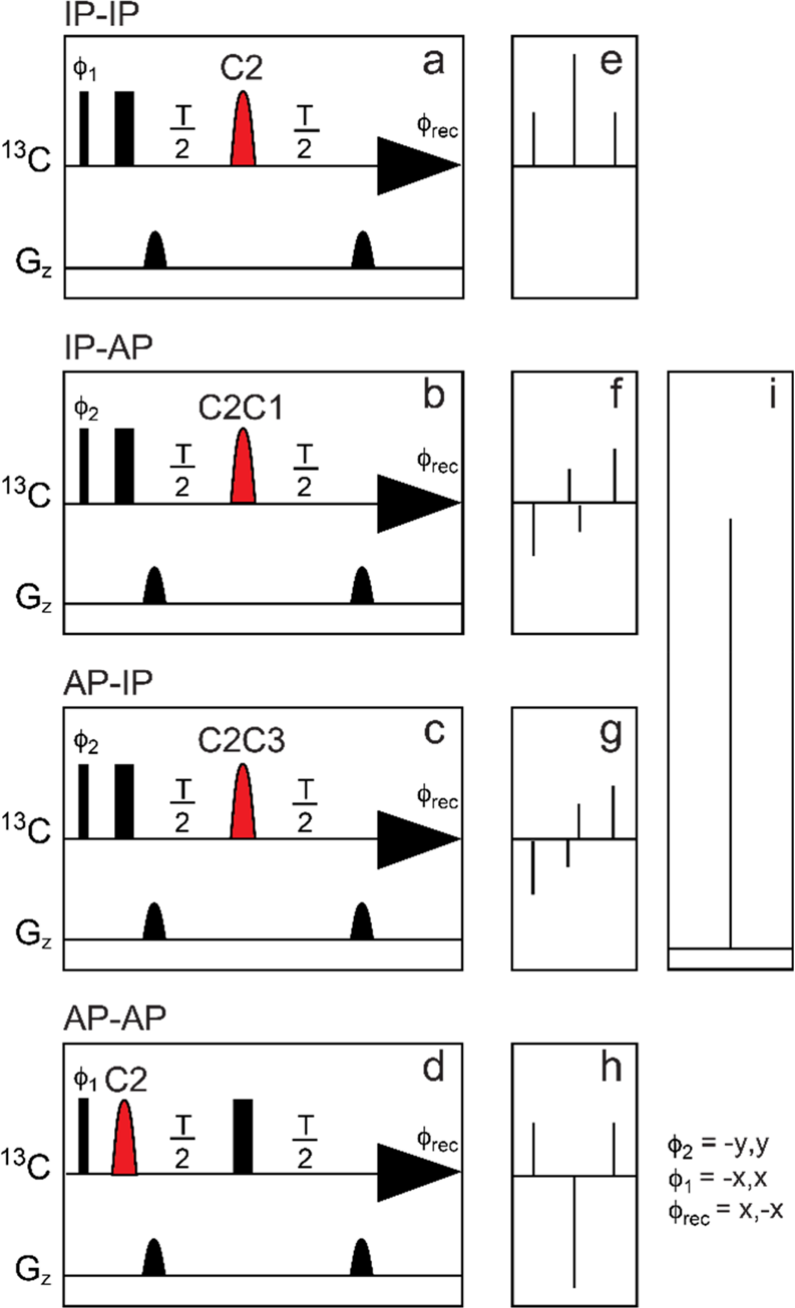
Graphic representation of the double in-phase antiphase (DIPAP) schemes added to the ^13^C,^13^C-TOCSY experiment for the virtual decoupling of the ^1^*J*_C1,C2_ and ^1^*J*_C2,C3_ couplings of nitrogen bearing C2 carbons resonances.^148^ During this experiment, four different spectra (e–h) with different magnetization components (IP-IP, IP-AP, AP-IP, and AP-AP) are obtained after the execution of the respective schemes (a,b). A selective on-resonance refocusing pulse centered at the middle of the C2 carbon resonances (∼ 50 ppm) is used during the IP-IP and AP-AP schemes (a and d, respectively). A shaped refocusing on/off-resonance pulse centered at the middle of both the C1 (∼ 100 ppm) and nitrogen bearing C2 resonances (∼ 50 ppm) is used in the IP-AP scheme (b). For practical reasons, the on/off-resonance pulse required for the refocusing of the C2 and C3 resonances during the AP-IP scheme (c) can be set at the center of the hexose and hexosamine ring carbon resonances (∼ 62 ppm). Finally, a linear combination of the spectra (e–h) is used to obtain the virtual decoupled spectrum (i).

In the following, we exemplify in some detail how ^13^C isotope labeling can be made highly useful in the structural analysis of glycans by ^13^C,^13^C-TOCSY NMR experiments. The use of in-phase antiphase (IPAP) or double in-phase antiphase (DIPAP) schemes can be applied to obtain virtually decoupled ^13^C,^13^C-TOCSY spectra of ^13^C-uniformly labeled carbohydrates. These spectra can be recorded in a 2D manner, using the pulse sequences described previously by Richter et al.^343^ for the study of ^13^C-enriched RNA samples. The IPAP scheme (Figure 25a,b) can be employed to virtually decouple homonuclear ^1^*J*_C1,C2_ scalar couplings of anomeric carbon resonances in the direct dimension. In this case, two selective on-resonance pulses (90° excitation and 180° refocusing) are applied at the center of the anomeric carbon resonances (C1 ∼ 100 ppm), whereas two selective 180° off-resonance refocusing pulses are applied in a region of the spectrum that comprises the C2 resonances; for practical reasons, the off-resonance pulse can be set at the center of the hexose and hexosamine ring carbon resonances (∼ 62 ppm). This scheme includes a constant time delay *T* = 1/4·^1^*J*_CC_. Because the experiment is recorded in an interleaved manner, the in-phase and antiphase spectra need to be split and processed separately (Figure 25c,d, respectively). The combined spectra (Figure 25e,f) are then shifted (downfield and upfield, respectively) by 0.5·^1^*J*_CC_ to obtain the correct chemical shift (Figure 25g,h); once the latter spectra are combined, the virtually decoupled spectrum is obtained (Figure 25i and Figure 26 bottom). ^1^H- and ^15^N-decoupling (in the case of ^15^N-enriched carbohydrates) can be performed using state-of-the-art decoupling schemes (i.e., WALTZ65 and GARP4, respectively). The DIPAP scheme can be useful to observe ^13^C,^13^C-TOCSY correlations from nitrogen-bearing C2 carbons (∼ 50 ppm) with simultaneous virtual decoupling of the ^1^*J*_C1,C2_ and ^1^*J*_C2,C3_ couplings (Figure 27). In this case, three different 180° selective carbon refocusing pulses are employed in the scheme: an on-resonance pulse centered at the middle of the C2 carbon resonances (∼ 50 ppm, Figure 27a,d), an on/off-resonance pulse centered both at the middle of the C1 (∼ 100 ppm) and nitrogen bearing C2 carbon (∼ 50 ppm) resonances (Figure 27b), and an on/off-resonance pulse centered at the middle of the C2 and C3 resonances (Figure 27c); for practical reasons, the latter can be set at the center of the hexose and hexosamine ring carbon resonances (∼ 62 ppm). In this case, four different subspectra are obtained (Figure 27e–h); in an analogous manner to what was described above, after combining the spectra and shifting the resonances by 0.5·^1^*J*_CC_, the virtual decoupled spectrum is obtained (Figure 27i and Figure 26 top).

**Figure 28:**
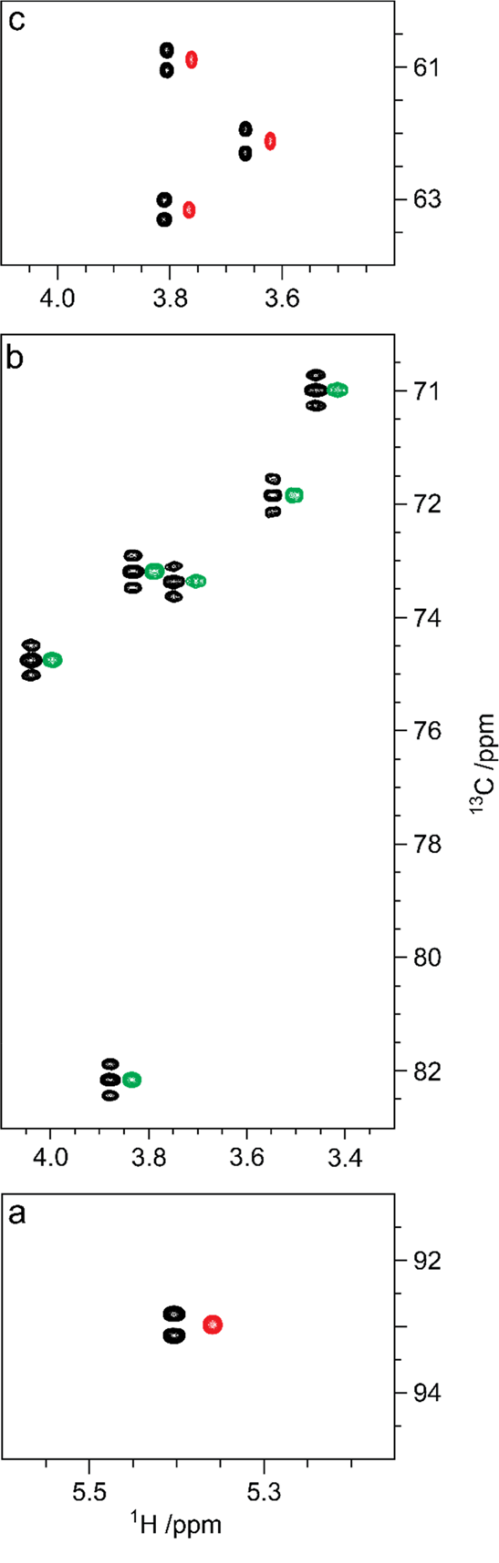
Overlay of the ^1^H,^13^C-HSQC (black color) and the ^1^H,^13^C-CT-HSQC spectra (green and red color) of [UL-^13^C]-sucrose, showing the anomeric and ring atoms regions, as well as that of the hydroxymethyl groups (a–c, respectively). The latter spectrum was recorded with a constant time delay (2*T*) of 22 ms and the ^1^H chemical shifts are displaced by −0.045 ppm for clarity; the sign of the cross-peaks are opposite for carbons directly attached to an odd versus an even number of neighboring non-carbonyl carbons (shown in red and green color, respectively).

With the ^13^C NMR chemical shifts and spin-systems identified, the assignment of ^1^H resonances can subsequently be performed by analysis of chemical shift correlations in ^1^H,^13^C-HSQC NMR spectra. However, due to evolution of ^1^*J*_CC_ couplings, the cross-peaks will be split into multiples along the *F*_1_-dimension, which may be alleviated by performing a constant-time version of the experiment referred to as ^1^H,^13^C-CT-HSQC (Figure 28). Interestingly, and in contrast to multiplicity-edited ^1^H,^13^C-HSQC spectra of glycans at natural isotope abundance in which the cross-peaks of the methylene carbons have opposite phase that makes it possible to easily distinguish these correlations from those of methyl and methine carbons, for the ^1^H,^13^C-CT-HSQC spectra of uniformly ^13^C-labeled glycans the differentiation can be made based on the number of neighboring non-carbonyl carbons, e.g., C1 and C6 in glucose and fructose residues of [UL-^13^C_12_]sucrose, if a refocusing delay corresponding to 1/^1^*J*_CC_ (2*T* = 22 ms) is used in the experiment (Figure 28). Thus, not only are the resonances from the hydroxymethyl groups identified and differentiated but also the anomeric carbon atom of glucose in sucrose. ^13^C uniform labeling, as well as site-specific ^13^C labeling, was used in analysis of N-linked glycans released from glycoproteins.^344,345^ The oligosaccharides analyzed were of the high-mannose type and 2-aminopyridine labeled at the reducing end, one of them being an undecasaccharide referred to as M9 (nine d-mannose and two *N*-acetyl-d-glucosamine residues) and the other a decasaccharide denoted M8B devoid of one of the mannosyl residues. Besides 2D NMR experiments used for ^1^H and ^13^C resonance assignments 3D ^13^C-edited NOESY experiments with a mixing time of 200 ms were acquired from which identification of the presence (or absence) of ^1^H,^1^H-connectivities at specific ^13^C NMR chemical shifts could be made for the M8B and M9 oligosaccharides.

**Figure 29:**
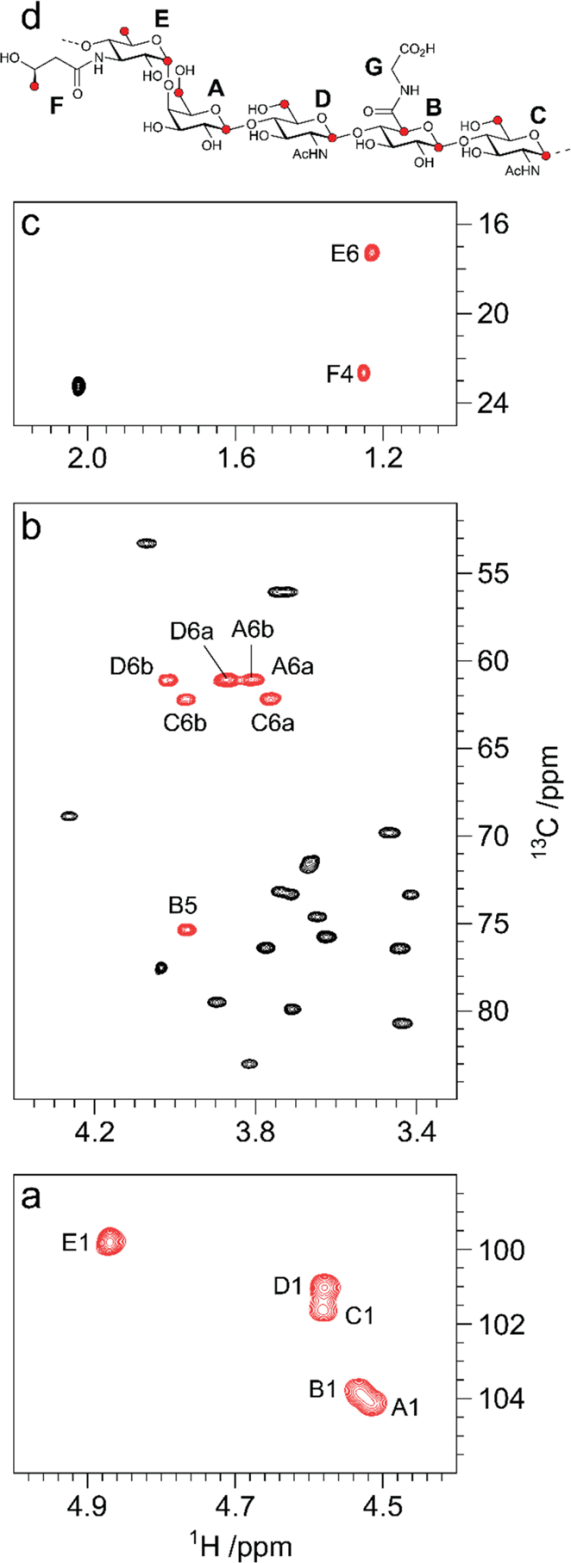
^1^H,^13^C-CT-HSQC spectra (2*T* = 22 ms) of the ^13^C-enriched O-antigen polysaccharide from *E. coli* O91 showing the anomeric region (a), the region for the ring atoms and those from the hydroxymethyl groups (b), and the region of the methyl groups (c). Representation of the structure of the aforementioned polysaccharide in schematic representation (d), where the carbon atoms directly attached to an odd number of neighboring non-carbonyl carbons are indicated with red dots; in the ^1^H,^13^C-CT-HSQC spectrum, the cross-peaks from these atoms have an opposite sign that those from carbons directly attached to an even number of neighboring non-carbonyl carbons (shown in red and black, respectively in a–c).

Detection of α-(2→8)polysialic acid homopolymers on cells in a relatively short experimental time, ∼ 20 min for recording a ^1^H,^13^C-HSQC NMR spectrum, was made possible by ^13^C,^15^N-isotope enrichment.^346^ Notably, the capsular polysaccharide was produced by addition of Neu5Ac (labeled or unlabeled) to the culture medium. NMR spectra of α-(2→8)polysialic acid polymers and the corresponding cell-associated polysaccharides were closely similar, although the ^13^C line widths of the latter were 2–3 times larger. Distinction of ^13^C-neighbors in highly or uniformly ^13^C-labeled glycans from analysis of ^1^H,^13^C-CT-HSQC NMR spectra becomes very informative for polysaccharides that have different types of sugars as well as substituents as shown for the ^13^C-labeled O-antigen polysaccharide from *E. coli* O91 (Figure 29). The O-antigen from *E. coli* O142 was ^13^C- and/or ^15^N-isotope labeled, and NMR studies of this polysaccharide employed, inter alia, TROSY-based ^1^H,^15^N-HSQC and HNCO experiments on ^13^C,^15^N-isotopically labeled material for resonance assignments.^177^ Temperature dependence of coupling constants of the amino sugars in the polysaccharide was investigated from a series of *F*_1_-coupled ^1^H,^15^N-HSQC spectra.

#### 5.1.9 Glycopeptides and Glycoproteins

**Figure 30:**
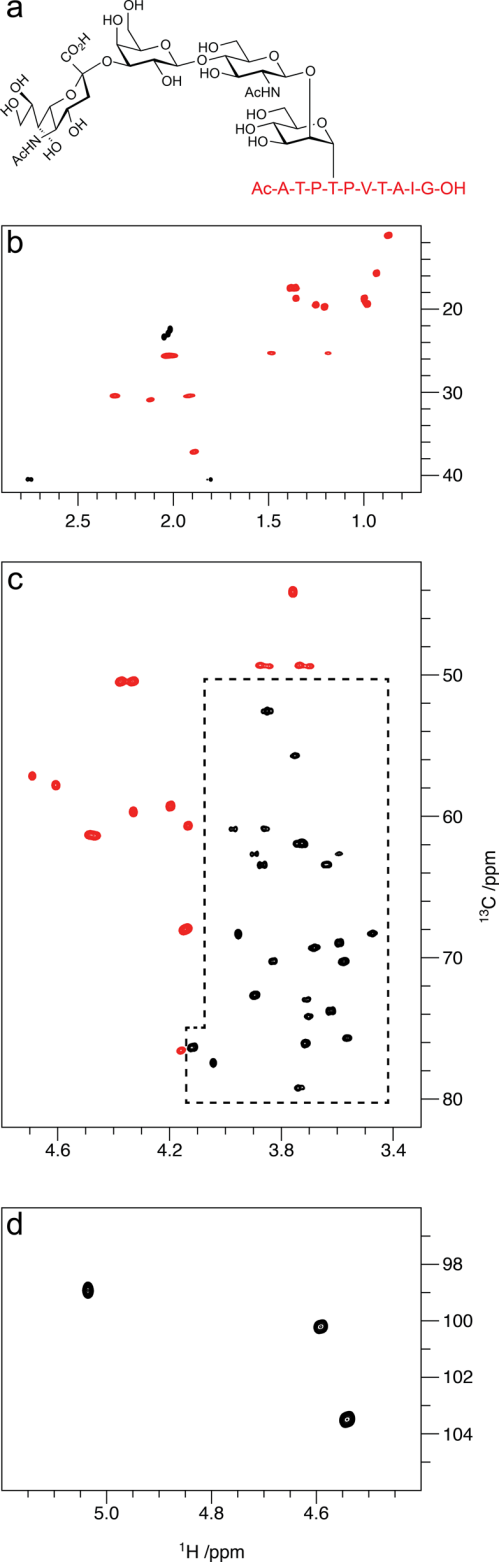
(a) Structure of the tetrasaccharide–decapeptide reported by Šardzík et al.^348^ (b–d) Selected regions of the ^1^H,^13^C-HSQC spectrum (700 MHz), where the correlations from the carbohydrate and peptide moieties are indicated in black and red color, respectively. (b) The region for the side-chain protons of amino acids, H3 of sialic acid and acetyl methyl groups. (c) The region for the ring atoms and hydroxymethyl groups of carbohydrates (highlighted with a dashed line) and that for α-protons of amino acids; (d) the anomeric region.

In glycosylated peptides and proteins, the sugar residue forming the glycosyl–amino acid connectivity is in many cases linked to asparagine in N-linked structures or to serine/threonine in O-linked structures, although other amino acids can be the aglycone, and a good number of different monosaccharide–amino acid combinations have been reported.^115^ High-resolution one-dimensional ^1^H NMR spectroscopy can shed light on, e.g., the structure of O-linked glycans from glycoproteins by relying the concept of structural-reporter-group resonances that are characteristic for specific structural elements, because besides well-resolved resonances from anomeric protons also those from protons of the sugar ring may be utilized, if they are shifted from the bulk of the signals due to glycosylation or substituent effects.^347^ Importantly, even though overlap between sugar and amino acid resonances can occur in NMR spectra depending on the specific sugar and amino acid in the oligo/polysaccharide and glycopeptide/glycoprotein, respectively, the resonances can be found in different regions in ^1^H,^13^C-HSQC NMR spectra (Figure 30).

**Figure 31:**
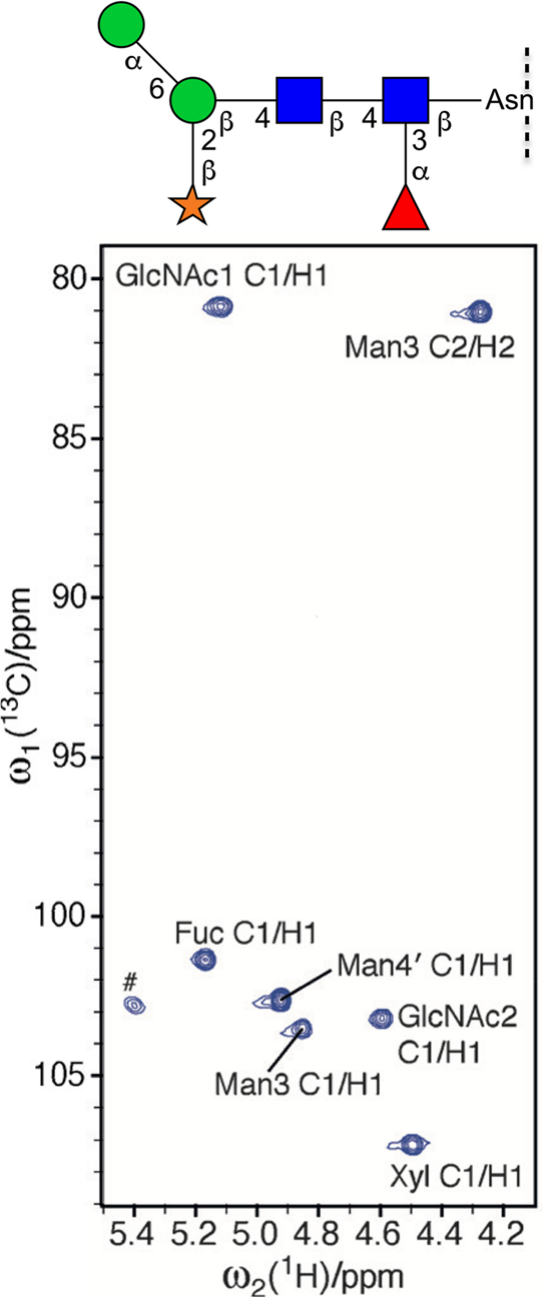
Glycosylation detected by a ^1^H,^13^C-HSQC NMR spectrum in the denatured plant protein bromelain. The spectral region covers cross-peaks from the anomeric resonances of the sugar residues in the N-linked hexasaccharide, shown by SNFG representation. Note that the ^13^C NMR chemical shift of the proximal GlcNAc residue linked to Asn resonates at ∼ 81 ppm, whereas the other sugar residues have their ^13^C chemical shifts for anomeric carbons in the range 101–108 ppm. Adapted and reproduced with permission from ref (349). Copyright 2015 Wiley.

NMR analysis of glycan post-translational modifications of proteins at natural abundance carried out under denaturing conditions, using 7 M urea in D_2_O, eliminates molecular mass restrictions for the proteins.^349^ Deviations from the random-coil NMR chemical shifts for the amino acids in the protein indicate that modifications have taken place. The anomeric region of a ^1^H,^13^C-HSQC NMR spectrum (Figure 31) is of particular interest, as these cross-peaks give information on glycan structure and complemented with ^1^H,^1^H-TOCSY spectra a good deal of information can be obtained on constituent sugar residues. Analysis of glycosylation patterns in monoclonal antibody therapeutics by NMR spectroscopy employed denaturing conditions and focused on the fingerprint characteristics of anomeric region in purified Fc domains from digested mAb molecules in order to profile and differentiate glycan composition.^350,351^ An alternative approach in analysis of intact glycoproteins at natural isotope abundance is to rely on the differences in nuclear spin relaxation of the NMR signals from the protein and the glycan(s).^352^ The analysis was performed on two glycoforms of RNase B containing high-mannose M5 and M9 N-linked glycan variants. Based on line widths, the ^13^C transverse relaxation times of the glycans were ∼ 25% longer than those from the protein resonances. The corresponding difference for the ^1^H *T*_2_ values were ∼ 80%, i.e., almost 2-fold, and this information was used to select a mixing time of 90 ms in ^1^H,^13^C-HSQC-TOCSY experiments. The resulting 2D NMR spectra were essentially devoid of signals from the protein, thus mainly showing cross-peaks from glycans.

The high complexity of glycoprotein NMR spectra may be alleviated by stable isotope labeling, which in addition increases the sensitivity when ^13^C- and/or ^15^N-labeling is utilized. Several labeling schemes have been devised, including uniform labeling, segmental labeling of either the glycan or the protein, and residue specific labeling^353^ performed by, inter alia, metabolic labeling or in vitro labeling.^354,355^ The segmental labeling approach was chosen in a study of an N-linked glycoprotein from *Campylobacter jejuni*, in which the protein was uniformly ^13^C,^15^N-labeled, whereas the glycan heptasaccharide at natural isotope abundance was in vitro glycosylated.^356^ This labeling scheme enabled ^15^N-filtered-filtered ^1^H,^1^H-NOESY and ^13^C-filtered-filtered ^1^H,^1^H-NOESY experiments to be carried out, whereby all protein signals are suppressed and only the resonances from the unlabeled glycan are observed. The use of these 2D filtered/edited NOESY experiments^357^ and 3D ^13^C-filtered-edited ^1^H,^1^H-NOESY experiments facilitated identification of a large number of NOEs used for structural characterization of the glycoprotein.

**Figure 32:**
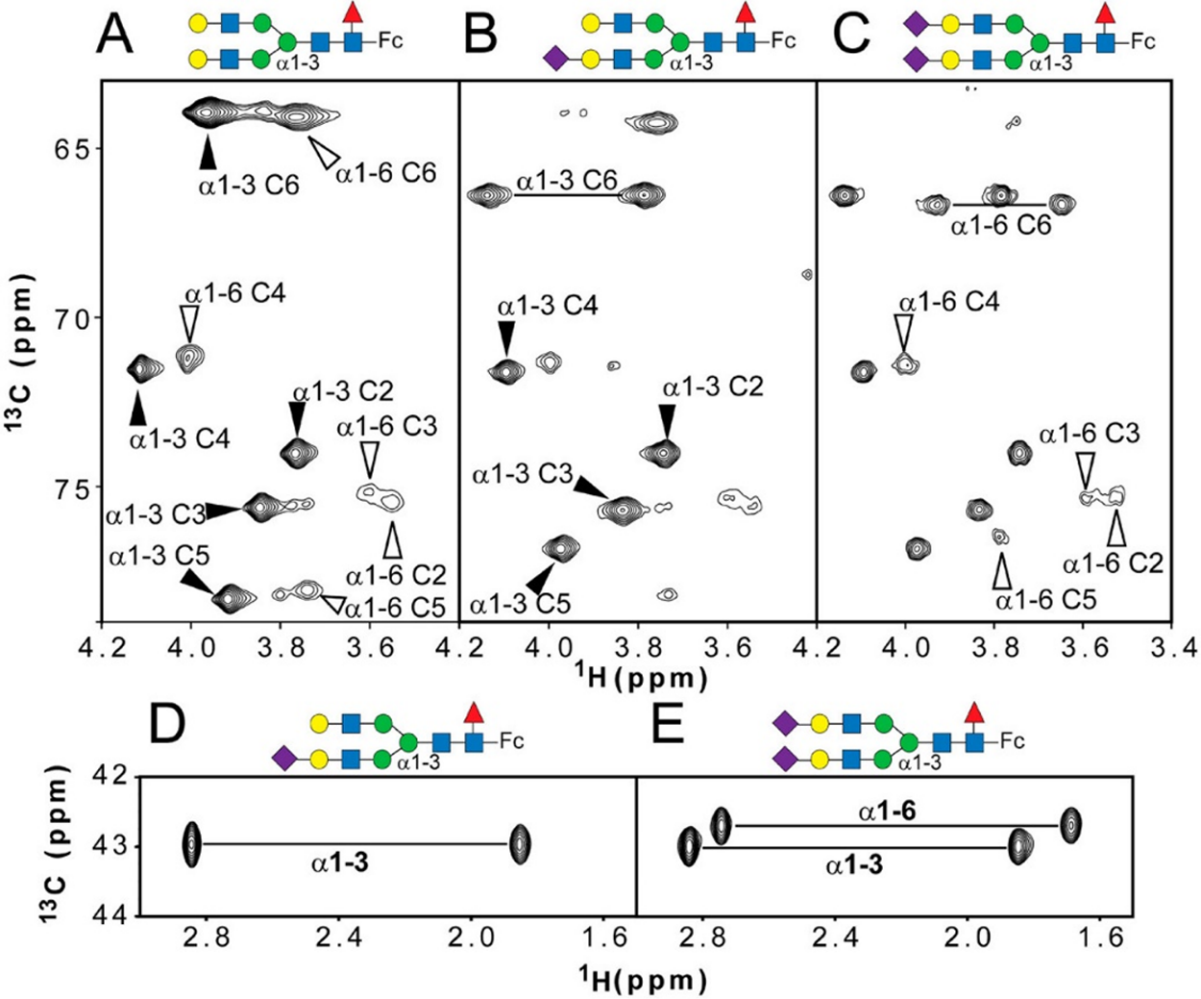
NMR spectra of terminal Gal and/or terminal *N*-acetylneuraminic acid residues of Fc-conjugated N-glycan show distinct ^1^H,^13^C-correlations. (A) [UL-^13^C_6_]Gal resonances observed in a ^1^H,^13^C-HSQC spectrum of Gal-terminated Fc. (B) A corresponding spectrum in which the Fc has an *N*-acetyl-[1,2,3-^13^C_3_]neuraminic acid residue attached to the Gal residue of the α-(1→3)-Man branch in the N-glycan structure. (C) ^1^H,^13^C-HSQC spectrum of glycosylated Fc domain in which both branches of the N-glycan have been isotopically labeled with [UL-^13^C_6_]Gal and *N*-acetyl-[1,2,3-^13^C_3_]neuraminic acid. (D,E) ^1^H,^13^C-HSQC spectra of the region for C3–H3 correlations from terminal *N*-acetylneuraminic acid residues of the α-(1→3/6)-Man branches. Reproduced with permission from ref (361). Copyright 2012 American Chemical Society.

Enzymatic glycan remodeling was carried to label the sialic acid-containing N-glycan of the α-2,6-sialyltransferase (ST6Gal-I) with site specifically ^13^C-labeled Neu5Ac.^358^ After neuraminidase treatment and removal of the terminal sialic acids, the enzyme was resialylated using CMP-β-[1,2,3,10,11-^13^C_5_]Neu5Ac. The α-2,6-sialyltransferase now containing terminal ^13^C-labeled Neu5Ac residue(s) as part of its N-glycan showed only one set of cross-peaks at δ_C3_ 48.8 to δ_H3ax_ 1.72 and δ_H3eq_ 2.68 in the ^1^H,^13^C-HSQC NMR spectrum, but from a 3D ^1^H–^13^C–^13^C correlated NMR experiment, it was possible to show the presence of two different sets of C2–H3_ax_ and C2–H3_eq_ correlations in the plane, corresponding to the ^13^C chemical shift of C3 in the sialic acids. In another study, the Fc fragment of an immunoglobulin G was remodeled using ST6Gal-I, and the terminal sialic acid was introduced in a specific manner preferentially on the α-(1→3)-branch.^359^ Subsequent developments introduced d-[UL-^13^C_6_]galactose terminally at N-glycans of IgG1 and its Fab fragment; NMR chemical shift as well as intensity differences were identified in ^1^H,^13^C-HMQC spectra.^360^ Furthermore, Fc fragments were remodeled to include uniformly ^13^C-labeled galactose residues at both the 3- and 6-branches of the N-glycan. Additionally, also d-[1,2-^13^C_2_]galactose labeling was performed in a corresponding way, thereby identifying resonances from C2 atoms. This labeling scheme also made it possible to better examine the ^1^H resonance line widths from the two different galactosyl residues. By relying on the fact that ST6Gal-I preferentially sialylates galactose on the α-(1→3)-branch, it was feasible to remodel the glycan to have a terminal d-[UL-^13^C_6_]galactose specifically on the α-(1→6)-branch. Taken together, it was then possible to deduce that a sharper set of peaks originate from the galactosyl residue on the α-(1→3)-branch and that broad peaks emanate from the galactosyl residue on the α-(1→6)-branch. The various substitution patterns and ^13^C-labeling approaches are well illustrated by the different combinations and cross-peak regions in ^1^H,^13^C-HSQC NMR spectra (Figure 32).^361^

**Figure 33:**
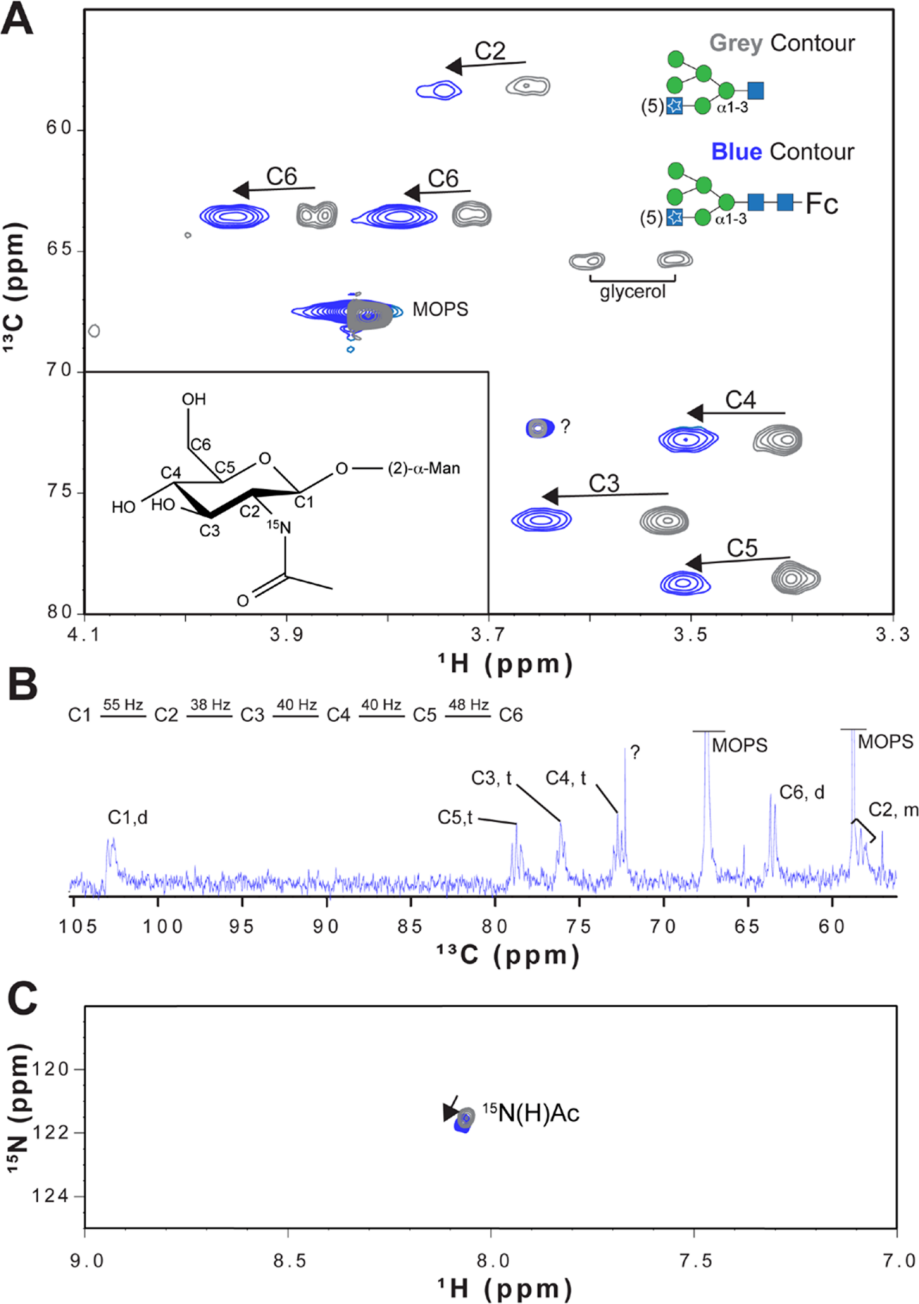
^1^H,^13^C-HSQC NMR spectra of IgG1 F_c_ with a Man5 N-glycan following addition of [^13^C,^15^N]GlcNAc, denoted by *N in the glycan name and shown as a blue square with a white star in the SNFG representation. (A) A 2D ^1^H,^13^C-HSQC spectrum of the *N-Man5 N-glycan following EndoF1-catalyzed hydrolysis is shown as gray contours. Blue contours show the positions of peaks from IgG1 Fc bearing a *N-Man5 N-glycan. ^1^*J*_CC_ couplings are not resolved because of the limited resolution in the ^13^C dimension. (B) 1D ^13^C-observe NMR spectrum of *N-Man5 Fc with ^1^*J*_CC_ values indicated. (C) 2D ^1^H,^15^N-HSQC spectra before and after N-glycan hydrolysis with the same colors used in (A). Reproduced with permission from ref (362). Copyright 2015 American Chemical Society.

The complex N-glycan structures typically have *N*-acetyl-d-glucosamine residues in both branches, where they are β-(1→2)-linked to the mannosyl residues at the branching region. Starting from an M5 N-glycan structure on an Fc fragment from an IgG1, enzymatic remodeling using UDP-[^13^C,^15^N]GlcNAc (where the isotope labeling was obtained from [UL-^13^C_6_]Glc and [^15^N-*amido*]glutamine) and the glycosyltransferase Gnt1 the isotope labeled d-GlcNAc residue could be added to the α-(1→3)-branch of the N-glycan on the Fc fragment.^362^ In comparison to the released N-glycan, ^1^H NMR chemical shift displacements occur for the *N*-acetyl-d-glucosamine resonances of the Fc-linked form as a result of interactions with the protein (Figure 33). Pruning of the *N*-glycan on the Fc fragment down to an M3F structure facilitated installation of isotope labeled d-GlcNAc residues on both branches of the N-glycan, employing first Gnt1 as described and subsequently Gnt2 in conjunction with UDP-[^13^C,^15^N]GlcNAc, resulting in an *N*-acetyl-d-glucosamine residue also at the α-(1→6)-branch. A suite of 2D NMR experiments was developed to correlate H3–C2, H3–C1, and C1–C2 nuclei in terminal [1,2,3-^13^C_3_]Neu5Ac isotope labeled *N*-glycan on the 55 kDa IgG1 Fc domain.^363^ The labeling scheme was also applied to α-(2→8)polysialic acid polymers, and the three 2D NMR experiments were as suitable for purified polysaccharides as for the corresponding cell-associated polymers.

**Figure 34:**
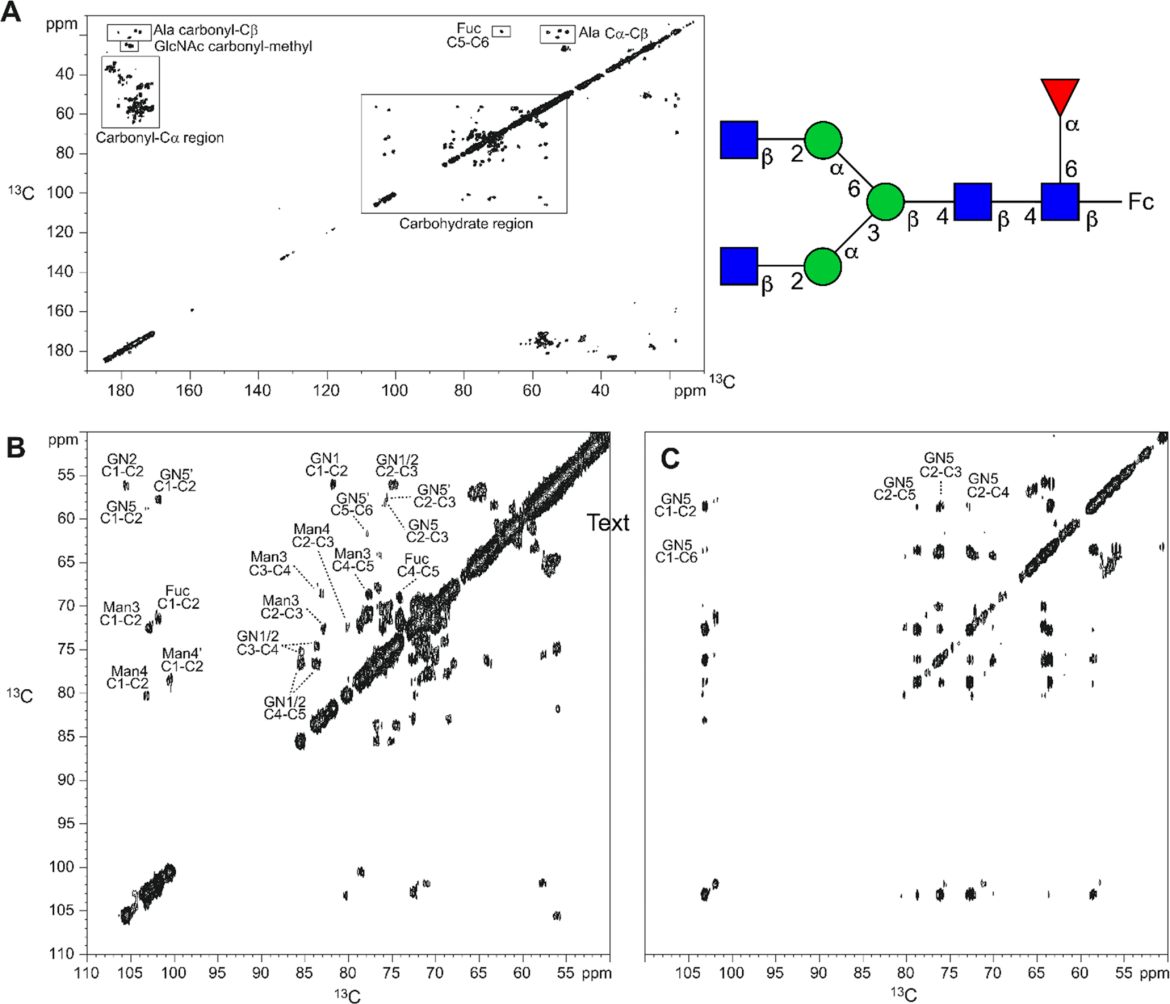
Full spectral region (A) and oligosaccharide region (B) of the 2D ^13^C,^13^C-NOESY spectrum of ^13^C-labeled IgG-Fc acquired at 125 MHz with a mixing time of 600 ms and (C) ^13^C,^13^C-TOCSY spectrum, in which the magnetization transfer was performed with the FLOPSY pulse sequence with a mixing time of 1.2 s. Adapted and reproduced with permission from ref (364). Copyright 2009 Elsevier.

**Figure 35:**
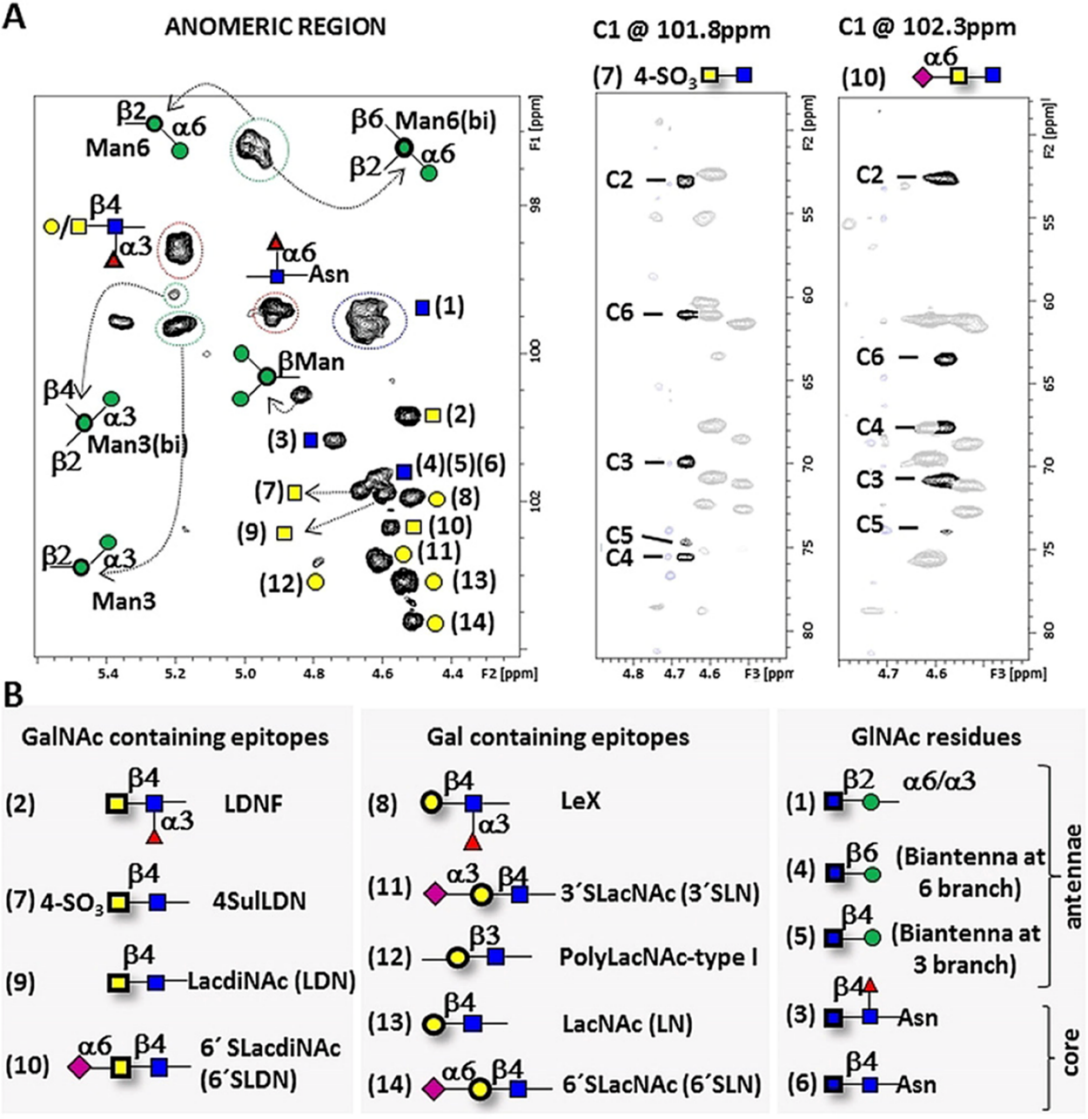
NMR identification of glycan structures on SARS-CoV-2 receptor binding domain (RBD) glycoprotein. (A) Anomeric region of the ^1^H,^13^C-HSQC spectrum of RBD (left); selected planes for C1 GalNAc on the 4SulLDN fragment and for C1 GalNAc on 6′SLDN from an edited 3D HCCH-TOCSY spectrum showing the correlations to all ^13^C atoms within the pyranose spin system (right). (B) GalNAc, Gal and GlcNAc containing epitopes in N-linked glycans on RBD. Reproduced with permission from ref (366). Copyright 2020 The Authors.

Uniform ^13^C-isotope labeling of a mouse monoclonal IgG2b antibody was carried out using d-[UL-^13^C_6_]Glc, and after cleavage by protease digestion and purification, a 56 kDa Fc fragment was isolated containing an octasaccharide N-linked glycan.^364^ At a ^13^C NMR frequency of 125 MHz, a mixing time of 600 ms was chosen for detecting one-bond correlations in the ^13^C,^13^C-NOESY spectrum (Figure 34) as longer mixing times gave fewer cross-peaks. Variation in relative intensities of the C1,C2 cross-peaks were proposed to be due to different local mobilities of the sugar residues. Additionally, a ^13^C,^13^C-TOCSY NMR experiment was carried out with a mixing time of 1200 ms, and whereas the *N*-acetyl-d-glucosamine residue on the α-(1→3)-branch exhibited extensive intraresidue C1–C5 correlations, the corresponding ones for the d-GlcNAc residue on the α-(1→6)-branch were barely observed. In glycoprofile analysis of an intact uniformly ^13^C,^15^N-labeled glycoprotein from an IgE high-affinity receptor, the anomeric region of a ^1^H,^13^C-HSQC spectrum was analyzed to map the constituent glycoforms of the N-linked glycans.^365^ Interestingly, the H1,C1 cross-peak of the proximal d-GlcNAc residue linked to asparagine in the protein was not detected in the native folded state. However, using denaturing conditions, it could readily be observed, highlighting the complementarity of the latter approach in analysis of glycoproteins. Uniform ^13^C-labeling was used to study the glycans at two N-glycosylations sites in the domain B of subunit S1 from the receptor binding domain (RBD) of SARS-CoV-2.^366^ Like for other glycoproteins, the anomeric region in the ^1^H,^13^C-HSQC spectrum was essential in identification of glycan structures present (Figure 35). In addition, a type of edited 3D HCCH-TOCSY experiment^367,368^ could unravel the complete C1–C6 spin-systems of d-GalNAc residues in the N-glycans (note that in d-GalNAc the ^3^*J*_H4,H5_ coupling constant is small and limits ^1^H,^1^H-TOCSY transfer, which is alleviated by using ^13^C,^13^C-TOCSY transfer in the experiment).

Sparse isotope labeling of glycoproteins using d-[UL-^13^C_6_]glucose has been shown to be an alternative approach, as standard commercial growth media can be complemented by an equal amount of isotope labeled glucose to that present in the growth medium.^369^ This was exemplified for a ∼ 12 kDa protein that has high levels of Man_5_GlcNAc_2_ N-glycan structures at its three N-glycosylation sites. Theoretically, half of the sugars should be ^13^C-enriched; experimentally this was observed to be ∼ 40%, also for the *N*-acetyl groups of the glucosamine residues. Alanine methyl groups were labeled to a decent level of ∼ 20%. The study explored both ^1^H,^13^C-HSQC experiments, where the ^1^*J*_CC_ couplings in the *F*_1_-dimension still evolve, and for the *N*-acetyl methyl groups, this resulted in a doublet with a peak separation of ∼ 50 Hz. In the ^1^H,^13^C-CT-HSQC experiment, the one-bond couplings in the *F*_1_-dimension are refocused, but this experiment causes loss of sensitivity, in particular, for resonances broadened by lack of internal motion.

### 5.2 Correlations between Sugar Residues

#### 5.2.1 HMBC and NOESY NMR Experiments

To obtain sequence information between sugar residues, the ^1^H,^1^H-NOESY NMR experiment is useful for polysaccharides, whereas ^1^H,^1^H-ROESY or ^1^H,^1^H-T-ROESY experiments are the experiments of choice for oligosaccharides with a few sugar residues. Detection of dipolar interactions of protons close in space, and as such not only protons within the same residue but also between sugar residues, then facilitates sequential information to be obtained. Thus, for proximate protons at the glycosidic linkage, observed NOEs will reveal information on sequence between sugar residues. However, for some stereochemical arrangements, the NOE between the anomeric proton in one residue and the proton on the glycosyloxylated carbon atom in the subsequent sugar residue may not be the pair of protons closest in space, which may instead be a proton vicinal to the substitution position, e.g., in the disaccharide structural elements α-d-Fuc*p*-(1→3)-d-Gal*p* and α-l-Fuc*p*-(1→3)-β-d-Man*p*, where the inter-residue distance H1′–H4 and H1′–H2, respectively, is shorter than the transglycosidic distance H1′–H3.^370^ Sequence information can still be deduced, although linkage position may not be determined without a detailed analysis.

The spectral quality of ^1^H,^1^H-NOESY spectra has been shown to be increased significantly by elimination of zero-quantum coherence, as this gives rise to antiphase dispersive components.^371^ The improved methodology to obtain pure absorption line shapes in spectra is based on the simultaneous application of a swept-frequency π pulse and a pulsed-field-gradient during the mixing time of the experiment. After the spin–echo, which initially refocuses the evolution of the zero-quantum coherence, a continued evolution of the zero-quantum coherence takes place, leading to that different parts of the sample have accrued a different phase of the zero-quantum coherence, resulting in its cancellation. Further developments to improve spectral quality from ^1^H,^1^H-NOESY experiments have been based on pure echo^302^ decoupling during the *t*_1_ period of the 2D NMR experiment^372^ and is best performed in conjunction with the zero-quantum coherence suppression technique.

**Figure 36:**
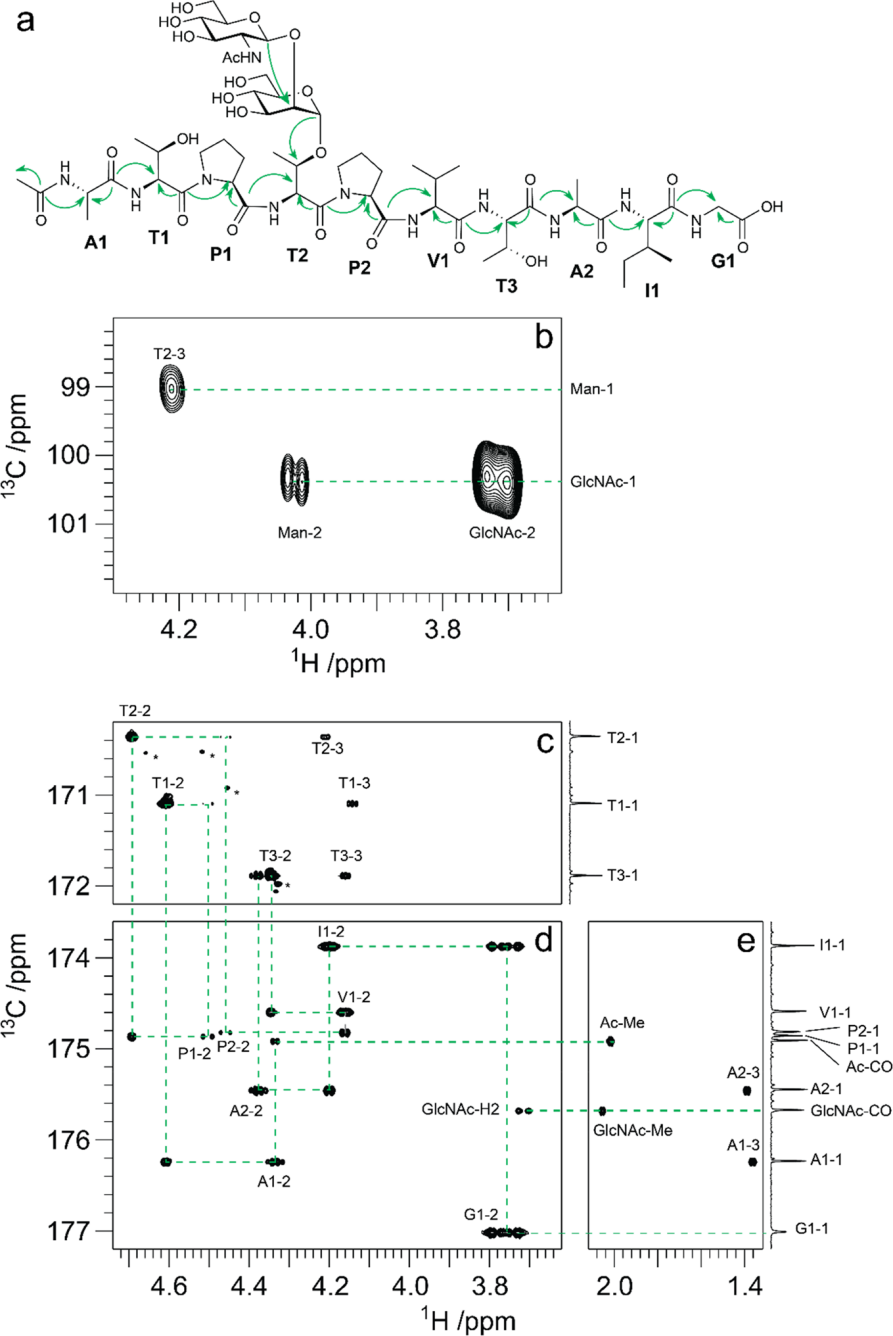
(a) Structure of the disaccharide–decapeptide reported by Šardzík et al.,^348^ showing carbon–proton long-range inter-residue correlations from the ^1^H,^13^C-HMBC and ^1^H,^13^C-BS-CT-HMBC spectra. (b) Selected region of the ^1^H,^13^C-HMBC spectrum showing correlations from anomeric carbons. (c–e) Different regions of the ^1^H,^13^C-BS-CT-HMBC spectrum showing correlations from carbonyl carbons.

The ^1^H,^13^C-HMBC NMR experiment commonly detects two- and three-bond correlations for which ^*n*^*J*_CH_ < 10 Hz, and transglycosidic correlations have ^3^*J*_CH_ in the range 3–6 Hz. Besides revealing sequence information between sugar residues, this experiment can also be used to establish the glycosylation sites of glycopeptides (Figure 36b). Furthermore, the peptide sequential information can readily be obtained in D_2_O solution from a ^1^H,^13^C-BS-CT-HMBC spectrum recorded with a selective excitation pulse centered at the carbonyl carbon resonances, which provides a correlation map in which each carbonyl resonance of the peptide bonds is correlated via ^2^*J*_CH_ and ^3^*J*_CH_ coupling constants to intra- and inter-residual protons, respectively (Figure 36c−e).^348^ This strategy was recently implemented in DMSO-*d*_6_ solution for sequence assignment of cyclic lipoglycopeptides isolated from the cyanobacterium *D. muscorum*.^373^

Further developments from the original HMBC pulse sequence^374^ include BIRD-HMBC, with a two-step low-pass *J* filter (LPJF).^375^ In the experiment, ^1^*J*_CH_ coupled proton signals evolve into pure in-phase coherence and long-range ^n^*J*_CH_ coupled proton signals evolve into pure antiphase coherence for subsequent dephasing and evolution, respectively. By accordion-type spectroscopy^376^ where NMR parameters are synchronously incremented and/or decremented as in the constant-time accordion BIRD-HMBC experiment,^377^ further improvements are possible with a variable long-range delay optimized to cover a range of *J* values in conjunction with the constant time element, by which the modulation, due to ^1^H,^1^H scalar couplings, is suppressed along the *F*_1_ dimension.

Elimination of cross-peaks due to ^1^*J*_CH_ couplings in HMBC spectra may be performed with a third-order LPJF, subsequent to the first ^1^H pulse of the experiment, for which the delays are set according to the range of one-bond couplings to be suppressed. In the presence of strong coupling among protons, which occurs for carbohydrates, one-bond artifacts arise in HMBC spectra and may be a nuisance for the interpretation of long-range correlations. This may be alleviated by adding a third-order LPJF, with two of the steps at the end in a four-step LPJF cycle based on different delays. The experiment was dubbed *clean* HMBC and was shown to decrease one-bond strong coupling artifacts in a sample of d-mannose.^378^ A further development of the *clean* HMBC experiment employed for a third-order LPJF an initial conventional second-order LPJF, whereas for the last LPJF dephasing of magnetization from ^1^H nuclei, one-bond coupled to ^13^C was performed by an adiabatic frequency swept π pulse on ^13^C that inverts the latter at different positions in the sample at different times when carried out in the presence of a pulsed-field-gradient.^379^ Excellent artifact suppression was in this case shown for *clean* HMBC spectra of the trisaccharide raffinose and a mannan polysaccharide. The technique implemented as *clean* HMBC has been applied to complex carbohydrate compounds to purge artifacts due to strong coupling.^203,380^

**Figure 37:**
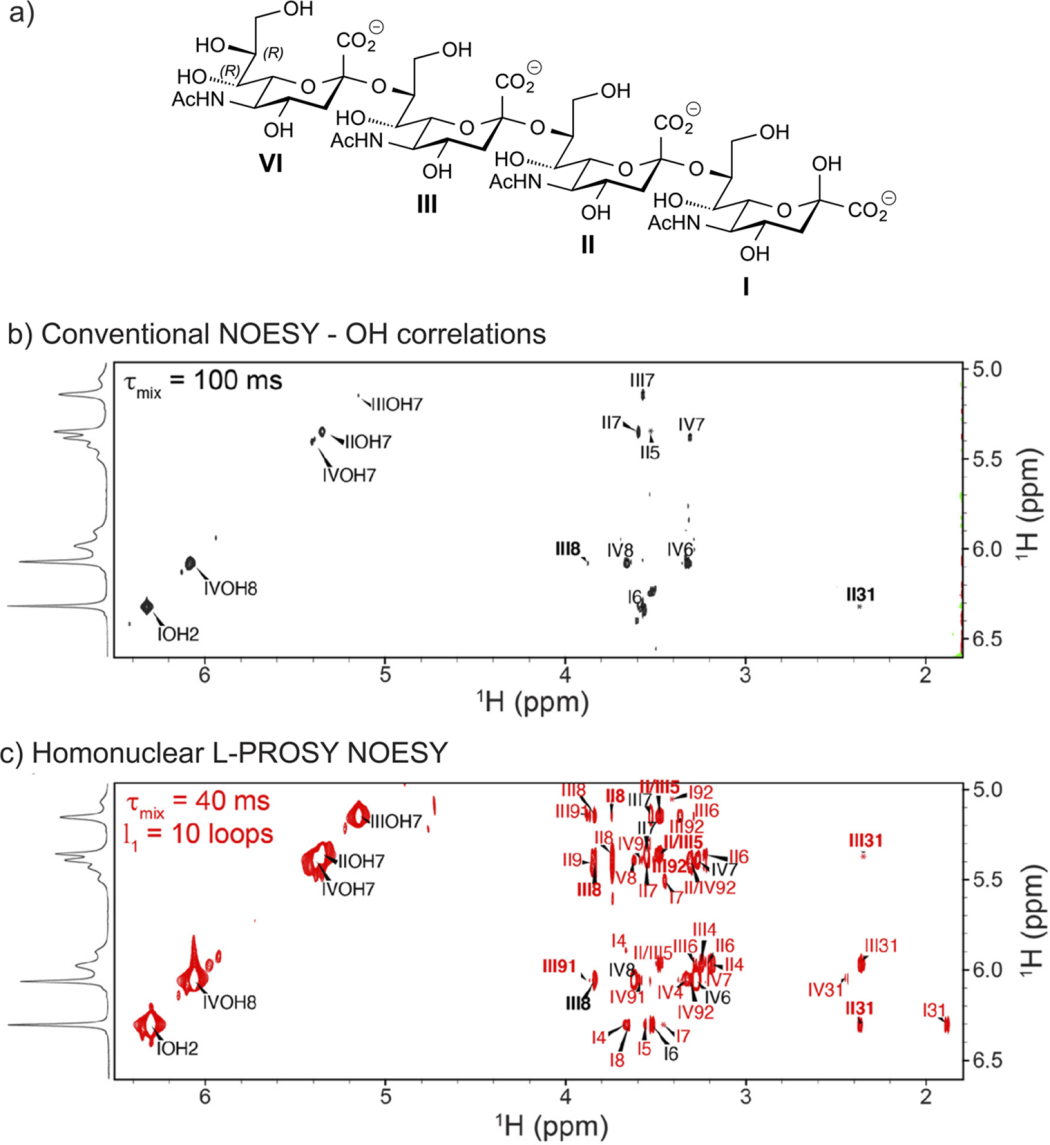
Conventional and L-PROSY NOESY experiments acquired on an *N*-acetylated α-(2→8)-linked sialic acid tetramer (a) at 5 °C and 1 GHz. (b) Hydroxyl group region of a conventional NOESY, optimized with a single mixing time of 100 ms, which is the upper boundary when considering the fast chemical exchange of hydroxyl groups with water; conventional NOESY spectrum shows only short-range cross-peaks of hydroxyl groups. (c) Homonuclear L-PROSY NOESY spectrum acquired under similar conditions, with 10 loops and 40 ms per loop, yielding an average enhancement of ∼ 4.5× over the conventional NOESY as well as the multiple new long-range correlations labeled in red. Placed along the *F*_1_ axes are the hydroxyl proton regions acquired using 1D excitation sculpting. Adapted and reproduced with permission from ref (381). Copyright 2021 American Chemical Society.

In NMR spectroscopy studies of carbohydrate molecules, the protons of the omnipresent hydroxyl groups are to a large extent an untapped source of information, as these are exchanging rapidly with water. Looped projected spectroscopy (L-PROSY) in the form of a ^1^H,^1^H-NOESY NMR experiment alleviates difficulties in utilizing and detecting cross-relaxation peaks in 2D NOESY spectra.^381^ By utilizing frequency selective π/2 pulses bracketing the *t*_1_ evolution time and targeting only the exchangeable hydroxyl protons in a looping scheme carried out several times, this enables significant intensity buildup of cross-peaks (∼ 4.5×) with nonlabile protons in the L-PROSY-NOESY spectrum, as shown for a sialic acid tetrasaccharide (Figure 37). Hydroxyl groups in carbohydrates exchange with water at a rate of 10–10^3^ s^–1^ at room temperature, and in order to estimate the number of loops *n*_loop_ for optimal acquisition parameters, the following relationship is useful: *n*_loop_ × τ_mix_ ≈ *T*_1_(nonlabile), where the optimal mixing time τ_mix_ is dependent on the rate of chemical exchange, which should be fast enough to facilitate adequate repolarization of the labile protons; thus τ_mix_ ≈ (*k*_ex_)^−1^, where *k*_ex_ is the exchange rate of the hydroxyl protons. For the protons of amide groups, the exchange is slower, although significant NOE enhancements (∼ 2.5×) can still be obtained, which enables inter-residue correlations to be observed also for these protons.

#### 5.2.2 DDCCR NMR Experiments

**Figure 38:**
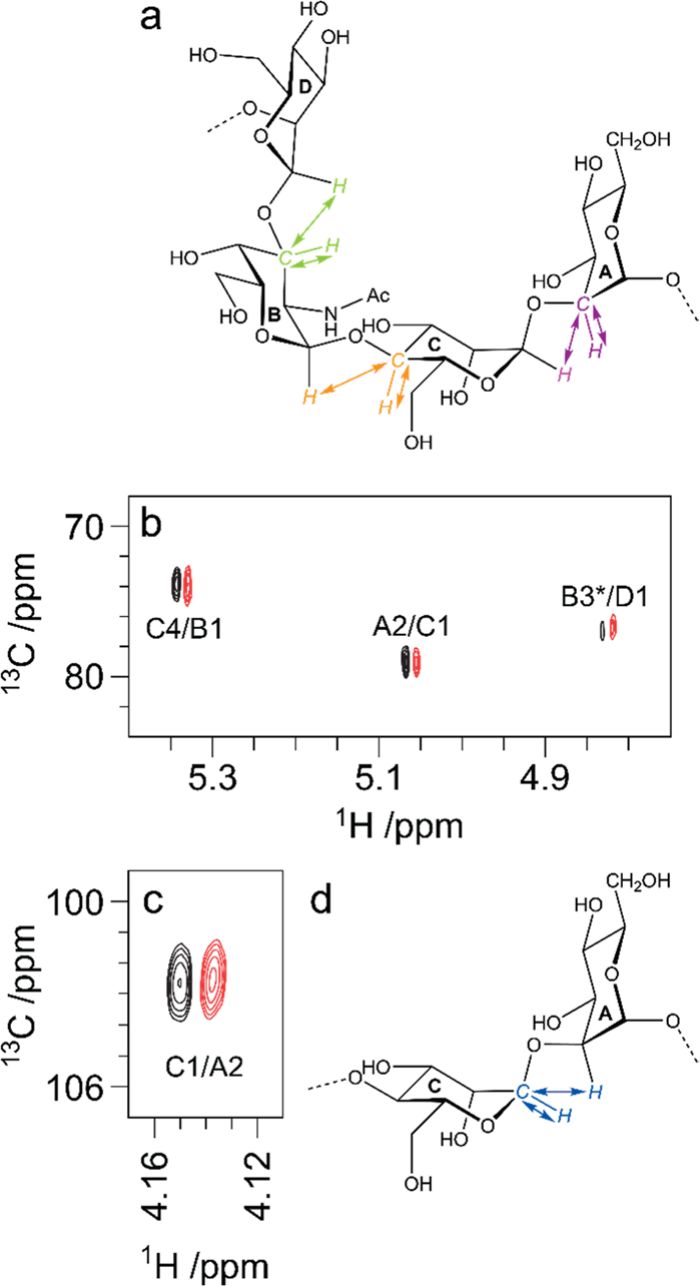
(a) Representation of the structure of the repeating unit, →4)-α-d-Man*p*-(1→2)-α-d-Man*p*-(1→2)-β-d-Man*p*-(1→3)-α-d-Glc*p*NAc(1→ , of the O-antigen polysaccharide of *Escherichia coli* O176, where the dipole pairs whose cross-correlations are observed in the spectrum of (b) are represented in green, orange, and purple colors. Selected regions of the proton–carbon dipole–dipole cross-correlated relaxation spectrum (^1^H,^13^C-DDCCR) recorded with a constant time period (2*T*) of 10 ms, showing correlations from (b) anomeric protons and (c) the anomeric carbon of residue C. (d) Representation of the structure of the →4)-α-d-Man*p*-(1→2)-α-d-Man*p*-(1→ moiety of the aforementioned O-specific polysaccharide, where the two dipoles whose correlation is observed in the spectrum of (c) are shown in blue color. The asterisk indicates a tentative assignment due to spectral overlap.

NMR dipole–dipole cross-correlated relaxation (DDCCR) between nuclei that form a pair of internuclear vectors have been used in conformational studies of proteins and nucleic acids in solution.^382,383^ As the cross-correlation rates depend linearly on the overall rotational correlation time, the methodology should also be beneficial in studies of polysaccharides with high molecular mass such as exopolysaccharides.^384^ Thus, based on the DDCCR principle, an NMR experiment was developed to facilitate analysis of cross-correlated relaxation of polysaccharides at ^13^C natural abundance between two dipoles centered on the same carbon atom in order to investigate the interactions across glycosidic linkages, which will give information on sequential arrangement between sugar residues.^223^^1^H,^13^C-correlations in the 2D NMR spectrum will be decoupled in the *F*_1_-dimension and antiphase with respect to the small long-range proton–carbon scalar couplings along *F*_2_ (Figure 38). Because also scalar one-bond proton–carbon couplings will be present in the *F*_2_-dimension of the spectrum, this can be used to determine the magnitude of ^1^*J*_C1,H1_, which is indicative of the anomeric configuration of hexopyranosides (vide supra).

In cases where the transverse relaxation is fast, a short constant-time period of only 10 ms in the DDCCR experiment has been shown to be sufficient to mediate magnetization transfer, compared to a delay of ∼ 60 ms commonly used for the ^1^H,^13^C-HMBC experiment. Detection of cross-peaks in the spectrum employing the DDCCR experiment is limited by a (3cos^2^θ – 1)/2 term, where θ is the projection angle between the two pairs of C–H vectors such that it vanishes for θ = ±54° and ±126°, whereas in the ^1^H,^13^C-HMBC experiment, the glycosidic torsion angles depend on Karplus-type relationships, where for ϕ ≈ ±90° and ψ ≈ ±90°, the corresponding ^3^*J*_CH_ ≈ 0. In application of the DDCCR experiment to the O-antigen polysaccharide from *E. coli* O126, the sugar residues having the α-*gluco*/*galacto* configuration showed intraresidue C3,H3/C3,H1 and C5,H5/C5,H1 as well as transglycosidic correlations emanating from the glycosyloxylated carbon atom, i.e., C*n*,H*n*/C*n*,H1′ where *n* is the substitution position and H1′ is the anomeric proton at the glycosidic linkage; the ^1^H,^13^C-HMBC experiment based on scalar couplings resulted in the corresponding cross-peaks in the 2D NMR spectrum.^224^ The *E. coli* O176 O-polysaccharide has four sugar residues in its repeating unit (Figure 38a),^385^ and in the DDCCR spectrum, correlations were observed between glycosyloxylated carbons and anomeric protons (Figure 38b) as well as between an anomeric carbon and a proton at the linkage position (Figure 38c). The DDCCR experiment is a good complement or alternative to the HMBC experiment, but a caveat may be warranted because as in using the ^1^H,^1^H-NOESY experiment, the linkage position may be misinterpreted if the conformation at the glycosidic linkage is such that the anomeric proton and the proton at the glycosyloxylated carbon are not proximate in space.

#### 5.2.3 Isotope Labeled Glycans

**Figure 39:**
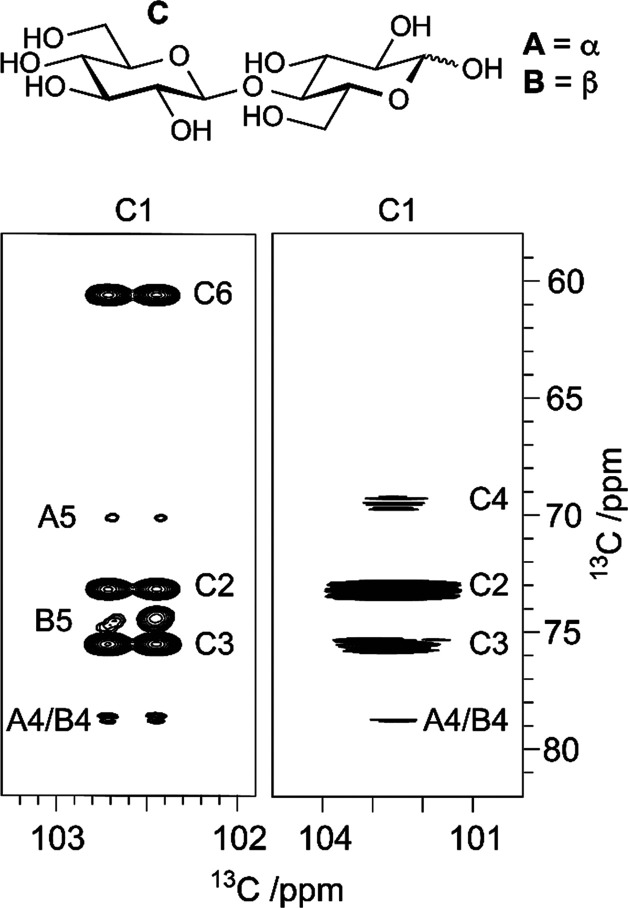
Selected region of the ^13^C,^13^C-CT-COSY (CT = 11.1 ms) and band-selective ^13^C,^13^C-TOCSY (τ_mix_ = 144 ms) of [UL-^13^C_12_]-cellobiose (left and right, respectively), showing intra- and inter-residue correlations from the anomeric carbon of the terminal β-d-glucosyl residue in the disaccharide.

In determination of the sequential arrangement of uniformly ^13^C-labeled sugar residues in oligo- and polysaccharides, the large intraresidue ^1^*J*_CC_ coupling constants should be considered in choosing, implementing, utilizing, and developing NMR experiments. For the [UL-^13^C,^15^N]-labeled α-(2→8)-linked sialic acid tetrasaccharide the ^1^H,^13^C-HSQC-NOESY experiment identified correlations between H7 in one residue and the H3 protons of the contiguous residue.^386^ Even though this type of experiment does not necessarily single out proton pairs at the glycosidic linkage, it can be useful in identifying adjacent sugar residues as was shown for [UL-^13^C_12_]sucrose using a ^1^H,^13^C-CT-HSQC-NOESY experiment (τ_mix_ = 500 ms) showing ^1^H,^1^H-NOE connectivities between H1 in glucose and H1 proton(s) in fructose, detected as an H1g–C1f cross-peak in the 2D NMR spectrum.^148^ Transglycosidic ^*n*^*J*_CC_ coupling constants can be up to ∼ 5 Hz and in a ^13^C,^13^C-CT-COSY experiment on [UL-^13^C_12_]cellobiose correlations from the anomeric carbon C1 of the terminal glucosyl residue via ^2^*J*_CC_ to the A4/B4 carbons of the reducing end glucosyl residue and via ^3^*J*_CC_ to the A5/B5 carbons can be obtained (Figure 39 bottom left), besides intraresidue correlations, in a similar way to what has been observed for [UL-^13^C_12_]sucrose.^148^ Alternatively, a “proton-start” ^13^C,^13^C-TOCSY experiment with a selective spin-lock (τ_mix_ = 144 ms) on the anomeric carbon C1 of the uniformly ^13^C-labeled cellobiose showed transglycosidic correlation(s) to A4/B4 of the reducing end residue (Figure 39 bottom right); the use of a relatively long spin-lock was required because the ^2^*J* magnitude of the C1–A4/B4 correlation is ∼ 2 Hz. The long-range ^1^H,^13^C-CT-HSQC experiment optimized with a significantly larger nominal ^3^*J*_CH_ of 12 Hz than observed across glycosidic linkages revealed a transglycosidic H1g–C2f cross-peak in the heteronuclear 2D NMR spectrum of [UL-^13^C_12_]sucrose.

**Figure 40:**
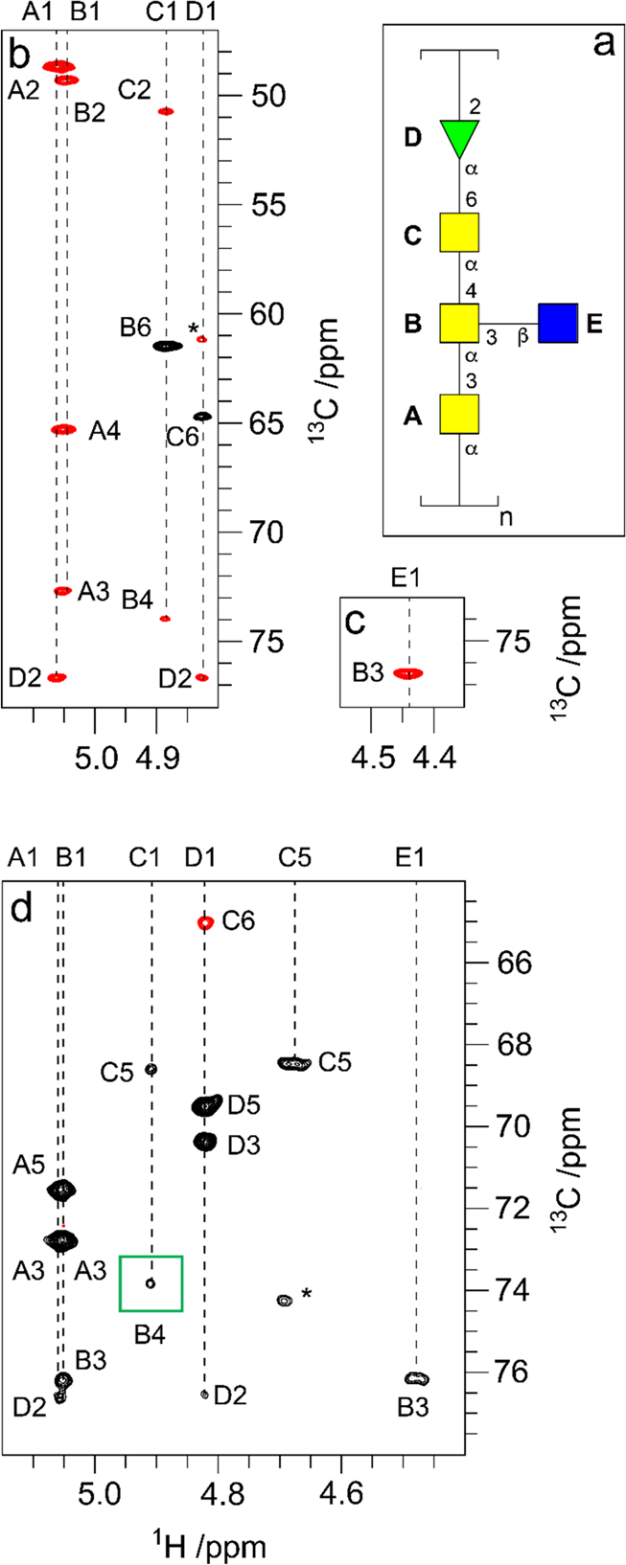
(a) Structure of the O-antigen polysaccharide of *E. coli* O142 in SNFG notation. Selected regions of (b,c) a ^1^H,^13^C-CT-HSQC-NOESY (2*T* = 22 ms, τ_m_ = 100 ms) and (d) a ^1^H,^13^C-LR-CT-HSQC (2*T* = 22 ms, and optimized for ^*n*^*J*_CH_ = 20 Hz) spectra of the ^13^C-enriched O-specific polysaccharide from *E. coli* O142 showing correlations from anomeric protons. The intensity of the cross-peak shown within the green box has been multiplied by a factor of 2. The asterisks denote resonances of minor impurities.

For the elucidation of sequential connectivities in uniformly ^13^C-labeled polysaccharides the ^1^H-detected ^1^H,^13^C-CT-HSQC and ^1^H,^13^C-CT-HSQC-NOESY experiments were the NMR techniques of choice.^148^ The former experiment gives linkage information and a nominal value of ∼ 20 Hz for ^*n*^*J*_CH_ is suitable to define the delay required for the long-range evolution. The latter experiment using a mixing time of ∼ 100 ms identifies spatial proximities between sugar residues, in many cases also defining sequential relationships between sugar residues. However, the determination of the primary sequence in highly or uniformly ^13^C-labeled polysaccharides is best made by acquiring both experiments as exemplified for the ^13^C-enriched O-antigen polysaccharide from *E. coli* O142 (Figure 40).

### 5.3 Miscellaneous

#### 5.3.1 Aliasing and NUS

In order not to cover large spectral widths in indirect dimensions of multidimensional NMR experiments, spectral aliasing or folding may be applied. To improve the spectral resolution in a heteronuclear 2D NMR spectrum, the spectral width in the indirect *F*_1_-dimension is decreased. Signals residing outside of detection in the chosen spectral region will then, depending on whether, e.g., echo/antiecho or states-TPPI quadrature detection is used in *F*_1_, be aliased, resulting in that signals just outside of one end of the spectral window will appear inside the opposite end, whereas if TPPI quadrature detection is used signals are folded, i.e., they are mirrored just inside the edge of the spectrum “close to” the original resonance.^387^ For carbohydrates, the approach may be used to position methyl resonances of 6-deoxy-hexoses or *N*-acetyl groups in ^1^H,^13^C-HSQC spectra or carbonyl resonances in ^1^H,^13^C-HMBC spectra such that they appear in the indirect ^13^C-dimension similar to ring-carbon resonances, although a deconvolution step is required to obtain the true ^13^C NMR chemical shift. The true chemical shift δ_0_ can be obtained from the apparent chemical shift δ_a_ according to δ_0_ = δ_a_ ± *n* × SW_ppm_ where *n* is the unknown aliasing order and SW_ppm_ is the spectral width in ppm. For moderate aliasing where *n* is a low number manual calculation of the true chemical shift works very well, but for small spectral regions in the *F*_1_-dimension computer-aided analysis^388^ is deemed necessary. Application of the technique has been used to resolve ^13^C NMR chemical shifts of glucose in a ^1^H,^13^C-HSQC NMR spectrum. The spectral width in *F*_1_ was reduced to < 1 ppm, and in the aliased spectrum the resonances from the two C4 nuclei of the α- and β-anomeric forms of the monosaccharide were differentiated while being only ∼ 5 Hz apart at 125 MHz.^387^ Similarly, the ^13^C NMR chemical shifts in the trisaccharide melezitose could in an aliased ^1^H,^13^C-HSQC NMR spectrum be resolved by using a small number of increments in the *F*_1_-dimension.^388^

An alternative methodology to increase the spectral resolution in the *F*_1_-dimension of a 2D NMR experiment without increasing the number of data points and consequently the experimental time is to use sparse sampling techniques.^389^ Nonuniform sampling (NUS)^390^ facilitates improved resolution by extending the time in the indirect dimension(s) during which sampling takes place, but without collecting all of the data points in an equal and stepwise manner,^391^ which as a benefit can lead to a 2-fold increase in sensitivity per unit-time of measurement.^392,393^ There are several different ways to sample less data points followed by reconstruction of the FIDs,^394−396^ prior to Fourier transformation to obtain the NMR spectrum. The NUS technique works very well when the sampling density is matched with the envelope of the decaying signal and any modulation caused by, e.g., one-bond ^1^H,^13^C scalar couplings, being on the order of ∼ 150 Hz in carbohydrates. Thus, NUS ^1^H,^13^C-HSQC NMR experiments with a coverage of 10% and 20% have been reported for poly- and oligosaccharides, respectively.^397,398^ However, applying NUS to ^1^H,^1^H-NOESY experiments is significantly more demanding due to the high dynamic range where spectra contain peaks of both high and low intensity.^397,399,400^ Aliasing artifacts in NUS NMR spectra have been investigated and ways to minimize these have been proposed.^401^

#### 5.3.2 NMR Spin Simulations

**Figure 41:**
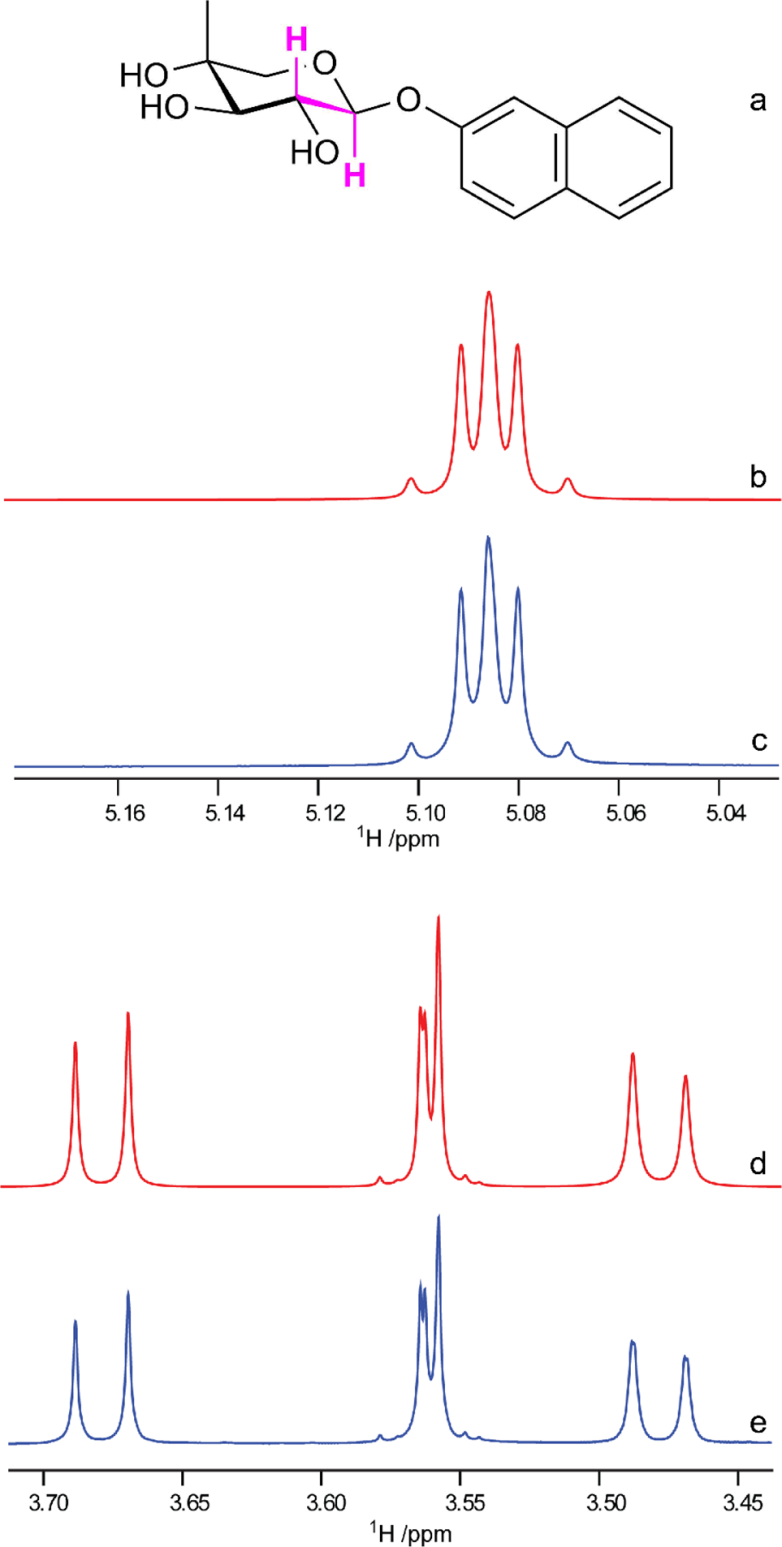
(a) Schematic structure of 2-naphthyl 4-*C*-methyl-β-d-xylopyranoside. Selected regions from the ^1^H NMR spectrum of the monosaccharide glycoside in methanol-*d*_4_ at 37 °C showing the resonance from the anomeric proton using (b) NMR spin simulation (PERCH) and (c) from experiment, and resonances from ring protons using (d) NMR spin simulation and (e) from experiment. The ^1^H NMR chemical shift for H2 is 3.562 ppm, and that of H3 is 3.560; at temperatures of either 60 or 10 °C, the anomeric proton retains its simple *doublet* appearance due to the ^3^*J*_H1,H2_ coupling constant of 7 Hz.

The limited ^1^H NMR spectral dispersion of glycans lead to second-order effects that appear as changes in intensities as well as in splitting of *J* coupled nuclei, in comparison to first-order spectra where the chemical shift difference Δν_AB_ between nuclei A and B is one order of magnitude larger than *J*_AB_. To obtain accurate ^1^H chemical shifts with high decimal place precision^402^ and ^*n*^*J*_HH_ coupling constants, quantum mechanical computerized spectral analysis can be performed.^403^^1^H NMR spectra are then simulated by an iterative process using, e.g., PERCH^404^ and compared to an experimental spectrum (Figure 41), and once the residual root-mean-square has been minimized between the observed and calculated spectra, both chemical shifts (δ_H_) and ^n^*J*_HH_ values can be obtained with confidence.

As anomeric ^1^H NMR resonances most often resonate at a higher chemical shift than those from other nonexchangeable protons of a carbohydrate molecule, virtual coupling is conspicuous if present.^405^ The presence of virtual coupling may be due to subtle and small changes in chemical shifts due to, e.g., temperature changes and can thus produce different spectral appearances as seen for β-d-Glc*p*NAc-(1→4)-β-d-Glc*p*NAc-OMe,^406,407^ where for the reducing end residue at 5 °C δ_H2_ 3.73 and δ_H3_ 3.68 differ in chemical shifts and δ_H1_ 4.43 with ^3^*J*_H1,H2_ = 8.6 Hz appears as a regular resolved doublet. However, at a higher temperature of 70 °C, the corresponding chemicals shifts of H2 and H3 both resonate at 3.71 ppm, and virtual coupling appears at H1 due to the degenerate chemical shifts with ^3^*J*_H2,H1_ = 8.4 Hz and ^3^*J*_H2,H3_ = 10.0 Hz in the spin system.

An even more extreme appearance of the resonance from an anomeric proton was observed for 2-naphthyl 4-*C*-methyl-β-d-xylopyranoside^408^ in the ^1^H NMR spectrum at 37 °C, where it showed five peaks (Figure 41b,c) instead of the simple doublet which is present at both 10 and 60 °C. The chemical shift difference between H2 and H3 is a mere 0.002 ppm at 37 °C with δ_H2_ 3.562 and δ_H3_ 3.560 (Figure 41d,e) and ^3^*J*_H2,H1_ = 6.8 Hz and ^3^*J*_H2,H3_ = 8.7 Hz, as deduced by NMR spin simulation using PERCH.

By parametrizing ^1^H NMR chemical shifts and coupling constants for a compound into a “spin system matrix” the characterization will be independent of spectrometer frequency and line shape, which subsequently facilitates simulation of spectra at other magnetic field strengths than originally acquired. This approach has been implemented in GISSMO, which enables calculation and refinement of spin system matrices.^409^ In the analysis of the ^1^H NMR chemical shifts and coupling constants of sucrose, the spins of the two sugar residues were divided into submatrices, one for the glucose residue and one for the fructose residue. For those spins that showed spectral overlap in the ^1^H NMR spectrum traces from the 2D ^1^H,^13^C-HSQC NMR spectrum made it possible to separate the overlapping resonances and to optimize them individually. Subsequent merging of submatrices produced a simulated spectrum in very good agreement with the experimental ^1^H NMR spectrum of sucrose.

Quantum mechanical ^1^H iterative full spin analysis (HiFSA) has been used to analyze in detail the ^1^H NMR spectra of the bidesmosidic flavonol triglycoside kaempfenrol-3-*O*-robinoside-7-*O*-glucoside, in which there are two points of attachment for saccharide components, one of which being β-d-Glc*p* and the other is the disaccharide α-l-Rha*p*-(1→6)-β-d-Gal*p*.^42^ Spectral analysis was performed at low 60 MHz, intermediate 600 MHz as well as high 900 MHz ^1^H resonance frequencies using different polar deuterated binary solvent mixtures of DMSO-*d*_6_ with methanold-*d*_4_ or D_2_O in comparison to neat DMSO-*d*_6_, which simplified the analysis and facilitated structure elucidation. The HiFSA methodology was also applied in the structural investigation of monoterpene diglycosides containing α-l-Ara*p*-(1→6)-β-d-Glc*p* or α-l-Ara*f*-(1→6)-β-d-Glc*p* linked to different C_10_-aglycones.^410^ The NMR spin simulation analysis was used for interpretation of ^1^H NMR spectra, where small chemical shift changes of ∼0.1 ppm were observed between protons in the methylene group of the primary carbon atom of the aglycone at the glycosidic linkage in comparison to (−)-myrtenyl α-l-arabinopyranosyl-(1→6)-β-d-glucopyranoside. It was concluded that the compound investigated was (+)-myrtenyl α-l-arabinopyranosyl-(1→6)-β-d-glucopyranoside, i.e., the aglycone moiety was the enantiomeric counterpart to that previously determined. The study underscored the importance of relative chemical shifts as indirect structural evidence.

#### 5.3.3 Carbohydrate Mixtures

Determination of saccharide components as mixtures employ a range of NMR techniques depending on whether monosaccharide hydrolysates, a distribution of oligosaccharides or different polysaccharides are to be analyzed. Quantitative determination of the sugar components in cellulose and hemicellulose polysaccharides was optimized by using a two-step hydrolysis procedure employing deuterated sulfuric acid and analyzed by ^1^H NMR spectroscopy.^411^ For the complex saccharide mixtures present in honey consisting of mono-, di-, and trisaccharides, an approach based on ^1^H,^1^H-CSSF-TOCSY NMR experiments was chosen.^412^ Prior to analysis of the unknown mixture, optimal frequencies for selective excitation at a resonance frequency characteristic of each sugar residue, typically from the anomeric protons, were determined on standard solutions of saccharides and the identity of > 20 mono- to trisaccharides could be ascertained as well as quantified. In an alternative approach, monosaccharide composition of glycans was investigated by quantitative ^1^H,^13^C-HSQC NMR experiments, which focused on the spectral region for anomeric resonances; a ^1^*J*_C1,H1_ value of 155 Hz was judged suitable for the Q-HSQC experiment.^413^ Using this methodology, sugar components were determined in hydrolysates of complex polysaccharides from gum in plants and from an exopolysaccharide of a plant-associated bacterium. Focus on the anomeric region was also the case in resolving starch fragments by ^1^H,^13^C-HSQC NMR experiments using a narrow 3 ppm ^13^C spectral width, which allows sampling the indirect *F*_1_ dimension at high resolution.^414^

To unravel differently sized glycans in a mixture, one may rely on the variation in their translational diffusion coefficients and ^1^H NMR experiments based on diffusion-ordered spectroscopy (DOSY), in which the second dimension is encoded by the translational diffusion coefficient (*D*_t_), offers a powerful approach. Differentiation of maltooligosaccharides with a degree-of-polymerization (dp) of 3–4 and arabinoglycan with dp > 50 in beer,^415^ as well as analysis of glucose in fruit juices,^416^ have utilized DOSY NMR experiments to this end. 2D-DOSY experiments were also successfully used to differentiate the α- and β-anomeric forms of d-glucose, other monosaccharides, the anomeric forms of cellobiose, and different phenyl d-glucosides.^417^ The additional use of transverse (*x*,*y*) pulsed-field gradients (PFGs) in conjunction with the standard *z*-axis PFG can be used to reduce the impact of sample convection and to minimize gradient-dependent line shape distortions in 2D-DOSY NMR experiments, as exemplified for a mixture of mono- and oligosaccharides.^418^

In cases where the translational diffusion coefficients are closely similar and a DOSY experiment will not differentiate the components, one would need to rely on a different physicochemical property such as NMR spin relaxation, referred to as relaxation-ordered spectroscopy (ROSY). In the relaxation-encoded selective TOCSY (REST) class of experiments one combines selective excitation and isotropic mixing to label each spin system with the same relaxation weighting based on, e.g., transverse *T*_2_ or longitudinal *T*_1_ relaxation times.^419^ Using selective excitation at ∼ 5.24 ppm of the reducing end glucosyl residue (α-anomeric form) of the (1→4)-linked lactose and the (1→6)-linked melibiose, the REST_2_ experiment facilitated differentiation between the two disaccharides based on their transverse relaxation times when analyzed in combination with multivariate processing. By combining REST with pure shift using the PSYCHE *J*-refocusing element, 2D NMR experiments referred to a PUREST-*T*_1_ or PUREST-*T*_2_, depending of the relaxation mechanism, can be obtained. A mixture of d-xylose and l-arabinose containing five major species could unambiguously be distinguished from 2D PUREST spectra.^420^ Other developments along these lines are described in a methodology referred to as SCALPEL,^421^ in which proton spins are first TOCSY-*t*_1_ encoded using only a small number of *t*_1_-increments, followed by translational diffusion encoding and *T*_1_ or *T*_2_ relaxation encoding in a block that also contains 180° selective refocusing pulses, and finally a TOCSY block in the pulse sequence prior to acquisition of the FID. The effect of the narrow bandwidth selective TOCSY pulse sequence in conjunction with multivariate analysis makes it possible to extract contributions from each of the different species in a mixture, whether they are oligosaccharides present in beer or in any other combination of saccharides where a single property would not suffice to differentiate the components.

A mixture of the three monosaccharides, d-glucose, d-mannose, and l-rhamnose, presents a quite complex ^1^H,^1^H-TOCSY NMR spectrum when a long mixing time of ∼ 100 ms is employed, i.e., six spin systems with 6 or 7 signals for each anomeric form. In order to speed up the acquisition of the 2D PSYCHE-TOCSY NMR experiment,^335^ retaining homonuclear decoupling in the indirect dimension where best needed, a band-selective excitation version (BSE-PSYCHE-TOCSY) NMR experiment^422^ was proposed whereby an *F*_1_-decoupled spectral region was obtained for chemical shifts within the excited band, such as between 3–4 ppm for carbohydrates, where the ring-protons and those from hydroxymethyl groups reside. The well-resolved anomeric protons and those from the methyl group of rhamnose are left unperturbed with respect to the homodecoupling. Subsequent application of indirect covariance matrix processing results in that also the *F*_2_-dimension becomes decoupled and a pure shift spectral region is attained within the excited band. The gain in acquisition time for the BSE-PSYCHE-TOCSY experiment is one order of magnitude in comparison to the broadband version of the experiment. In another approach, a mixture of d-glucose and d-xylose in high and low concentration, respectively, was used as a test case for complete chemical shift assignments using the NOAH-AST experiment,^423^ where the AST abbreviation refers to: A, 1,1-ADEQUATE; S, multiplicity-edited HSQC; T, TOCSY. For d-glucose, the anticipated correlations were observed in all spectra, whereas due to the low concentration of d-xylose in the sample preparation, only the proton-detected experiments showed correlations from the latter sugar under the experimental conditions used. In a study using model compounds, such as glucose, glucitol, and mannitol to represent biomass-derived complex mixtures, it was shown that a combination of 1D PSYCHE and 1D TOCSY-PSYCHE experiments were powerful in ^1^H NMR resonance assignments of the constituents of the mixture.^424^

## 6 Computer-Assisted Structural Elucidation of Glycans

### 6.1 Databases

NMR chemical shifts of carbohydrates in databases are valuable assets in elucidating and identifying glycan structures. In the GLYCOSCIENCES.de database, > 3000 NMR spectra have been deposited, and these are stored as lists of chemical shifts.^425^ By defining an NMR chemical shift range, the database can be queried for a specific carbohydrate residue. Furthermore, the peak search option compares a user-provided list of chemical shifts to be compared to those in the database to obtain NMR spectral information best matching the input data for the query. The carbohydrate structure database (CSDB) is based on ∼ 10000 covering > 25000 compounds from 13000 organisms.^426^ There are several ways to make a search query, inter alia, “(sub)structure” or “composition” as well as “NMR signals”. For the latter, a list of either ^13^C or ^1^H NMR chemical shifts can be entered in the query, which then returns both glycan structure and chemical shifts if matching data can be found.

A different approach was used for the database sum of anomeric chemical shifts (SOACS) and SOACS-ol, where the latter is to be used when the glycan has been reduced, e.g., for mucin type O-linked glycans released by β-elimination.^427^ An index number is calculated based on the sum of the ^1^H NMR chemical shifts of the anomeric protons as well as H3_ax_ resonances if sialic acids are present. With increasing number of constituent monosaccharides, the values of the SOACS and SOACS-ol indexes increase. The database has a focus of multibranched oligosaccharides containing a GalNAc-ol residue. In the database *Escherichia coli* O-Antigen Database (ECODAB), structures are stored of the O-polysaccharides of the lipopolysaccharides from *E. coli*.^188,428^ In addition, ^1^H and ^13^C NMR chemical shift data of the O-antigens together with a search query function makes it possible to retrieve structures with corresponding chemical shifts, which may be highly useful for clinical isolates that have not been serotyped and may belong to an already defined O-antigen group.^429^

Based on the GLYCOSCIENCES.de database, which contains > 16000 monosaccharide entries, a search algorithm was developed, viz., GlycoNMRSearch.^430^ Matching is performed using either subsets or the entire set of chemical shifts for monosaccharide spin systems. Connectivities rely on ^1^H,^13^C-HSQC spectra in combination with, e.g., ^1^H,^1^H-TOCSY or ^1^H,^13^C-HSQC-TOCSY spectra to assign carbohydrate spin systems by 2D NMR spectra. The results consist of top-ranked structures containing sugar residue(s), linkage position(s), and anomeric configuration(s).

### 6.2 NMR Chemical Shift Predictions

Tools for ^1^H and ^13^C NMR chemical shift predictions are valuable for structural confirmation of synthesized glycans, support of NMR resonance assignments, and approaches for structural elucidation relying on acquired NMR data. GlyNest uses the GLYCOSCIENCES.de database to estimate chemical shift, and it is based on a spherical environment encoding scheme for each atom.^425,431^ A semiautomated NMR-based method that uses unassigned ^13^C NMR spectra in conjunction with other methods is known as Generation, Ranking and Assignment of Saccharide Structures (GRASS).^432^ It performs a two-step procedure in which a rough ranking against the ^13^C NMR spectrum is carried out first, followed by an accurate simulation method for refinement of the chemical shifts. Besides the ^13^C NMR spectrum, additional NMR data should be added, if available, to enhance the accuracy of the chemical shift prediction. GRASS has been implemented as part of CSDB, and for top-ranked structure suggestions one can obtain predicted ^13^C and ^1^H NMR chemical shifts. The NMR-based structure elucidation can be complemented by visualization of 2D NMR spectra using the software Glycan Optimized Dual Empirical Spectrum Simulation (GODESS),^433^ also being a part of CSDB. CASPER predicts ^1^H and ^13^C NMR chemical shifts^318^ based on increment rules^434^ and uses all chemical shifts in a monosaccharide in conjunction with chemical shift differences of disaccharides vs monosaccharides, i.e., glycosylation shifts, as well as any chemical shift changes in trisaccharides compared to those of the constituent disaccharides to estimate the chemical shifts of oligo- and polysaccharides. The web-based program^435^ can be used in three main ways: (i) prediction of NMR chemical shifts for a given glycan structure, (ii) component analysis based on NMR chemical shifts of saccharide mixtures from an oligo- or polysaccharide hydrolysate giving reducing monosaccharides, or methanolysis resulting in methyl glycosides or butanolysis using optically active 2-butanol with ensuing 2-butyl glycosides. Analysis of unassigned ^1^H,^13^C-HSQC spectra (peak-picked cross-peaks to obtain chemical shifts and one-bond correlations) of the diastereomeric glycosides facilitates determination of both the sugars present in the mixture and their absolute configuration(s) by NMR spectroscopy.^179^ (iii) Structural determination of a glycan can be performed by using as input a component analysis performed by NMR spectroscopy (vide infra) or any other method and unassigned 1D ^1^H and/or ^13^C NMR chemical shifts in conjunction with connectivities between nuclei obtained from 2D NMR experiments such as ^1^H,^13^C-HSQC or ^13^C,^1^H-HETCOR, ^1^H,^1^H-TOCSY with several mixing times or a long mixing time (∼ 80 ms), ^1^H,^13^C-H2BC or ^1^H,^13^C-HSQC-TOCSY with a short mixing time (10 ms), and ^1^H,^13^C-HMBC experiments. Additional NMR data such as coupling constants of anomeric protons, ^3^*J*_H1,H2_ and ^1^*J*_H1,C1_, may be utilized to speed up the calculations and to rule out structural combinations that are not consistent with experimental data. Structural suggestions are ranked according to best fit between experimental and predicted NMR data, resulting also in that tentative ^1^H and ^13^C NMR chemical shift assignments are obtained. Predefined structural elements such as the N-glycan pentasaccharide core Man_3_GlcNAc_2_, the tetrasaccharide repeating unit of *Shigella flexneri* O-antigen polysaccharides, or biosynthetic considerations for the O-antigen assembly in, e.g., *E. coli*, may be applied. Different substituents at sugar residues and methyl glycosides as well as some glycan-amino acid structures are also handled by the CASPER program.^436,437^ 3D model of the proposed structure of the glycan investigated can subsequently be generated by CarbBuilder^438,439^ as part of the output results and visualized by a standalone molecular graphics program.

## 7 Technological Developments

### 7.1 Cryogenically Cooled Probes and Microcoils

NMR spectroscopy has been limited by its low sensitivity, which to some extent can be alleviated by signal averaging, although this leads to long experimental times. A significant improvement in sensitivity took place with the introduction of cryogenically cooled probes, in which the thermal noise in the radiofrequency coil is reduced by lowering the temperature to ∼ 20 K as well as by cooling the preamplifier electronics.^440,441^ An NMR sample can, however, be analyzed in the temperature range −40 °C to +80 °C. The sensitivity gain is on the order of a factor of 4 but will be reduced if sample solutions have a high ionic content. To mitigate the loss of sensitivity, low conductivity buffers may be used^442^ or NMR tubes of a smaller diameter such as 3 mm in a probe designed for 5 mm NMR tubes may be employed, as exemplified for sucrose in D_2_O devoid of salt or in the presence of 4 M NaCl.^443^ Using a 3 mm tube in a “5 mm probe” is highly beneficial for sample-limited studies of oligosaccharides because the signal-to-noise ratio is only affected to a small extent, even though the amount of material is reduced by one-third for the smaller diameter sample.^444^ Dedicated cryogenic probes with a sample tube diameter of 1.7 mm gives a sensitivity gain by more than one order of magnitude, compared to a standard 5 mm probe operating at room temperature. Cryogenically cooled broadband probes have increased signal-to-nose ratio compared to those operating at room temperature, and when optimized for carbon nuclei, the improved resolution due to direct ^13^C-detection offers a valuable complement or even an alternative to the ^1^H-detected heteronuclear correlation experiments in studies of carbohydrate structure.

Small amounts of sample are favorably analyzed using 3 mm outer diameter NMR tubes in probes dedicated to this end and a narrower 1.7 mm NMR tube may also be used in the same probe; the approach was employed in the analysis of large hydroxy-proline arabinogalactans having branched side-chains with different substitution patterns.^445^ For the smallest amount of material microcoil NMR probe technology can be utilized to obtain improved mass sensitivity.^446^ This methodology was applied for characterization of mass-limited heparin-derived oligosaccharides analyzed by ^1^H and standard 2D ^1^H,^1^H-correlated NMR experiments. Glycan structure can thus be investigated from microgram quantities of material, e.g., tetrasaccharides with a molecular mass of ∼ 1100–1200 Da were analyzed using ∼ 20 μg of sample in 3 μL of D_2_O.^447^

### 7.2 Dynamic Nuclear Polarization

The sensitivity of the NMR technique is low, and for spin-1/2 nuclei commonly used to investigate carbohydrate structure only a small fraction of the nuclear spins will contribute to the NMR signal, i.e., the polarization (*P*) of spins is low. This can be described by *P* = (*N*_α_ – *N*_β_/*N*_α_ + *N*_β_), where *N*_α_ and *N*_β_ are the number of spins in the lower and higher energy states, respectively, and at thermal equilibrium, the population of the spins follow a Boltzmann distribution. For the two energy levels, the polarization can also be given by *P* = tanh(*ℏγB*_0_/2*k*_B_*T*), where *ℏ* is Planck’s constant divided by 2π, γ is the nuclear magnetogyric ratio, *B*_0_ is the applied magnetic field, *k*_B_ is the Boltzmann constant, and *T* is the absolute temperature.^448^ Thus, as the magnetic field increases and the temperature decreases, the nuclear spin polarization increases but is still low for the nuclei with a resonance frequency in the MHz range. However, the frequency in electron spin resonance employing microwave irradiation is significantly higher by a few orders of magnitude.

**Figure 42:**
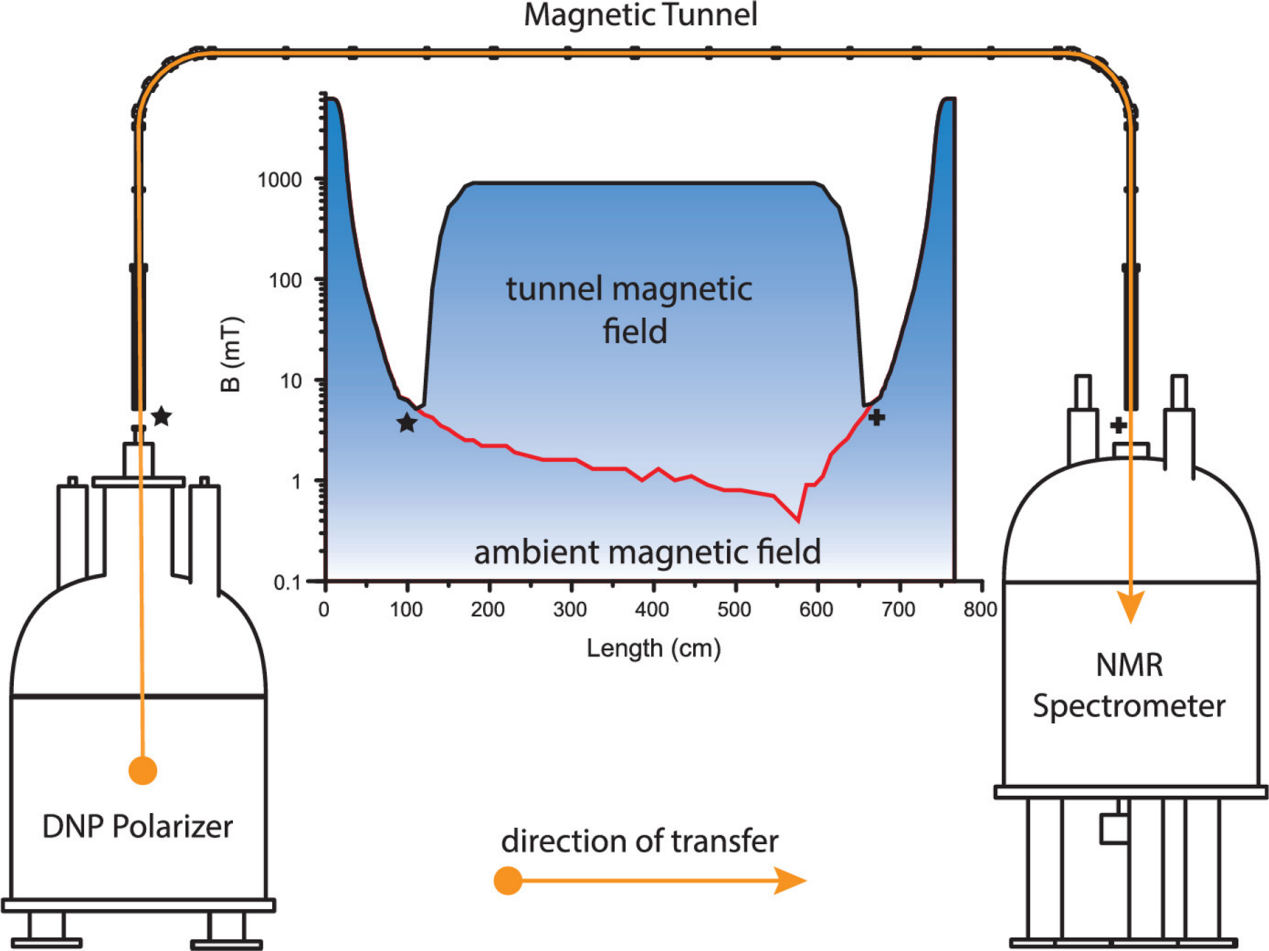
Schematic diagram of a combined dynamic nuclear polarization setup for liquid state NMR spectroscopy. The sample is hyperpolarized in the cold magnet system (left) and transferred by a stream of hot solvent into the NMR system (right) for data acquisition with improved sensitivity. The magnetic field strength during the transfer of the hyperpolarized fluid through a magnetic tunnel (black line) or without tunnel (red line) is shown as an insert. Reprinted with permission from ref (455). Copyright 2015 Authors.

Dynamic nuclear polarization (DNP)^449−451^ relies on the fact that microwave irradiation of an electron paramagnetic agent (EPA, which is in the form of an organic free radical) together with the target substance, both of which are dispersed in a glassy state at low temperature of ∼ 1 K, leads to a close to unit polarization transfer from the electrons to the nuclei of interest; as a result, an increase of the nuclear polarization occurs, improving the sensitivity of the resonances when detected by NMR spectroscopy. The polarization transfer in the dissolution DNP (dDNP) technique^448,452^ is often carried out in a separate system, e.g., with a relatively low magnetic field of 3.35 T and an irradiation frequency of 94 GHz, although higher magnetic fields (6.7 T) and irradiation frequencies (188 GHz) have also been used.^453^ This is followed by dissolution using a superheated solvent and transfer via a “magnetic tunnel” to an NMR spectrometer, where the experiment is carried out (Figure 42).^454,455^ The nuclei chosen, ^13^C or ^15^N, have a relatively low γ and the target compounds are present in M concentration, whereas the organic compound with a free radical is present in mM concentration. Perdeuteration of the target molecule naturally extends the ^13^C longitudinal relaxation time *T*_1_ due to the absence of heteronuclear ^1^H,^13^C dipolar relaxation, and this is beneficial both during and after the transfer from the polarizer.^453^ An alternative way to transfer the hyperpolarized sample is to keep it frozen, transfer it via the “magnetic tunnel” (duration ≤ 70 ms) using pressurized helium gas, and upon arrival in the second magnet let it dissolve rapidly in a preheated solvent, whereafter the sample solution is drawn into the NMR tube (duration < 1 s) and the recoding of NMR spectra is initiated; the technique has been dubbed “bullet-DNP”.^456,457^ Furthermore, ^13^C NMR signals from small biological molecules, exemplified inter alia by [UL-^13^C_6_]glucose, can be enhanced by in situ Overhauser DNP in water at room temperature,^458^ and hyperpolarized water in a dDNP experiment can be used for acquiring in a single scan a ^15^N NMR spectrum of urea at natural abundance;^459^ likewise, hyperpolarized water is able to boost sensitivity in biomolecular NMR by acting as a hyperpolarization agent, whereby labile protons on the target molecule are exchanged with those of the hyperpolarized solvent.^460^

The dDNP technique has been used successfully to study enzymatic reactions involving sugars, where the outstanding sensitivity and speed of the experiments made it possible to observe products and intermediates not previously detected. In a study of phosphorylation of glucose by hexokinase in the presence of magnesium ions and ATP resulting in d-glucose-6-phosphate, which also is an inhibitor of the enzyme, the dDNP methodology was employed.^461^ Specifically, to investigate the kinase reaction d-[UL-^13^C_6_;UL-^2^H_7_]glucose and the radical TEMPOL were mixed, frozen, and subjected to microwave irradiation at ∼ 1 K. After rapid dissolution, the hyperpolarized substrate was transferred to the second magnet and injected into a buffer solution containing reactants and the hexokinase enzyme. The ^13^C NMR spectra were acquired every second, with deuterium decoupling using 10° radio frequency pulses, for ∼ 20 s; the uniformly ^13^C and ^2^H labeled reactant d-glucose and product d-glucose-6-phosphate, both present in equilibrium between the α- and β-anomeric forms, had ^13^C *T*_1_ relaxation times in the range of 2–4 s. The presence of products and kinetics of the reaction were monitored using signals from the anomeric carbon-13 nuclei, where the products showed small chemical shift displacements toward higher chemical shifts, e.g., the C1-signal of the β-anomeric form of d-glucose-6-phosphate was shifted by ∼ 0.2 ppm compared to the corresponding signal from d-glucose, a chemical shift difference that was sufficient to distinguish resonances and to follow the time course of the reaction. Importantly, both anomeric forms of glucose, which interconvert on a time scale of several minutes, were phosphorylated, and it was possible to extract kinetic parameters for the kinase reaction from the NMR experiments lasting only ∼ 20 s.

**Figure 43:**
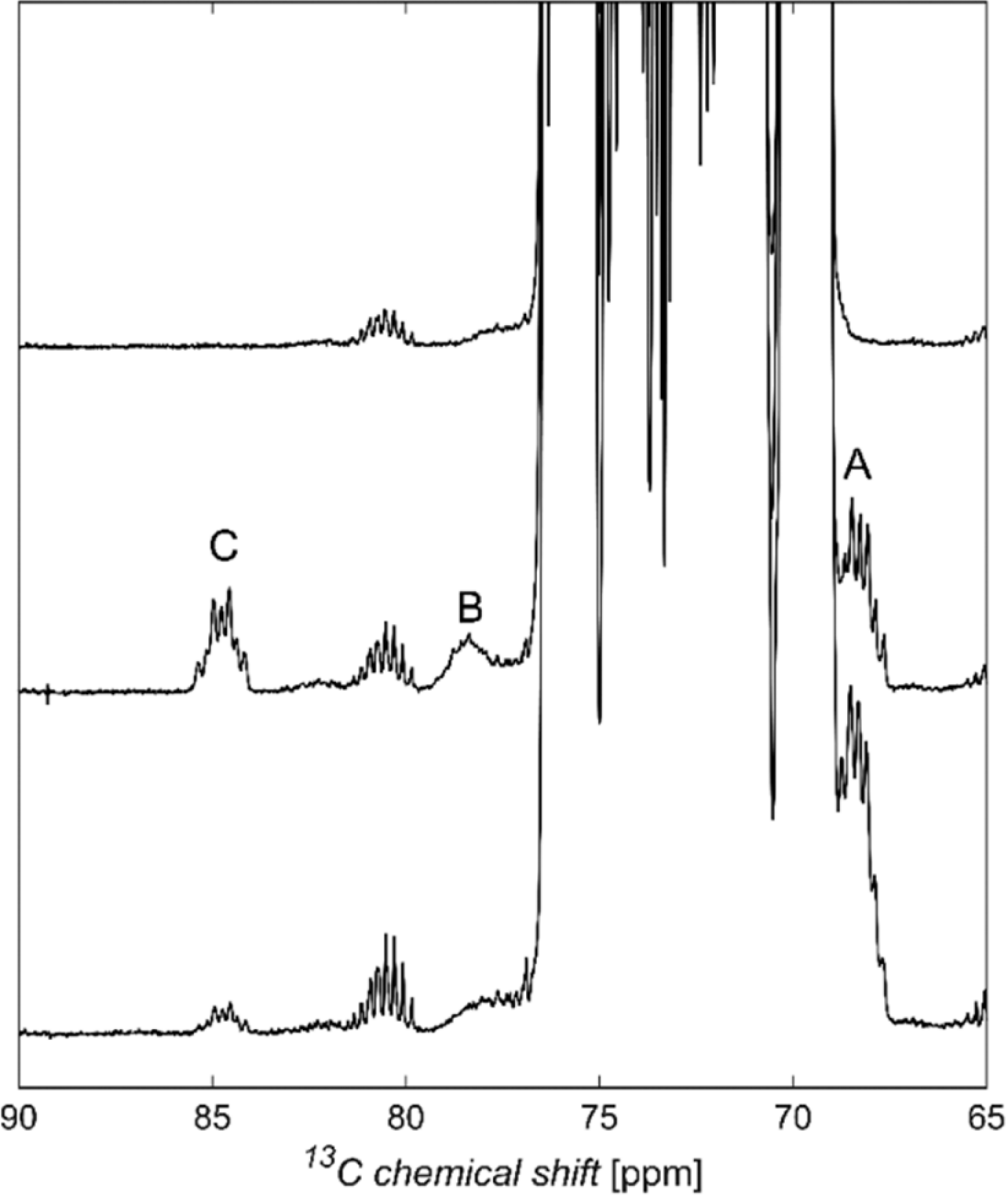
Dissolution dynamic nuclear polarization (dDNP) NMR spectroscopy in which the ^13^C spectra are summed 4–18 s after transfer to the NMR tube. (top) Hyperpolarized d-[UL-^13^C;UL-^2^H]glucopyranose without enzyme or donor molecule, (middle) mixed with *ortho*-nitro-phenyl β-d-galactopyranoside and lactozyme 2600L, and (bottom) mixed with the donor and *lacZ* β-galactosidase. The ^13^C resonances labeled by A, B, and C correspond to the substitution position in 6-substituted glucose, 4-substituted glucose, and 3-substituted β-d-glucose, respectively. Reproduced with permission from ref (463). Copyright 2020 American Chemical Society.

Glycosidases hydrolyze oligo- and polysaccharides, but this class of enzymes can also be used in transglycosylation reactions whereby a new glycosidic linkage is formed to an acceptor sugar, as shown by recent dDNP NMR studies of β-galactosidases.^462,463^ Enzymes from glycoside hydrolase family 2 have a double displacement mechanism with retention of anomeric configuration, and consequently any transglycosylation products from the action of *lacZ* β-galactosidase or the enzyme mixture Lactozyme 2600L should lead to galactosyl-containing products having the β-anomeric configuration. The experimental setup included after polarization and dissolution, inter alia, 20° or 30° ^13^C radio frequency pulses applied with a repetition time of 2 s to the sample mixture containing the enzyme β-galactosidase and the reactants being a donor glycoside and a monosaccharide acceptor molecule, one of which was a ^13^C,^2^H-isotopically labeled monosaccharide entity. Notably, in the first scan, the signal enhancement was ∼ 10^4^, thereby facilitating detection of products and transient intermediates that would not have been possible to reveal otherwise. The transglycosylation reactions and subsequent hydrolysis of products formed were first studied^462^ using the site specifically isotope labeled *o*-nitrophenyl β-d-[1-^13^C;1-^2^H]galactopyranoside as a donor molecule and galactose as an acceptor (Scheme 1). Analysis of the region in ^13^C NMR spectra where anomeric carbon resonances reside revealed β-d-[1-^13^C;1-^2^H]Gal*p*-(1→6)-d-Gal*p* as the major transglycosylation product, β-d-[1-^13^C;1-^2^H]Gal*p*-(1→4)-d-Gal*p*, and/or β-d-[1-^13^C;1-^2^H]Gal*p*-(1→3)-d-Gal*p* and most interestingly the trehalose-type disaccharide β-d-[1-^13^C;1-^2^H]Gal*p*-(1↔1)-β-d-Gal*p*. Further analysis of acquired NMR data enabled the determination of relative transglycosylation and hydrolysis rates, where β-d-[1-^13^C;1-^2^H]Gal*p*-(1→6)-d-Gal*p* was formed at the highest rate and β-d-[1-^13^C;1-^2^H]Gal*p*-(1↔1)-β-d-Gal*p* was hydrolyzed at the highest rate of the disaccharides produced. The second study used instead natural abundance *o*-nitrophenyl β-d-galactopyranoside and d-[UL-^13^C_6_;UL-^2^H_7_]glucose (Scheme 1), together with *lacZ* β-galactosidase as well as with the enzyme mixture Lactozyme 2600L.^463^ In this case, the uniformly ^13^C and ^2^H labeled glucose allowed for analysis of the ^13^C spectral region 65–85 ppm, thereby identifying β-d-Gal*p*-(1→6)-d-[UL-^13^C_6_;UL-^2^H_7_]Glc*p* (allolactose) as the major product, β-d-Gal*p*-(1→4)-d-[UL-^13^C_6_;UL-^2^H_7_]Glc*p* (lactose) and β-d-Gal*p*-(1→3)-d-[UL-^13^C_6_;UL-^2^H_7_]Glc*p* (Scheme 1, Figure 43). In particular, the latter disaccharide was observed as β-d-Gal*p*-(1→3)-β-d-[UL-^13^C_6_;UL-^2^H_7_]Glc*p*, i.e., the anomeric configuration of the reducing end sugar was β for the transglycosylation product, demonstrating that the enzyme has selectivity for that anomeric form of the acceptor. Moreover, the obtained NMR data were used to determine the relative formation ratios as well as the hydrolysis rates for the three disaccharides.

### 7.3 Low Field Magnets, High-Temperature Superconductors, and High Field Magnets

**Figure 44:**
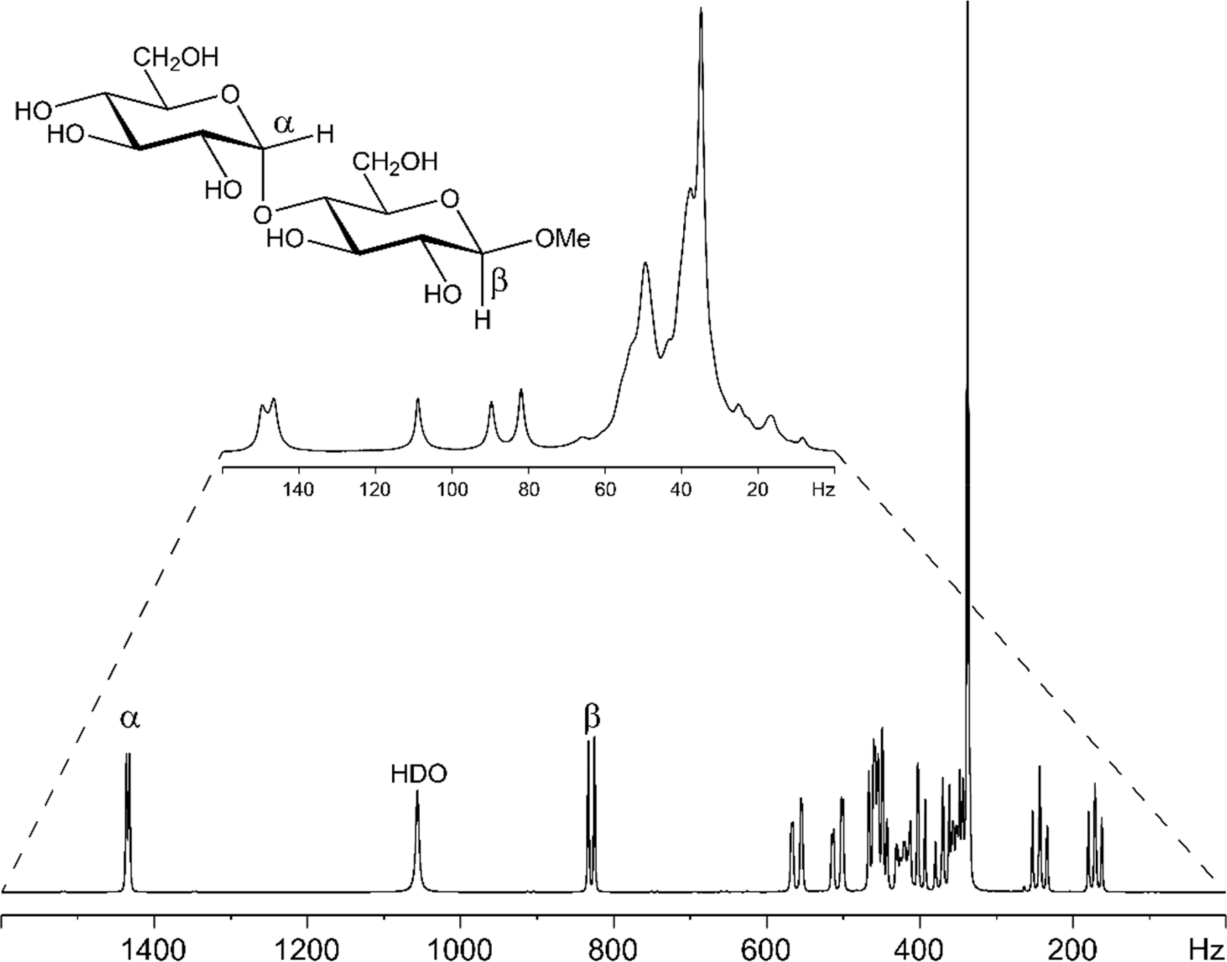
Low and medium magnetic fields used for ^1^H NMR spectra of methyl β-maltoside in D_2_O at 26 °C and a ^1^H spectrometer frequency of 60 MHz (top) and 600 MHz (bottom). The ^1^H NMR chemical shift at 3 ppm was set to 0 Hz.

The revival of NMR spectrometers operating at low ^1^H Larmor frequencies in the range 43–100 MHz^464^ has during the past decade opened a niche complementing the NMR spectrometers with ^1^H frequencies of 300 MHz or higher. These low frequency benchtop systems have permanent magnets, in contrast to the higher frequency systems that utilize low-temperature superconducting (LTS) magnets at 4.2 K. The benchtop NMR spectrometers are compact with a small size and do not require cryogens for their operation. Most of the commercially available benchtop spectrometers utilize standard 5 mm outer diameter NMR tubes and can detect ^1^H and/or different NMR active nuclei such as ^13^C, ^15^N, or ^31^P present in glycans. Notably, the common homo- and heteronuclear 2D NMR experiments have also been implemented. The recent development for benchtop systems that facilitates NMR spectra to be acquired at different elevated temperatures is a very important improvement because for the NMR analysis of carbohydrates molecules in D_2_O, the fact that the ^1^H chemical shift of the HDO peak is very sensitive to temperature makes it possible to avoid spectral overlap between, in particular, the HDO peak and resonances from anomeric protons of an oligosaccharide. However, the dispersion of resonances in ^1^H NMR spectra is low at 60 MHz as compared to, e.g., 600 MHz (Figure 44), but the anomeric configuration of pyranosides with the *gluco*/*galacto* configuration can readily be determined at the lower frequency.

**Figure 45:**
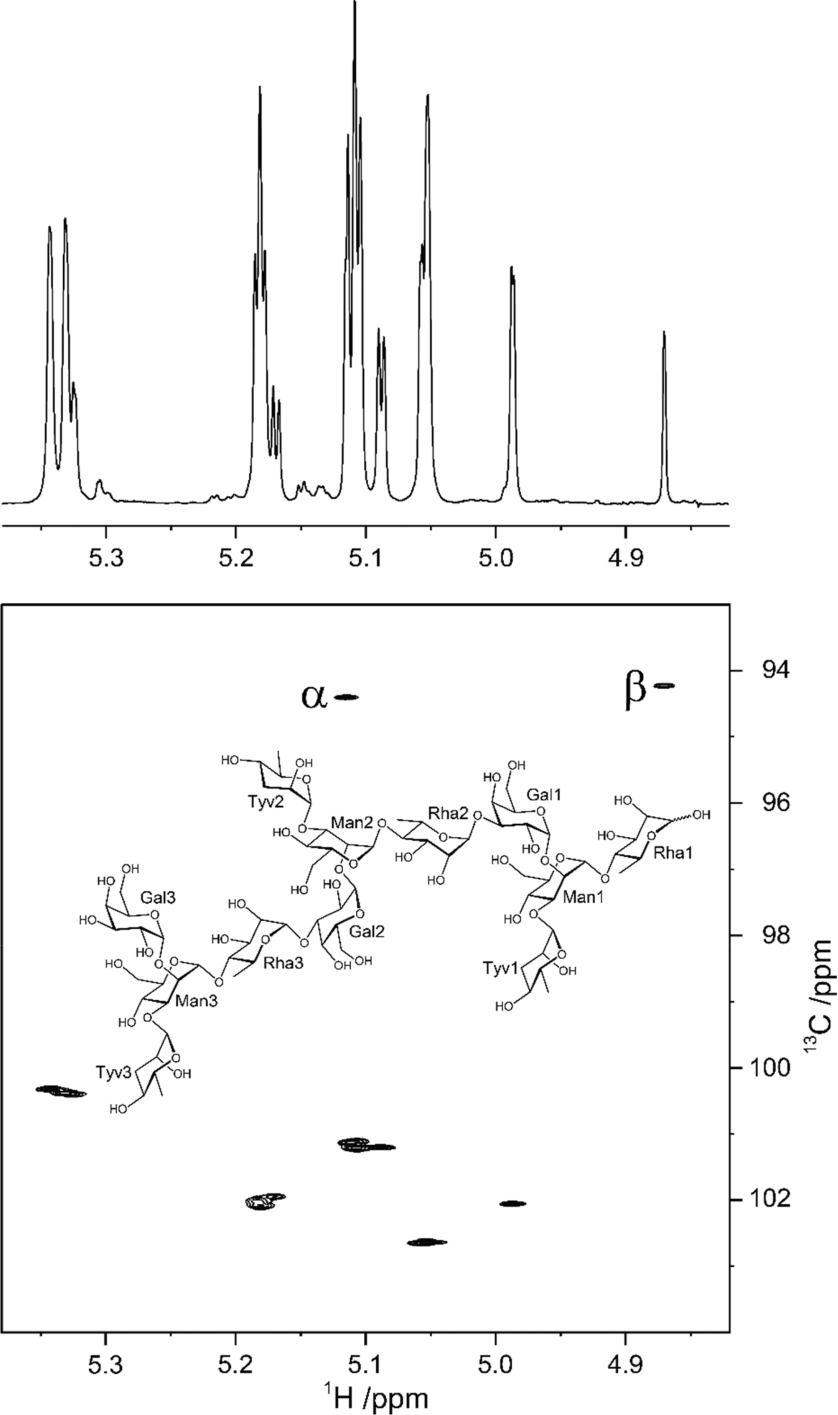
High magnetic field used for ^1^H (a) and ^1^H,^13^C-HSQC (b) NMR spectra (anomeric region) of the dodecasaccharide (anomeric mixture at the reducing end) in D_2_O at 25 °C and a ^1^H spectrometer frequency of 900 MHz. Its structure corresponds to three repeating units of the *Salmonella enteritidis* O-antigen with the sequence →3)-α-d-Gal*p*-(1→2)-α-d-Man*p*-(1→4)-α-l-Rha*p*-(1→, to which tyvelose (3,6-dideoxy-d-*arabino*-hexopyranose) groups are α-(1→3)-linked to each of the mannosyl residues. The rhamnosyl residue at the reducing end of the dodecasaccharide is present as a mixture of anomeric forms.

In the early 2000s, high field NMR LTS magnets operating at a ^1^H frequency of 800 MHz had become available to researchers in the field. The earlier wires used for construction of LTS magnets were made from alloys of niobium and titanium which facilitated operation at 400 MHz, but switching to alloys made from niobium and titanium complemented with other elements such as tantalum made it possible to reach significantly higher magnetic fields.^465^ Further increase of the magnetic field strength corresponding to a ^1^H frequency of 900 MHz was promoted by cooling the NMR coil using subcooled superfluid helium at a temperature of ∼ 2 K. At a ^1^H frequency of 900 MHz, the dispersion of resonances increases significantly, which is highly beneficial in structural studies of complex oligosaccharides,^466^ although some spectral overlap of resonances still occurs for closely similar structural elements in oligosaccharides originating from polysaccharides with repeating units (Figure 45). A decade later, the first 1 GHz NMR spectrometer made its appearance.^467^ However, above this magnetic field of ∼ 23.5 T, the critical current density for Nb_3_Sn-based alloys decreases steeply and superconductivity will disappear. To reach even higher magnetic fields with ultra-high field NMR spectrometers operating at 1.1 and 1.2 GHz hybrid designs have been developed, whereby high-temperature superconductors (HTS) using “copper-oxides” are utilized in the inner section of the solenoid magnet and LTS in the outer portion of the magnet. ^1^H,^15^N-SOFAST-HMQC and ^1^H,^15^N-BEST-TROSY NMR spectra of proteins at 1.2 GHz have been acquired and were compared with respect to resolution and sensitivity to spectra obtained at 900 and 950 MHz, which resulted in clear improvements at the highest magnetic field;^468^ these results are promising for future applications to glycans using ultra-high field NMR spectroscopy at > 1 GHz. To obtain even higher magnetic field strengths for NMR spectrometers operated in a persistent mode, it will be essential to construct high quality superconducting joints between HTS coils as well as between HTS and LTS wires in order to reach such goals.^469,470^ Furthermore, the HTS can be used in another very interesting area of NMR spectroscopy, viz., in the construction of cryogen-free power-driven magnets, as was shown by an HTS magnet operating at 9.4 T corresponding to a ^1^H frequency of 400 MHz.^471^ The operation temperature of the magnet is 14–18 K, which allows the magnet to maintain the superconducting state, using a high-stability power supply and a helium compressor.

## 8 Summary and Outlook

Knowledge of carbohydrate structure forms the basis of understanding glycan function in biology and medicine. The developments of (i) NMR pulse sequences improving speed of experiments, (ii) hardware enhancing signal-to-noise ratio, and (iii) magnetic field strengths surpassing 1 GHz, thereby increasing spectral resolution, have during the last two decades materialized such that tools for liquid-state NMR spectroscopy are available to efficiently elucidate glycan structure of highly complex oligo- and polysaccharides as well as of glycopeptides and glycoproteins. As reviewed herein, the advancements have led to considerable progress in the field of biomolecular systems containing glycans, exemplified by the progression that has taken place since the turn of the century up to today’s state-of-the-art NMR technologies. Complementary to this, strategies for NMR spectral analysis of oligosaccharides and carbohydrate polymers have been described using specific examples in a tutorial way,^472,473^ illustrating the wealth of information available from 1D, 2D, and 3D NMR experiments, whereby chemical shifts and spin–spin coupling constants can be obtained and connectivities in and between sugar residues may be established.

Future developments in structural analysis of glycans will include the use of machine learning techniques to predict NMR chemical shifts from structure or vice versa to determine the structure of carbohydrates from NMR spectra. Machine learning methods related to NMR spectroscopy are to this end presently being developed based on data-driven approaches^474^ and density functional theory quantum chemical computed values^475−477^ of organic molecules as well as by using deep neural networks (DNN) for peak picking of biomolecular NMR spectra.^478^ NMR spectroscopy was employed in conjunction with supervised machine learning models, which map input data and via an inferred function produces output data to detect in an automatic fashion adulteration in honey, such as invert sugar, i.e., hydrolyzed sucrose.^479^ The classification methods included a logistic regression classifier, DNN, and a light gradient boosting machine; interestingly, by combining the results through a voting method using all of the classifiers, the tested data sets were correctly identified, whether they came from samples containing adulterated or pure honey. One can foresee that machine learning approaches will have great potential to complement already existing NMR chemical shift prediction methods based on increment rules (vide supra, section 6.2).

The post-translational modification of proteins by glycans may take place by multiply O-glycosidically linked *N*-acetyl-d-galactosamine residues^480^ or by larger complex oligosaccharides.^481^ Chemical shift displacements upon glycosylation of peptides and proteins, monitored by, e.g., ^1^H,^15^N-HSQC^480^ or ^1^H,^1^H-TOCSY^482^ NMR experiments, can be utilized as specific identifiers on sites of modification and the process of sequential addition of glycans to the polypeptide chain. The importance of glycosylation in biochemical systems will in future studies be further unraveled by the detailed analysis of the interplay between glycans and polypeptides, where NMR spectroscopy will play an essential role. NMR chemical shift assignments of glycans in large glycoproteins such as antibodies and glycan-substituted Fab fragments thereof or of multiply glycan-substituted proteins in general are highly challenging problems to be solved, whether it be by selective mutation of N- or O-linked positions or complemented by stable isotope labeling. Not only will stable isotope incorporation of ^13^C and/or ^15^N nuclei, in particular, as uniform, site/residue specific, or sparse labeling^157,369,483,484^ lead to enhanced sensitivity in detecting glycan resonances, but metabolic aspects and biosynthesis pathways can also be investigated effectively.

Aided by increased spectral resolution from ultrahigh field NMR spectrometers and specific ultraselective excitation of resonances in crowded spectral regions, NMR experiments will be able to unravel and identify different sugar residues in polysaccharides, i.e., those from the reducing end, primer–adaptor region if present, backbone constituents and terminal end entities, in conjunction with spacing between and partial substitution of side-chains, all of which can give valuable knowledge about biosynthesis. Insight into structure and biosynthesis increases the potential of being able to interfere with polysaccharide assembly, which may help in combatting pathogenic bacteria in general and antimicrobial resistance in particular. Whether it may be the development of experiments based on novel NMR pulse sequences, further enhancement of sensitivity or continued advancement of ultrahigh field magnets, solution-state NMR spectroscopy will be vital for successful research on glycans.
